# People calibrate future expectations to past performance when predicting transparently random events

**DOI:** 10.1093/pnasnexus/pgaf237

**Published:** 2025-08-26

**Authors:** Russell Roberts, Reid Hastie, Alexander Todorov

**Affiliations:** Booth School of Business, University of Chicago, Chicago, IL 60637, USA; Booth School of Business, University of Chicago, Chicago, IL 60637, USA; Booth School of Business, University of Chicago, Chicago, IL 60637, USA

**Keywords:** randomness, prediction, rationality, risk, luck

## Abstract

We report on five experiments studying people's (*n* > 12,000) responses to a prototypical random process: predicting the outcomes in a sequence of five fair coin tosses. “Success” rates in making predictions followed the binomial distribution, and randomly assigned participants to zero to five success experiences, capturing the entire distribution of possible performance outcomes without deception. We found that more successful predictions led to more optimistic expectations of future performance and an increased propensity for risk-taking behaviors, whereas more unsuccessful predictions led to more pessimistic expectations and risk-averse behavior, demonstrating the tendency to believe that there is a signal in performance predicting random sequences of events. Inference from performance was stronger for participants who changed their predictions more often, suggesting that it is more likely to emerge when participants detect a spurious correlation between their behavior and the experienced outcomes. The findings could not be explained by distorted beliefs about the nature of the outcome-generating process, poor knowledge of probability, or risk attitudes, and were unaffected by the presence of performance-related rewards.

Significance Statement Many popular accounts claim that people fail to accurately assess the role of chance in human experience. Existing evidence for how luck can affect consequential outcomes comes from simulation studies and observational data, but there are no strong experimental tests of this claim in a nondeceptive, transparently random prediction task. Understanding how people respond to performance determined by chance is fundamental for understanding human psychology, with implications for fairness, compensation, and welfare policies. Our experiments show that even when performance is transparently randomly determined, people's expectations of future performance, willingness to take risks, and outcome attributions are all systematically affected by their prior lucky successes and unlucky failures.

## Introduction

Almost all consequential outcomes of human behavior are affected by random processes (1). Buzzer-beating shots win and lose championships, children born in one neighborhood can face lifelong challenges that do not encumber their neighbors a few miles away, and market vagaries can suddenly reward some firms and punish others for unforeseeable reasons. At the same time, the way that people account for these outcomes can have substantial impacts on compensation, welfare policies, and social justice (2–5). An accurate appraisal of how chance affects outcomes like these is important, yet theoretical arguments, simulations, and empirical findings all suggest that people underestimate the role of chance in human experience (6–9), often drawing the wrong conclusions from “lucky successes” and “unlucky failures” and acting on them in ways that are suboptimal. For example, basketball coaches change strategies more often after close losses than wins (10), CEO pay increases when firm performance improves due to uncontrollable factors (11), and people whose homes remain unaffected during natural disasters by chance are less inclined to take follow-up precautions compared with their neighbors whose homes were damaged (12). To make matters worse, more extreme outcomes, whether positive or negative, are more prone to be influenced by chance processes relative to more moderate outcomes (6, 9, 10).

The difficulty people have in accounting for the role of chance in human life stems from the fact that outcomes that are only *partly* determined by random processes are causally ambiguous. Causal ambiguity in turn produces a host of well-documented psychological biases that can distort judgments and predictions. For example, under these conditions people tend to view past events as more predictable than they were in reality (13, 14), to attribute successes to their own efforts and failure to factors beyond their control (15, 16), and to judge decisions based on the quality of the outcome rather than on the quality of the decision process (17). They also tend to view their own task performance over time as diagnostic of ability (18). Interpreting performance as a signal of ability and a predictor of future performance is in itself not necessarily an error. Causal ambiguity implies the possibility of a contingent relationship between actions and outcomes, even when chance factors are present.

However, there are some domains in which outcomes are determined entirely by chance and in which successfully predicting such outcomes occurs at random. Yet even when predicting outcomes determined by conventional random devices such as lotteries, roulette spins, and coin tosses, people behave in ways that suggest that they believe that performance is repeatable. Stores that sell winning lottery tickets experience a subsequent increase in ticket sales (19), people pay a premium to mimic the bets of previous lottery winners (20), and people pay to see the coin-flip predictions of others who have recently made successful predictions (21). In such transparently random domains, particularly those in which the base rate of each possible outcome is known, past prediction performance should have no influence on people's expectations of future performance because there are no nonchance factors that can affect outcomes.

We created a task designed to minimize the availability of nonchance attributions to study how experiencing success or failure predicting outcomes completely determined by a random mechanical process (coin tosses) affected people's behaviors and judgments. We conducted five large-scale experiments (*n* > 12,000) using a transparently nondeceptive judgment task in which participants predicted a sequence of five fair coin tosses (real coins in experiments 1 and 3, and virtual coins in the remaining experiments). We assigned about 1,000 participants to each experimental condition so that about 30 participants would experience each of the least probable outcomes (five successes or five failures). The binomial distribution of participants experiencing successes, ranging from zero to five, is accordingly 32, 156, 312, 312, 156, and 32. Essentially, participants were randomly assigned to experience different numbers of successes. Thus, we created conditions representing the entire distribution of possible performance in which people experienced real lucky successes and unlucky failures and examined their expectations about future performance.

With respect to prior work on how people respond to personal performance when outcomes are randomly determined, we considered three possible patterns of results. First, people could ignore their own prior prediction performance and forecast future performance in line with the known base rate (e.g. 50% success rate). This describes the behavior of a rational actor, and we refer to this potential pattern as *base rate adherence*. A second possible pattern of results consists of participants forecasting future performance that, when averaged with prior performance, produces the known base rate of random outcomes. We refer to this as *base rate compensation*, a notion consistent with the logic of the gambler's fallacy and also present in so-called “stock of luck” beliefs and belief in the law of small numbers (22, 23). In essence, this pattern would hold if especially lucky people forecast unusually poor performance in the near future and if especially unlucky people forecast unusually good performance. Finally, people could expect their prediction performance, whether good or bad, to persist into the future. We refer to this pattern as *base rate updating* because it entails the belief that the overall probability of success in the task is not equal to 0.5.

For unusually successful performance (but not unusually unsuccessful performance), this third pattern would be consistent with research on effects known as the “illusion of control” and the “hot hand fallacy.” Indeed, in a seminal paper, the illusion of control was defined as “an expectancy of personal probability inappropriately higher than the objective probability would warrant” (24, p. 311). However, none of the measures used in the associated studies captured judgments of the subjective probability of personal success that could be compared against objective benchmarks (24). Subsequent work in this literature moved away from ostensibly random devices, and none examined responses to real outcomes (25–29). Thus, it remains an open question whether judgments would follow this pattern under stricter conditions. Furthermore, theories of illusory control focus on inferences of (spurious) positive correlation between actions and outcomes but have unclear implications about what happens when people detect a spurious negative correlation.

Similarly, although there is a large literature on the hot hand fallacy—the expectation of positive autocorrelation in sequences of binary performance outcomes—the best evidence involving i.i.d. random devices and real outcomes comes from gambling studies that confound reward with performance success and do not measure participants' beliefs (30), or from experiments with instructions that suggest that prediction is possible before the task begins (31). Research on hot hand beliefs is also subtly different from our approach in that it is focused particularly on performance streaks. Our data include all possible levels *and* sequences of prediction performance, and the effects we observe can be statistically distinguished from performance streaks per se.

Given the importance of understanding judgments of random events, we searched for a dataset examining prediction performance of a transparently random device with known base rates producing real outcomes without deception. Although there is a substantial literature of behavioral studies of people's reactions to events generated by random processes (32), most of these studies rely on fictional or deceptive scenarios (33, 34), small samples (35), or samples of gamblers under conditions where beliefs can only be indirectly inferred and where performance and reward are confounded (30). We specifically looked for studies involving predicting the outcomes of real coin tosses, because coins are familiar random devices that produce outcomes with known base rates (other random devices such as lottery tickets and roulette wheels may be less familiar to participants or may have outcome base rates that are less readily known). In a review of over 200 experiments studying judgments of binary events occurring in sequences (see Appendix A), we found only 15 experiments in which the stimulus events were described as coin tosses and only four experiments in which the to-be-judged events were generated by (pseudo) i.i.d. processes with strong stationary prior probabilities. Only one experiment used actual physical coins as stimuli (36), and that study did not report on prediction strategies or the effects of performance on subsequent attributions and inferences. Thus, from both theoretical and empirical standpoints, the effect of prediction performance of transparently random outcomes on people's beliefs and behavior is less well-established than it may first appear.

Across experiments, we measured participants' beliefs about their ability to predict future coin tosses, how they expected to perform on 20 future coin toss predictions, their attributions of performance to skill and luck, and their incentivized risky bets on future predictions. In experiments 1 and 5, all measures were collected after the first (and only) five trials in which participants predicted coin toss outcomes. In experiments 2–4, after the first five trials, we measured only risk-taking behavior (a one-shot decision on how much risk to take on before predicting the outcomes of five subsequent tosses). To avoid the possibility of responses to other measures (such as ability to predict future tosses and luck/skill attributions) influencing the risk-taking measure, we collected all other measures after the second five trials of the prediction task.

First, we report the results for our main measures: expected future task performance and risk-taking behavior. We find that these measures are systematically and positively related to the number of experienced successes. Second, we test whether the main results are qualified by participants' knowledge of probability, their risk attitudes (experiments 2–4), or beliefs in the randomness of the outcome-generating process (experiments 1–5). Third, we report the results for attributions of prediction performance to skill and luck (experiments 1–5), as well as confidence that the participant's advice would help another participant to do well in the task (experiment 4). Fourth, we test whether the main results are specific to prediction tasks (experiment 5) and whether providing rewards contingent on performance changes these results (experiments 4 and 5). We also analyze how changes in prediction behavior moderate the effect of randomly experienced failure or success on our measures. Finally, we discuss the observed results in the context of related phenomena such as the hot hand (37) and illusion of control (24–29).

## Results

### Expectations about future performance and risk-taking behavior

**Fig. 1.**
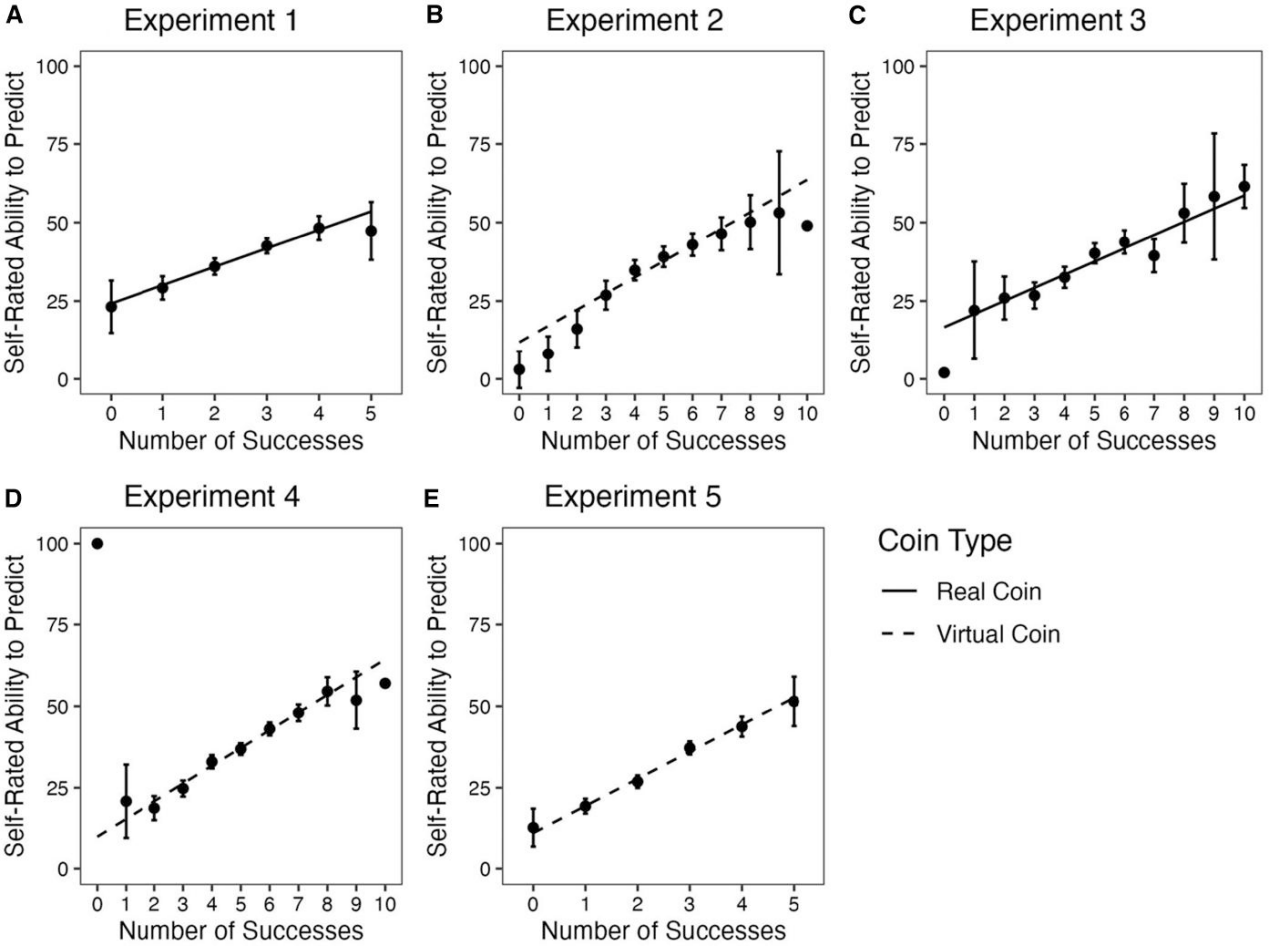
Perceived ability to predict future coin tosses as a function of experienced lucky success. Participants rated the degree to which they could accurately predict the outcomes of future coin tosses on a sliding scale from “No ability” to “Ability to predict outcomes perfectly.” Participants rated their ability after five predictions in experiments 1 and 5 and after 10 in experiments 2–4. A) Experiment 1 (*n* = 998), real coin. B) Experiment 2 (*n* = 1,001), virtual coin. C) Experiment 3 (*n* = 988), real coin. D) Experiment 4 (*n* = 3,001), virtual coin; the data are aggregated across three reward conditions, which did not interact with the effect of success. (Figures for each condition are given in the Supplementary material.) E) Experiment 5 (*n* = 2,010), virtual coin; data are from the prediction condition aggregated across two reward conditions, which did not interact with the effect of success. (Figures for each condition are given in the Supplementary material.) Individual points without error bars indicate that just one participant experienced these outcomes. Error bars are 95% CIs.

In all studies, we used two measures of expected future task performance: participants' self-rated ability to accurately predict the outcomes of future coin tosses and their estimates of how many out of 20 hypothetical additional tosses they expected to predict accurately. A fully rational actor's judgments of expected performance should show *base rate adherence* when performance is randomly determined—that is, they should be insensitive to their experience of success or failure. However, both measures were sensitive to the number of experienced successes. This sensitivity was not characterized by an expectation of mean reversion (*base rate compensation*) but instead revealed a pattern consistent with *base rate updating*. As shown in Fig. 1, the more (lucky) successes participants experienced, the more optimistic they were about future performance, whereas the more failures they experienced, the more pessimistic they became (*b*_Experiments 1–5_ = 4.20–8.31, *t*_Experiments 1–5_ = 7.99–18.57, *P*_Experiments 1–5_ < 0.001). This effect was robust across five separate experiments and was not sensitive to other manipulations such as outcome-dependent rewards, as described below. Although this particular measure is on a subjective scale, making the normative interpretation ambiguous, the participants' ratings nevertheless should not have been sensitive to task success.

**Fig. 2.**
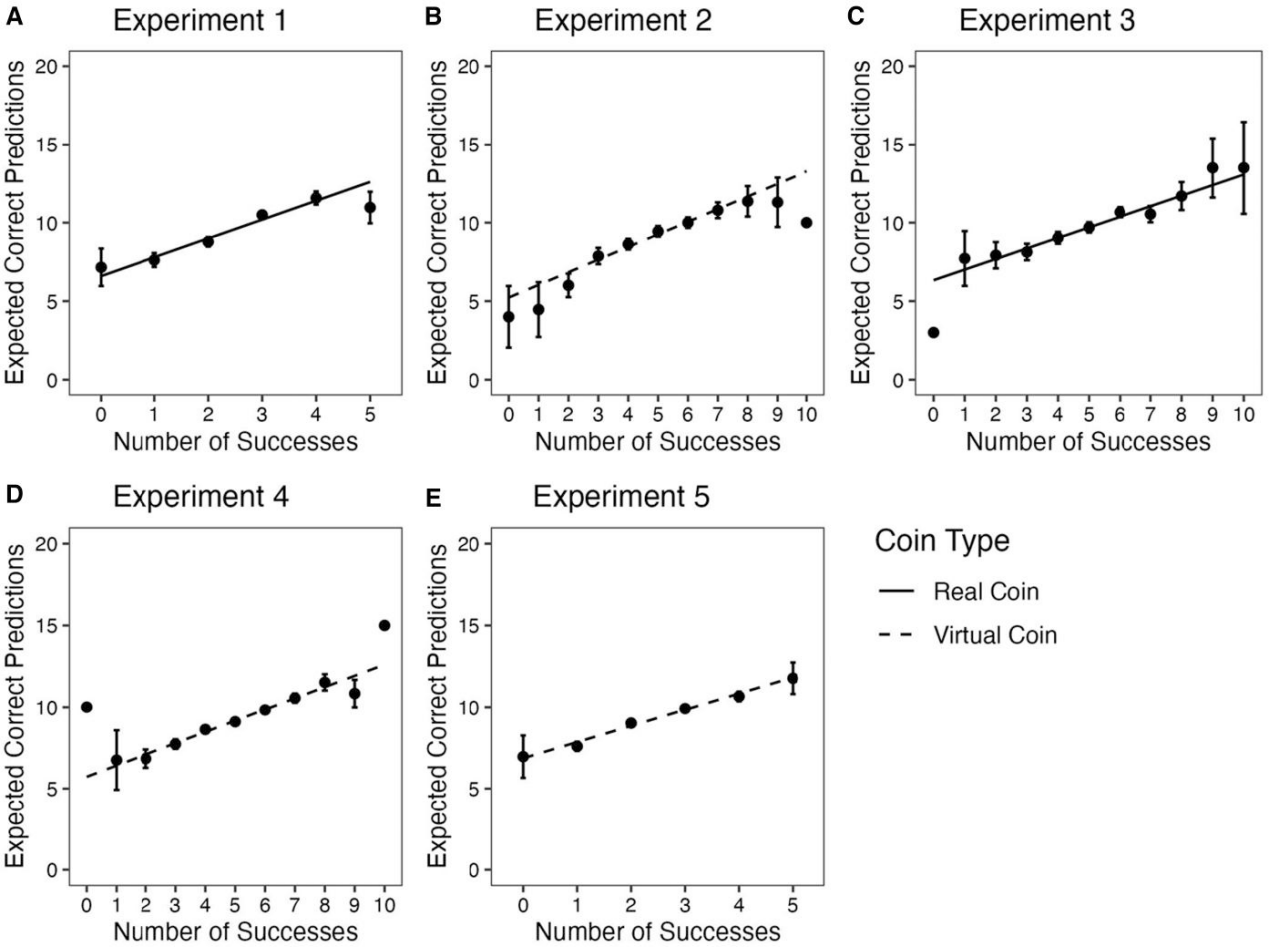
Number of expected correct predictions out of 20 as a function of experienced lucky success. Participants forecasted how many predictions out of 20 they would get correct after five predictions in experiments 1 and 5 and after 10 in experiments 2–4. A) Experiment 1 (*n* = 998), real coin. B) Experiment 2 (*n* = 1,001), virtual coin. C) Experiment 3 (*n* = 988), real coin. D) Experiment 4 (*n* = 3,001), virtual coin; the data are aggregated across three reward conditions, which did not interact with the effect of success. (Figures for each condition are given in the Supplementary material.) E) Experiment 5 (*n* = 2,010), virtual coin; data are from the prediction condition aggregated across two reward conditions, which did not interact with the effect of success. (Figures for each condition are in the Supplementary material.) Individual points without error bars indicate that just one participant experienced these outcomes. Error bars are 95% CIs.

Our second measure is less ambiguous to interpret: expected correct predictions out of 20. Although a perfectly rational actor would expect 10 correct predictions regardless of prior performance, participants' expected number of correct predictions increased nearly linearly as a function of their prediction performance on the initial trials (*b*_Experiments 1–5_ = 0.67–1.20, *t*_Experiments 1–5_ = 12.33–22.02, *P*_Experiments 1–5_ < 0.001; Fig. 2). Our primary focus is the slope of the success curves for each dependent variable, but for measures with a clear normative response, we can examine deviations from the normative benchmark as indicators of overconfidence and under-confidence. The mean number of expected correct predictions for unusually successful participants exceeds 10, but often only by a small margin (Fig. 2). Although this may be described as a small effect, participants' experience challenges a very strong prior. Not only do participants know the base rate of success, but they also know how the outcomes are generated, especially in experiments using real coins. In contrast to unusually successful participants, unusually unsuccessful participants' estimates of accurate predictions tend to fall below 10 with a wider margin, although this asymmetry is more pronounced for experiments using a virtual coin.

**Fig. 3.**
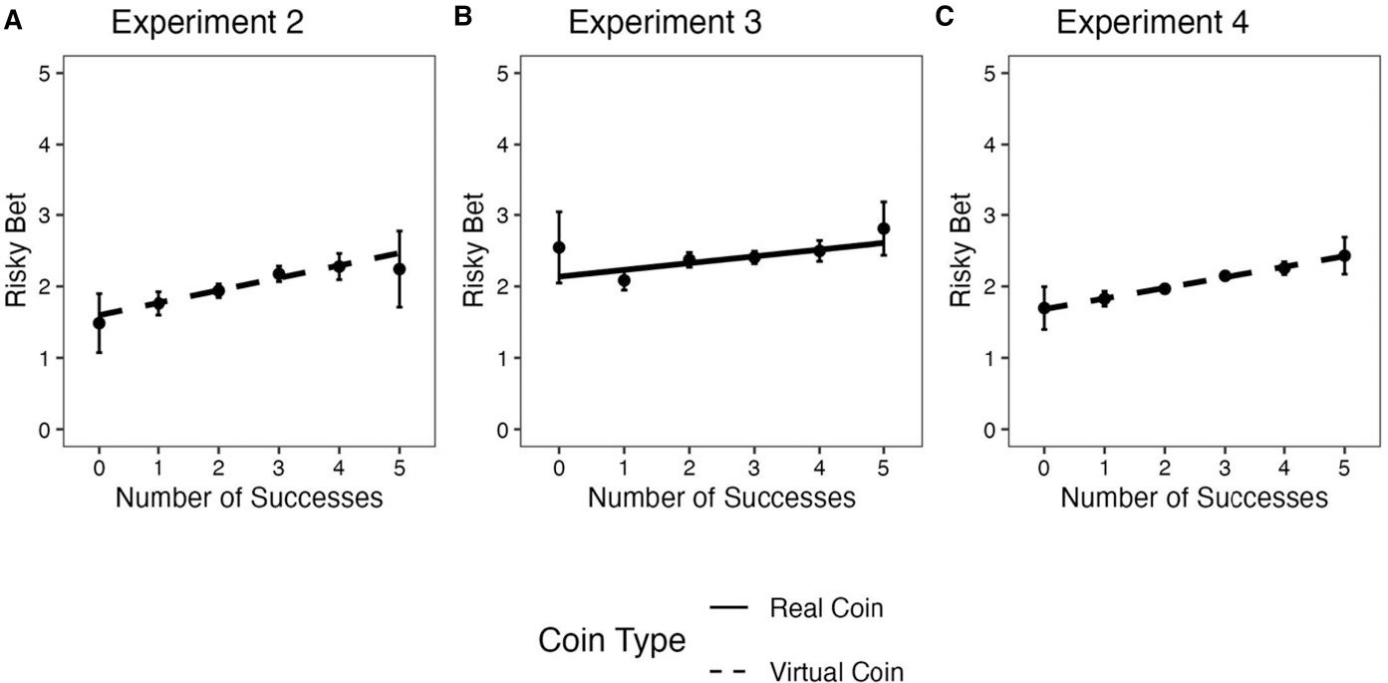
Risky bet on minimum number of correct predictions (out of five) as a function of experienced lucky success. Participants bet on attaining a minimum rate of success, with escalating rewards for greater rates of success. They needed to correctly predict the outcomes of at least as many tosses as they expected to get correct to be rewarded. Payment options were as follows: 25¢ for getting at least zero out of five predictions correct (sure thing); 50¢ for getting at least one out of five predictions correct, $1 for getting at least two out of five predictions correct, with a similar doubling reward schedule up to $8 for successfully predicting five out of five coin tosses. A) Experiment 2 (*n* = 1,001), virtual coin. B) Experiment 3 (*n* = 988), real coin. Participants were rewarded using a lab-based token economy following a similar reward schedule. C) Experiment 4 (*n* = 3,001), virtual coin; the data are aggregated across three reward conditions, which did not interact with the effect of success. (Figures for each condition are in the Supplementary material). Experiments 1 and 5 used different risk measures (Supplementary material). Error bars are 95% CIs.

Experiments 2–4 assessed risk-taking behavior by offering participants the opportunity to bet on a minimum rate of success for five subsequent trials. If they met or exceeded that success rate, they earned a reward. If they failed to meet that success rate, they earned no reward. Before commencing the second five trials, participants in these experiments selected one of the following six payment options: 25¢ for getting at least zero out of five predictions correct (sure thing); 50¢ for getting at least one out of five predictions correct, with a doubling reward schedule up to $8 for successfully predicting five out of five coin tosses. (Experiment 3 used a real coin and rewarded participants using a lab-based token economy following a similar reward schedule rather than $USD.) Across experiments 2–4, initial success predicting coin toss outcomes was associated with increased risk-taking on future trials (*b*_Experiments 2–4_ = 0.10–0.17, *t*_Experiments 2–4_ = 3.71–8.87, *P*_Experiments 2–4_ < 0.001; Fig. 3). Experiment 5 measured self-reported willingness to bet on future coin toss predictions and found a similar pattern (*b* = 3.99, *t* = 5.50, *P* < 0.001). Experiment 1 used a binary measure of risk-taking, but it was not sensitive to the experimental manipulation (Supplementary material).

As with the measure of the expected number of accurate predictions, relative to the normative benchmarks—the expected number of successful predictions (2.5) or the bet with the highest expected value (3)—the effects were asymmetric for the highly successful and highly unsuccessful participants. Lucky participants were not as overly optimistic about their future performance as unlucky participants were overly pessimistic. Taken together, these findings suggest that in the context of a prediction task where outcomes are transparently randomly determined, the consequences of *unlucky failure* on beliefs and risk behavior are potentially greater than the consequences of lucky success. This asymmetry was more pronounced for participants predicting the virtual coin than those predicting the real coin, suggesting that it may be driven by skepticism about the mechanism experienced by unlucky participants interacting with a virtual coin. This is consistent with the observed asymmetry in participants' judgments of the randomness of the outcome-generating process for virtual vs. real coins, discussed below.

### Probability knowledge, risk attitudes, and beliefs about the process generating the outcomes

**Fig. 4.**
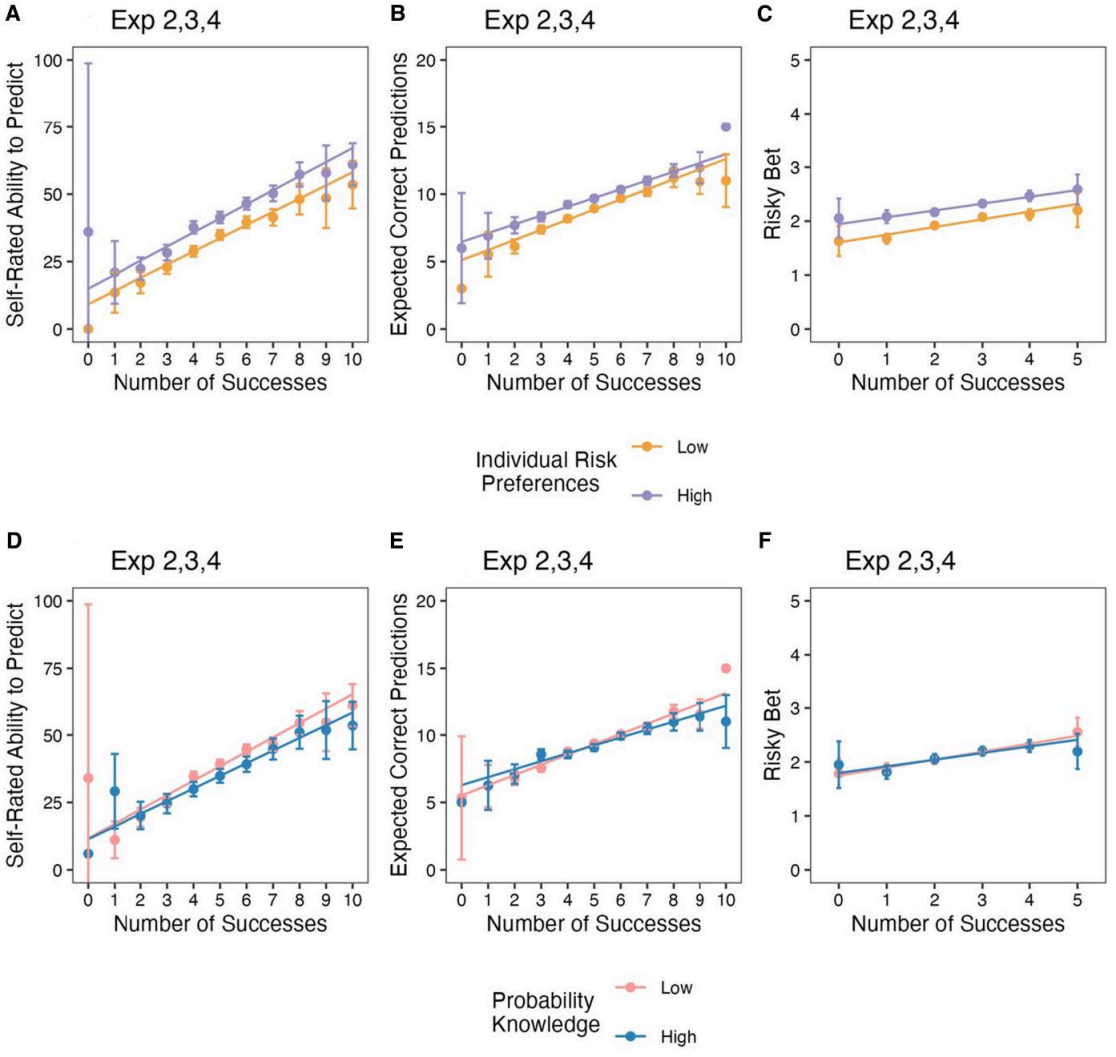
Key dependent variables by individual differences in risk tolerance and probability knowledge. Participants rated their own risk preferences using a single-item measure; a median split was used to display “low” and “high” risk preferences (A–C). Participants completed a four-item measure of probability knowledge (see Supplementary material); data split roughly equally, with “low” probability knowledge depicting three or fewer correct, “high” probability knowledge depicting four out of four correct (D–F). All panels aggregate data from experiments 2–4 (*n* = 4,990). A, D) Self-rated ability to predict future tosses. B, E) Number of expected correct predictions (out of 20). C, F) Risky bet on minimum rate of prediction success (out of five). Individual points without error bars indicate that just one participant experienced these outcomes. Error bars are 95% CIs. Figures and detailed analyses for each experiment are summarized in the Supplementary material.

To test whether the findings described above were driven by participants with poor knowledge of concepts of probability, we measured basic probability knowledge in experiments 2–4. We regressed the main measures on the number of successes, probability knowledge, and their interaction. For perceived ability to predict future tosses, this analysis revealed a negative main effect of probability knowledge (*b*_Experiments 2–4_ = −4.40 to −3.50, *t*_Experiments 2–4_ = 4.01–9.09, *P*_Experiments 2–4_ < 0.001; see Supplementary material for detailed results) but did not reveal a significant interaction. Figure 4 displays these findings as a split between participants with “low” and “high” knowledge. People with greater knowledge of probability tended to be more pessimistic about their ability to predict future coin tosses, but they were similarly affected by prior performance.

For expected number of correct predictions, the analysis revealed a significant negative main effect of knowledge in experiment 2 (*b* = −0.30, *t* = 3.23, *P* < 0.001) and experiment 4 (*b* = −0.24, *t_s_* = 4.47, *P* < 0.001), but not in experiment 3 (*b* = −0.04, *t* = 0.43). For this measure, the analysis also revealed a significant interaction for experiments 2 and 3 (*b*_Experiments 2,3_ = −0.16 to −0.18, *t*_Experiments 2,3_ = 2.75–3.25, *P*_Experiments 2,3_ < 0.006), but not experiment 4 (*b* = −0.06, *t* = 1.83, *P* = 0.07). As shown in Fig. 4E, the most knowledgeable participants were less sensitive to their experience. Nevertheless, their estimates also changed linearly as a function of prediction performance (*b*_Experiments 2–4_ = 0.51–0.62, *t*_Experiments 2–4_ = 6.31–13.20, *P*_Experiments 2–4_ < 0.001).

Risk behavior was largely unaffected by probability knowledge. The only significant effect was the main effect of knowledge in experiment 3 (*b* = −0.07, *t* = 2.33, *P* = 0.02; see Table S25 and Fig. S13). This lack of effects is consistent with studies showing that educational interventions have no effect on gambling behavior, though they can improve skills in computing gambling odds and understanding of gambling fallacies (38). The latter findings are also consistent with the significant interaction for the expected number of accurate predictions in experiments 2 and 3.

In addition to probability knowledge, we also tested whether our results could be driven primarily by participants with unusually high individual preference for risk. We measured risk preferences using a single well-validated item (39), asking participants how willing they are to take risks in general. Although this measure was positively associated with perceived ability to predict (*b*_Experiments 2–4_ = 0.13–0.18, *t*_Experiments 2–4_ = 3.89–10.68, *P*_Experiments 2–4_ < 0.001), expected number of accurate predictions (*b*_Experiments 2–4_ = 0.01—0.02, *t*_Experiments 2–4_ = 4.26–11.13, *P*_Experiments 2–4_ < 0.001), and risk-taking (*b*_Experiments 2–4_ = 0.01, *t*_Experiments 2–4_ = 5.48–9.69, *P*_Experiments 2–4_ < 0.001), it did not significantly impact the effect of prediction performance on these key dependent measures. Figure 4 displays these findings as a median split between participants with “low” and “high” individual risk preferences.

**Fig. 5.**
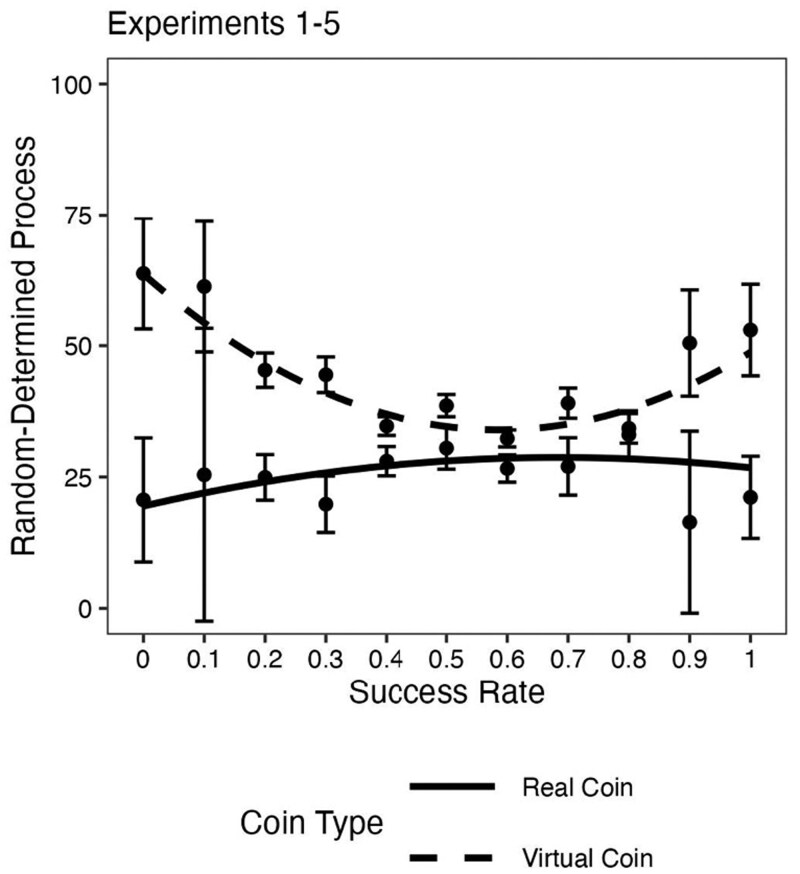
Beliefs that coin toss outcomes resulted from Random (0)–Determined (100) process by rate of success and coin type (Real vs. Virtual). Data are aggregated across experiments 1–5. Success rates are computed because across studies, participants rendered their judgments after either five trials (experiments 1 and 5) or 10 trials (experiments 2, 3, and 4). For experiment 4, this result is averaged over the three levels of the reward contingency variable (no bonus, contingent bonus, and noncontingent bonus). For experiment 5, this result is drawn from the subset of participants who experienced the predict condition (*n* = 2,010) and is averaged over the two levels of the reward contingency variable (contingent bonus and noncontingent bonus). Error bars are 95% CIs. Figures and detailed analyses for each experiment are summarized in the Supplementary material.

Finally, although there was no deception and the coin was fair in all experiments, it is possible that participants did not believe the generation process was random and this distorted their beliefs and behaviors (40). At the end of all experiments, participants rated the extent to which outcomes were random or determined. In the virtual coin toss experiments (experiments 2, 4, and 5), participants who experienced extreme outcomes were more likely to rate the outcomes as determined rather than random. However, this was not the case in the experiments using a real coin (experiments 1 and 3; see Fig. 5). These findings suggest that participants viewed the outcome-generating process for the virtual coin differently than for the real coin. This is reasonable, as online participants may correctly believe that a virtual coin would be easy to manipulate by the experimenter—something that would be much harder to accomplish with a real coin that the participant holds and tosses. Importantly, effects of performance on expected future performance and risk-taking emerged for both real and virtual coins. Whatever differences participants perceived between these two random devices, their responses on self-rated ability to predict future outcomes, expected number of accurate predictions, and risk-taking behavior followed a similar pattern. An interesting observation in the virtual coin experiments was that the U-shaped function was asymmetric: extremely unlucky participants were less likely to believe that the process was random than extremely lucky participants, consistent with a self-serving attribution bias (41).

### Attributions of performance and confidence in advice

Although our primary focus was beliefs about future performance and willingness to act on those beliefs, we also measured skill and luck attributions. A rational actor whose expectations about future performance reflect *base rate adherence* should attribute randomly generated “successes” and “failures” to chance, and their attributions to luck and to skill should be insensitive to these outcomes. Neither *base rate compensation* nor *base rate updating* logically necessitates specific patterns of sensitivity to performance for luck or skill attributions. For example, someone who experienced lucky success and expects their good performance to continue on future trials (*base rate updating*) *might* believe this results from their greater skill, but they also might believe their performance has nothing to do with skill and instead base their optimistic expectations on persistent luck or on some other factor entirely. These would all produce different luck/skill attribution patterns despite producing the same expectations about future performance. Nonetheless, these measures could still offer clues as to why participants have the performance expectations they do.

Experiments 1 and 5 assessed luck and skill attributions as a trade-off, asking participants to indicate on a sliding scale the extent to which they believed their outcomes were due to “totally luck” or “totally skill.” If anything, participants' outcome attributions shifted away from luck and towards skill as a function of increasing success (*b*_Experiments 1,5_ = 2.17–3.00, *t*_Experiments 2–4_ = 3.36–7.58, *P*_Experiments 1,5_ < 0.001; see Fig. S7 for detailed results). This attribution also correlated with the self-rated ability to predict future outcomes (*r*_Experiments 1,5_ > 0.38, *P* < 0.001; see Table S37).

Because skill and luck need not be perceived as opposites (23, 42), experiments 2–4 assessed these attributions separately, asking participants to indicate the extent to which skill (or lack of skill) and luck (good luck or bad luck) played a role in their coin prediction performance. Increasing success was associated with greater luck attributions (*b*_Experiments 3,4_ = 1.21–1.83; *t*_Experiments 3,4_ = 2.48–3.27, *P*_Experiments 3,4_ < 0.013), although the effect was not significant in experiment 2 (*b*  *=* 0.60, *P* = 0.36). Increasing success was also associated with greater skill attributions (*b*_Experiments 2,4_ = 2.55–3.03; *t*_Experiments 2,4_ = 5.20–10.60, *P*_Experiments 2,4_ < 0.001), although the effect was not significant in experiment 3 (*b*  *=* 0.81, *P* = 0.11). Skill attributions were positively correlated with self-rated ability to predict (*r*_Experiments 2–4_ > 0.34, *P* < 0.001; see Table S36), but luck attributions were either uncorrelated or weakly correlated with this measure (range of *r* = 0.01 to −0.11; Table S36). Thus, across experiments, more successful prediction performance was associated with increased attributions to skill.

In experiment 4, we asked participants to write down their advice about “how to accurately predict as many coin tosses as possible,” and then measured how confident they felt that someone else following that advice would be successful in the task. As with our other measures, success in the task was associated with increased confidence in task advice (*b* = 3.32; *t* = 11.46, *P* < 0.001), suggesting that people who predicted random events accurately believed that they could convey information that would help others replicate their success (Table S19).

### Generality of effects

In order to explore the generality of our effects, in experiment 5, we manipulated the nature of the task: the degree to which participants perceive a direct or indirect link between their actions and successful outcomes (43). We created two alternative versions of the task in which participants were paid for “heads” outcomes. In the “observe” condition, participants flipped a coin and observed the outcome. In the “choose” condition, participants chose between two coins to be flipped and observed the outcome. We hypothesized that these two conditions would reduce the extent to which participants' beliefs about prediction ability were sensitive to the rate of experienced success in the task. Across our dependent variables, participants' expectations about prediction performance were sensitive to the number of successes when making predictions but much less so when choosing between two coins and least of all when flipping a single coin without making predictions (e.g. for expected number of correct predictions, *b*_predict_ = 4.02, *t*_predict_ = 5.56, *P*_predict_ < 0.001; *b*_choose_ = 0.29, *t*_choose_ = 0.40, *P*_choose_ = 0.688; *b*_observe_ = −0.91, *t*_observe_ = 1.28, *P*_observe_ = 0.201; see Tables S3 and S6 for detailed analyses).

However, we also measured participants' expectations of how many “heads” they expected to occur on 20 hypothetical subsequent trials. For these two additional experimental conditions (“observe” and “choose”), participants' expectations of future performance (now characterized as “heads” outcomes) were positively associated with the number of experienced “heads” outcomes producing a starkly similar pattern of *base rate updating* relative to predictors' expectations of future predictions (*b*_observe_ = 0.87, *t*_observe_ = 19.83, *P*_observe_ < 0.001; *b*_choose_ = 0.87, *t*_choose_ = 19.29, *P*_choose_ < 0.001). Notably, sensitivity to performance for this measure was statistically indistinguishable between the “observe” and “choose” conditions, suggesting that merely having the option to choose between coins did not further enhance the effect (Table S12) (44). Although these conditions were only tested using a virtual coin, behavioral evidence from experiment 3, in which participants predicted the outcomes of real coin tosses, shows that the number of “heads” outcomes participants experienced during the first five trials was positively associated with how many “heads” outcomes they predicted during the second five trials (*b* = 0.30, *t*(985) = 7.13, *P* < 0.001). Similar trends were observed for participants predicting virtual coin tosses in experiment 2 (*b* = 0.26, *t*(999) = 6.09, *P* < 0.001) and experiment 4 (*b* = 0.24, *t*(2,996) = 9.25, *P* < 0.001).

In addition to our main manipulation of the number of randomly experienced successes, we tested whether escalating monetary rewards contingent on (the first five) successful trials (experiments 4 and 5) would increase the effect of lucky successes on anticipated future performance and risk-taking behavior. In experiment 4, participants were randomly assigned to one of three conditions. In the contingent bonus condition, participants were rewarded for successful predictions starting with $0.01 for a single correct prediction and escalating by a factor of five for each subsequent correct prediction with a maximum reward of $6.25 (five correct predictions). In the noncontingent bonus condition, participants earned a randomly determined bonus following the first five trials drawn from the same probability distribution as in the contingent bonus conditions with equivalent rewards unrelated to the number of successful predictions. In the baseline condition, similar to experiments 2 and 3, participants did not earn a bonus. We measured risk behavior after the initial five trials and then played out the second five trials (without rewards of any kind) before measuring our other dependent variables. In experiment 5, participants were randomly assigned to receive either contingent or noncontingent bonuses for successful predictions (there were only five trials in experiment 5). Contrary to our expectations, we failed to observe significant effects of reward contingencies (see Supplementary material).

**Fig. 6.**
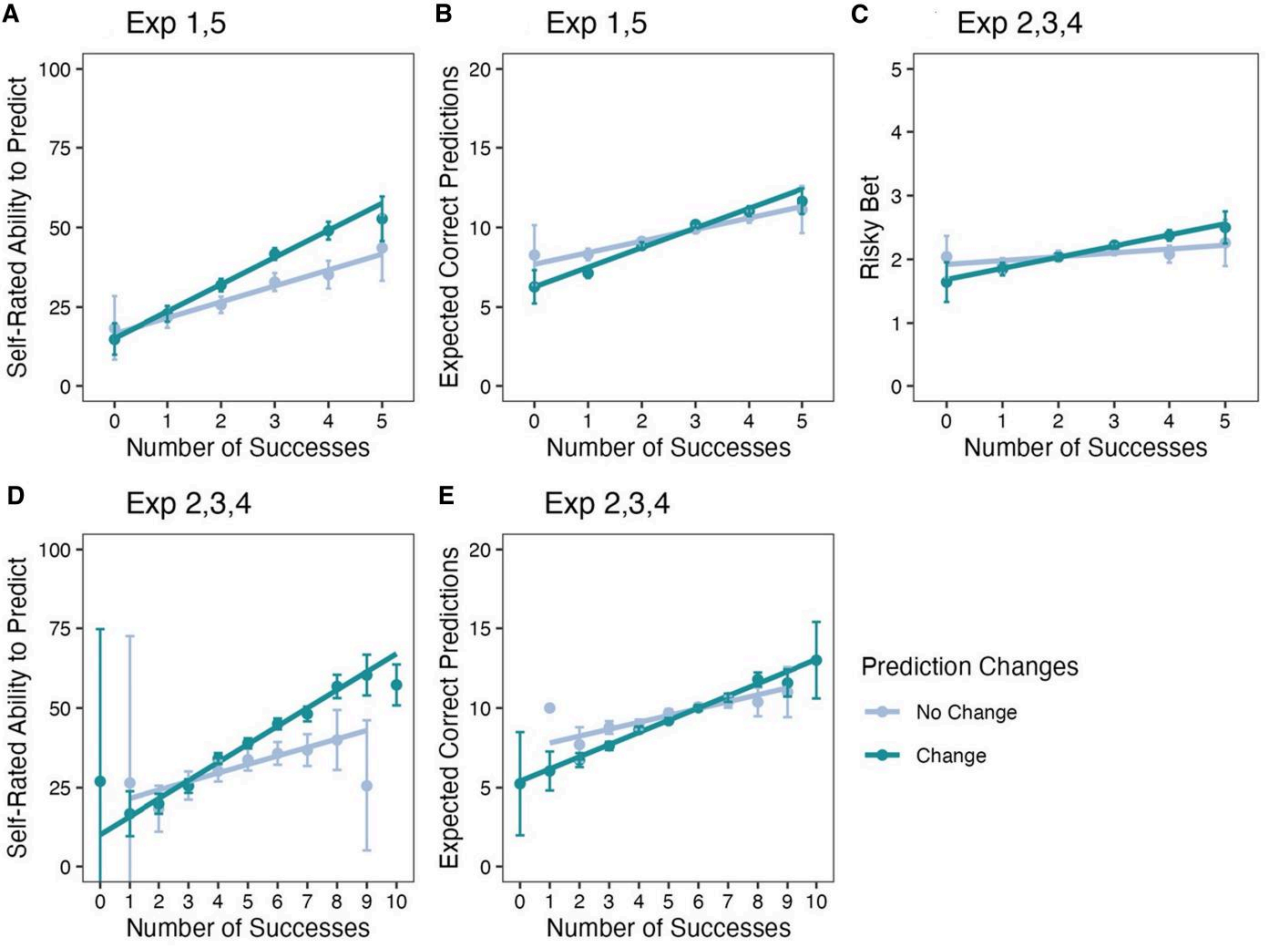
Key dependent variables by changes in coin toss predictions (change vs. no change). Participants who changed their prediction one or more times (change) or who did not change their predictions at all (no change). A) Perceived ability to predict future tosses, experiments 1 and 5 (*n* = 3,008); for experiment 5, data are from the prediction condition. B) Number of expected correct predictions (out of 20), experiments 1 and 5 (*n* = 3,008). C) Risky bet on minimum rate of prediction success (out of five), experiments 2–4 (*n* = 4,990). D) Perceived ability to predict future tosses, experiments 2–4 (*n* = 4,990). E) Number of expected correct predictions (out of 20) experiments 2–4 (*n* = 4,990). Individual points without error bars indicate that just one participant experienced these outcomes. Error bars are 95% CIs. Figures and detailed analyses for each experiment are summarized in the Supplementary material.

Finally, we examined the sequences of predictions produced by each participant to understand how trial-by-trial behavior affected their sensitivity to their own performance. Consider participants in our prediction task who always made the same prediction (e.g. “heads”) and those who made more complex sequences of predictions. A correlation between behavior (varying predictions) and outcomes will appear stronger for the latter than the former (and technically could only be computed for the latter). Indeed, we observed that the effect of prediction performance on beliefs about ability to predict and risk-taking behavior was moderated by the complexity of predictions. For ease of presentation, Fig. 6 shows the results for the groups of participants who forecast the same outcome for all tosses vs. those who changed their forecast at least once. The effect of prediction performance was stronger for participants who made different predictions for beliefs about ability to predict, number of expected correct predictions, and risk-taking behavior. The results hold for more sophisticated measures of algorithmic complexity of predictions (45, 46) (see Supplementary material).

## Discussion

### Random events and base rate updating

These results demonstrate a systematic tendency, even under conditions in which outcomes are entirely and transparently generated by random processes, for people to treat their own prior performance as a signal of expected future performance. Rather than ignoring experience and forecasting base rate performance (*base rate adherence*) or expecting their prior performance to be balanced out by the next few trials (*base rate compensation*), people adjusted their expectations to align with their experience (*base rate updating*). Participants experiencing unlikely success were overconfident in their beliefs and engaged in riskier behavior, whereas those who experienced unlikely failure were unreasonably pessimistic about future performance and bet more conservatively.

Moreover, this pattern of results emerged independently of a number of factors that might be expected to moderate it. For example, neither higher risk tolerance nor the presence of performance rewards enhanced people's sensitivity to their own performance. Likewise, greater knowledge of probability did not attenuate the pattern. Even participants who demonstrated understanding of the odds of success and the independence of random events seemed unable to resist the influence of their prior performance when forecasting future performance and taking risks based on that performance. One factor that did moderate the effect of prior performance on expectations of future performance was participants' own behavior. That is, participants who produced more complex sequences of predictions tended to be more sensitive to their performance relative to those who predicted the same outcome every time. This finding likely reflects the fact that the former participants can more easily see a spurious correlation between their behavior and the outcomes they experience (47).

We also document an asymmetry between the effects of experienced failure and success relative to the objective benchmarks. Participants tended to adjust their expectations more to failure than to success, even when tossing a real coin themselves. This highlights the relatively large impact of spurious failure feedback and has implications for domains such as learning, hiring, and goal pursuit. The perils of overconfidence following lucky success are prominent in modern treatments of underappreciating the role of chance (3, 9, 48), but the current evidence suggests that pessimism about the future following unlucky failure may be even more psychologically potent.

Although we focused on prediction performance, nearly identical expectations emerged when participants tossed coins with the goal of producing heads outcomes, a context in which one might expect a pattern of *base rate compensation*. Despite the fact that we did observe local gambler's fallacy effects on a trial-by-trial basis in the prediction task, people in general tended to forecast higher rates of “heads” outcomes after experiencing more “heads” outcomes in the task. The prediction behavior of participants in experiments 2–4 between the first five trials and the second five trials confirms this—more observed heads on the first five trials was associated with more “heads” predictions on the second five. Thus, *base rate updating* in the context of transparently randomly determined outcomes does not appear to be limited to prediction performance.

Finally, we note that although experiencing rarer events led to more extreme updating, the tendency to adjust expectations in accordance with prior performance was not limited only to the most extreme levels of performance. Even participants experiencing rates of success *slightly* above or below the base rate adjusted their expectations. This suggests that although outlier experiences may lead to beliefs and behavior that deviate furthest from normative expectations, updating also occurs for intermediate deviations from average performance. People who experience more probable outcomes and then forecast performance that aligns with normative benchmarks may be just as unduly influenced by their experience as their lucky and unlucky counterparts—their beliefs and behavior may simply appear more rational because they happen to experience outcomes consistent with what would be most likely to occur by chance.

### Relation of findings to prior research

Within the judgment and decision-making literature, our findings are related to two extensively studied phenomena: the hot hand and the illusion of control. The hot hand fallacy refers to the tendency, under some conditions, to overreact to streaks of successes (and failures) and predict that successes will “persist” or repeat when they do not. Most claims of hot hand judgment errors reference judgments of intentional actions in goal-directed achievement tasks (e.g. (33, 49); but see (50) for a compelling critique of many of the original demonstrations). Similar effects have been demonstrated in judgments of random events (e.g. (40); note that these experiments relied on descriptions of fictional sequences, not actual random sequences). What is distinctive about the present studies is that participants made judgments of familiar and actual mechanical devices (tossed coins) and held strong prior beliefs in the base rate of the outcomes (50–50).

Our experiments were designed to test for the effect of success rates, and these rates bound potential streaks of successes (for example, everyone who experienced five out of five successes also had an ending streak of five). Although these two variables are not orthogonal, we can statistically control for “hot hand” effects (ending streaks of successes) to identify the effect of success per se. We tested for the presence of “hot hand” effects in our data by computing the length of each participant's ending streak of successes (zero to five) and including this variable in our models in addition to the overall number of successes. For all dependent measures in which measurement occurred after five trials, we tested whether controlling for effects of ending streak (i.e. “hot hand” effects) affected the success effects described above. Across all studies and all variables, success effects remained significant when controlling for the effects of ending streaks, while effects of ending streaks were nonsignificant (see Table S40 for full regression model results). The one exception was that experiment 1 revealed a significant *negative* effect of ending streak, a “stock of luck” effect (51), on expected correct predictions (it did not affect the significant positive effect of number of successes). Because it was not repeated across other measures and experiments, we are reluctant to infer anything from this single result.

The present results are also broadly consistent with research on the illusion of control, the belief that one has influence over events even in the absence of any causal connection between actions and outcomes (24–29). Indeed, one of the earliest tests for an illusion of control involved predicting coin toss outcomes and subsequently rating skill and forecasting future task performance as a function of whether prediction “success” was manipulated to appear early or late in the prediction sequence (52). With respect to our findings, we note three things about the illusion of control literature. First, there are no studies in this literature that directly examine the effects of success rate on expectations of future performance. Second, although the illusion of control is often described as the belief that one has control over chance outcomes, very few experiments place participants in a context in which outcomes are ostensibly generated by chance. Those that do use deception and have proven difficult to replicate over the last 50 years (53–57). Third, many of the published studies in the literature are severely underpowered, and there is a large dependence of the observed effect sizes on the respective sample sizes (see Supplementary material and Fig. S20), suggesting either the presence of methodological biases (including publication bias) or that the illusion of control label encompasses several weakly related phenomena.

In addition, we observe a nearly perfectly linear relationship between prediction performance and expected future performance that includes both overconfidence following success and undue pessimism following failure. Theories of illusory control are premised on people detecting a spurious correlation between their actions and desired outcomes (43). Yet our data show that people are at least as sensitive to detecting correlations between their behavior and *undesired* outcomes—an effect largely ignored in the illusion of control literature. Based on this evidence, we propose that the illusion of control represents just one facet of a more general phenomenon in which people adjust their expectations of future performance to accord with prior performance—even when that performance is determined by chance processes.

## Conclusion

Humans seek structure in their environments and capitalize on cause–effect relationships to maximize their adaptive success. But people often perform suboptimally in situations where relationships between events are best described as random or due to chance. We report on five experiments with >12,000 participants judging chance outcomes that provide a rigorous and unequivocal demonstration of the tendency to believe that there is a signal in performance predicting transparently random events. Although the role of chance was unambiguous, (as the outcomes were determined by a quintessentially random device, coin tosses), participants consistently demonstrated the tendency to expect future performance to resemble past performance and acted on those expectations by taking risks in proportion to those expectations.

This effect was not impacted by risk attitudes, probability knowledge, or even the presence of performance rewards but was enhanced when participants alternated their predictions more often. Failure affected judgments to a greater degree than success did, and the trend was present across the entire distribution of possible performance—even small differences in performance close to the expected base rate led to slightly divergent expectations. Taken together, these findings demonstrate that past experience, even when events are randomly determined, exerts an irresistible influence on people's future expectations.

Although many domains that bear on human achievements are affected by random factors, very few are entirely determined by chance. Accordingly, people have great difficulty comprehending the role that chance plays in the outcomes that matter to them. These findings suggest that even when the role of chance is clear and that nothing else can account for their experience, people simply cannot help but use the past, good or bad, as a guide for the future.

## Materials and methods

### Experiment 1

Experiment 1 was conducted after experiment 5. In the latter, we found that as the number of successes increased, participants' willingness to bet on their predictions increased too. In experiment 1, we used a binary measure of risk behavior and hypothesized that greater success in the task would be associated with greater risk-taking.

#### Participants

We recruited a total of 998 participants, *M*_age_  *=* 31.8 (395 males; 573 females; 29 other/prefer not to say) at a public-facing research lab in the downtown district of a major Midwest US city. The sample was 48.4% White, 7.3% Black, 19.7% Asian, 10.4% Latinx, 0.1% Native American, and 24.5% of the participants selected “other” or some combination of options when asked to report their race/ethnicity.

#### Procedure

Participants were told that they would predict the outcomes of five coin flips (heads or tails). Experimenters showed participants the coin (an ordinary US quarter), explained the task, and then elicited predictions. The participant performed each coin flip. Experimenters observed each and every coin flip and did not report any unusual behavior, including attempts to control the outcome. Participants were rewarded for successful predictions starting with $0.01 for a single correct prediction and escalating by a factor of five (e.g. $0.05, $0.25, $1.25, $6.25) for each additional correct prediction. After the task, participants answered a series of questions eliciting their perceived ability to predict future flips (“Rate the degree to which you think you could accurately predict the outcomes if you continued to play the coin toss game” and “Suppose that you continue to play the coin toss game for 20 more trials. On each trial you would predict the outcome (head or tail) in advance of the toss. Out of those 20 trials, on how many trials would your prediction be correct?”), risk tolerance (participants were offered the opportunity to play the game again to win up to $6.25 or forgo the game and take $1.00 for sure), and the extent to which they attributed their performance to luck or to skill (“Your performance predicting the outcomes of the coin tosses can be described as a combination of luck and skill. Rate the degree to which you believe you were just lucky (or unlucky) versus the degree to which you are skilled (in this task)”). We also asked participants to rate the degree to which they thought the outcomes from the task were due to a random or deterministic process, how many heads they expected to occur from 20 future flips, and their willingness to bet on heads for 20 future flips. The exact wording of all measures and their analysis is reported in the Supplementary material. Due to lab requirements, participants were paid in Amazon Gift Cards emailed to them rather than direct monetary payment.

### Experiment 2: virtual coins

Experiment 2 utilized a virtual coin-flipping paradigm similar to experiment 1. Specifically, participants in experiment 2 completed a similar coin-flipping task (but without escalating contingent rewards), and then chose a payment structure for the next five trials of the task. They were compensated via direct payment on MTurk. We hypothesized that prediction success would be associated with greater risk-taking.

#### Participants

We recruited a total of 1,001 participants, *M*_age_  *=* 40.7 (406 males; 586 females; nine other/prefer not to say) from Amazon Mechanical Turk. The sample was 73.3% White, 11.3% Black, 5.4% Asian, 6.6% Latinx, 0.6% Native American, and 2.8% selected “other” when asked to report their race/ethnicity.

#### Procedure

Participants flipped a virtual coin (a US quarter). They were told that the virtual coin was “just like a real coin” that displayed a “flipping” animation when clicked and then displayed either a “heads” or “tails” result, where *P*(heads) = *P*(tails) = 0.5. Participants predicted the outcome five times, each time indicating whether the outcome would be “heads” or “tails.” After each flip, the result was displayed, as well as the results of their previous flips. After completing these initial five trials, participants decided how a monetary reward based on five subsequent coin-flip predictions would be determined (note that this is different from the escalating reward used in experiment 1, where participants completed only five trials). Payment options were as follows: $0.25 for getting at least zero out of five predictions correct (sure thing); $0.50 for getting at least one out of five predictions correct, $1.00 for getting at least two out of five predictions correct, with a similar doubling reward schedule up to $8.00 for forecasting and then successfully executing five out of five correct predictions. Failure to meet or exceed the forecasted number of correct predictions resulted in no reward, and there was no additional reward for exceeding the forecast. In this way, participants wagered on their future rate of successful predictions. After selecting a payment option, participants played out the next five predictions and coin flips. They then responded to a series of self-report measures about their experience (similar to those in experiment 1), a battery of questions measuring their knowledge of probability (Table S38), and a single validated measure of propensity to take risks (39) (“How willing are you to take risks in general?”).

### Experiment 3

Experiment 3 replicated the design from experiment 2 using real coins. We hypothesized that prediction success would be associated with greater risk-taking.

#### Participants

We recruited a total of 988 participants, *M*_age_  *=* 32.6 (395 males; 566 females; 27 other/prefer not to say) at a public-facing research lab in the downtown district of a major Midwest US city. The sample was 47.3% White, 7.7% Black, 17.3% Asian, 12.8% Latinx, 0.2% Native American, and 14.7% selected “other” or some combination of options when asked to report their race/ethnicity.

#### Procedure

Participants completed a version of the task from experiment 2 using a real coin. Due to lab requirements, the reward associated with the risk measure was earned points in a token economy that could be exchanged for prizes (e.g. books, water bottles, and pens). One hundred points roughly equated to $1USD, and the payment schedule was similar to the one used in experiment 2, translated into points (50 points for at least zero out of five correct, 100 points for at least one out of five correct, 200 points for at least two out of five correct, etc.). After the task, participants completed the same measures as in experiment 2.

### Experiment 4: reward contingency

In experiment 4, we experimentally manipulated reward contingency to determine whether earning rewards for successful predictions (of the first five trials) affects subsequent risk-taking behavior. We included three conditions: (i) a *no bonus* condition replicating the procedure from experiments 2 and 3; (ii) a *contingent bonus* condition in which participants earned monetary rewards for the first five coin flips according to the reward schedule from experiment 1 (note that in the latter experiment, this manipulation was perfectly confounded with the number of successful predictions); and (iii) a *noncontingent bonus* condition in which participants completed the first five trials without rewards but were then awarded a random payment (unrelated to the success of their predictions) drawn from the same probability distribution and reward schedule as those in the contingent bonus condition.

We hypothesized that prediction success would be associated with greater risk-taking, as in experiments 2 and 3. This experiment was conducted after experiment 5 and given those results we did not have a hypothesis about how reward contingency would affect risk-taking. (As noted below, for experiment 5, we hypothesized but failed to observe that reward contingency would amplify the effects of lucky success on the dependent measures.)

#### Participants

We recruited a total of 2,998 participants, *M*_age_  *=* 42.2 (1,312 males; 1,656 females; 30 other/prefer not to say) on Amazon Mechanical Turk. The sample was 74.4% White, 9.7% Black, 7.9% Asian, 5.3% Latinx, 0.3% Native American, and 2.4% selected “other” when asked to report their race/ethnicity.

#### Procedure

Participants were randomly assigned to the no bonus, contingent bonus, and noncontingent bonus conditions. In the no bonus condition, participants completed the same procedure as in experiments 2 and 3. In the contingent bonus condition, participants completed the same procedure, but earned monetary rewards for successful predictions in the first five trials ($0.00 for none correct, $0.01 for one correct, $0.05 for two correct; $0.25 for three correct, $1.25 for four correct, $6.25 for five correct). In the noncontingent bonus condition, participants completed measures identical to those in experiments 2 and 3, but between the first five trials and their risk decision, they were awarded a randomly determined bonus drawn from an identical probability distribution to the contingent bonus condition but unrelated to their performance. For example, participants had a 0.03125 probability of winning $6.25, just as in the contingent bonus condition but this reward was unrelated to the success of their predictions. After the task, participants completed the same self-report measures as in experiments 2 and 3.

### Experiment 5: choice and observation

In experiment 5, we created two alternative versions of the task, one in which participants flipped a virtual coin and were paid for “heads” outcomes, and one in which participants chose between two virtual coins and were paid for “heads” outcomes. We hypothesized that these two conditions would reduce the extent to which participants felt that their behavior contributed to success or failure relative to the prediction condition, consistent with the control heuristic (43). When making a prediction of a binary outcome, it is easy to imagine how an alternative prediction would have yielded a different result (“If I had just picked heads, I would have won”). This form of counterfactual thinking is less vivid in a “choice” task (“If I had just picked the other coin, I *might* have won”), and still less vivid in an “observation” task where nothing about participant behavior could have even potentially led to an alternative outcome. We also manipulated reward contingency (contingent bonus and noncontingent bonus conditions), which resulted in a 3 (task type) by 2 (reward contingency) design. We hypothesized that more successful participants would be more confident in future performance, more willing to take risks, and more likely to attribute their performance to skill rather than luck. Additionally, we hypothesized that these effects would be amplified for participants who could choose between random devices and for participants who predicted outcomes and were rewarded based on their predictions.

#### Participants

We recruited a total of 6,035 participants, *M*_age_  *=* 40.1 (2,633 males; 3,362 females; 39 other/prefer not to say) on Amazon Mechanical Turk. Seven hundred and forty-nine participants failed at least one of two attention checks but were retained in the final sample per the preregistration (similar results are obtained when these participants are removed from the analyses). The sample was 75.7% White, 9.4% Black, 7.2% Asian, 4.8% Latinx, 0.5% Native American, and 2.3% selected “other” when asked to report their race/ethnicity.

#### Procedure

Participants were randomly assigned to one of the six conditions: 3 (task type) by 2 (reward contingency). In the *observe* condition, participants experiencing contingent bonuses were told they would be flipping a virtual coin five times and would be given a bonus based on the number of “heads” outcomes they experienced starting at $0.01 for the first “heads” and multiplying by a factor of five for each additional “heads” result. Participants experiencing noncontingent bonuses were simply told they would observe the outcomes of five coin tosses and were awarded a randomly determined bonus, as described in experiment 4. In the *choose* condition, participants completed a task identical to the *observe* condition except they chose between one of two virtual coins on each trial (labeled “coin A” and “coin B”). For these participants, the coin they selected for each flip was also displayed after each trial. In the *predict* condition, participants predicted as in experiments 1–4.

After the task, participants responded to the same self-report measures from experiment 1, with the exception of the risk item, in which participants indicated preference to hypothetically play the game again to win $1 per success, up to $5, or take $2.50 for sure. This risk preference measure was never played out.

## Supplementary Material

pgaf237_Supplementary_Data

## Data Availability

Materials, data, and analysis code for all studies are available here. Preregistrations for hypotheses, methods, and analyses are available here (experiments 1–5).^a^ Analysis was always conducted after data collection was complete. The authors also note that they first preregistered and conducted experiments 5 and 1, and then experiments 2, 3, and 4. However, they decided to present the experiments in the current order—from the simpler to more complex experiments—for conceptual clarity. All participants provided informed consent and all studies were approved by the University of Chicago Institutional Review Board (IRB21-1166 and IRB22-0638).
