# Supplementary material for: People calibrate future expectations to past performance when predicting transparently random events
Source: PNAS Nexus. 2025 Aug 26;4(8):pgaf237. doi: 10.1093/pnasnexus/pgaf237 (PMC12378910; doi:10.1093/pnasnexus/pgaf237)
Supplement: pgaf237_Supplementary_Data [file pgaf237_supplementary_data.zip › PNASNEXUS-PNASNEXUS-2025-00637-s01.pdf]

People calibrate future expectations to past performance when predicting transparently random events

Supplementary Materials

Table of Contents

I. Summary of Supplemental Analyses and Findings p. 2
II. Results p. 2
  A. Additional Dependent Variable Analyses
    1. Ability to Predict p. 3
    2. Expected Number of Correct Predictions (Out of 20) p. 7
    3. Risk p. 11
    4. Expected Number of Heads (Out of 20) p. 15
    5. Willingness to Bet on Heads p. 17
    6. Luck and Skill Attributions p. 19
    7. Confidence in Advice p. 24
    8. Random-Determined Process Beliefs p. 26
  B. Alternative Explanations
    1. Probability Knowledge p. 30
    2. Individual Risk Preferences p. 34
    3. Affective State p. 36
  C. Moderating Variables
    1. Prediction Complexity p. 37
    2. Prediction Changes p. 43
III. Correlations of Dependent Measures p. 49
IV. Hot Hand Effects p. 51
V. Discussion of Illusion of Control Studies p. 52
VI. Tables with exact wording of all measures p. 54
VII. Appendix A: Reference List of Judgments of Binary Sequences p. 59

Summary of Supplemental Analyses and Findings

Across five experiments and over $N = 12{,}000$ participants, we studied how prediction performance completely determined by a random mechanical process (coin tosses) affects people's behaviors and judgments. Participants predicted a sequence of five fair coin tosses (real coins in Experiments 1 and 3, and virtual coins in the remaining experiments), with their randomly determined number of "successes" serving as our main independent variable. As reported in the main text, we observed a consistent effect of task success on participants' beliefs in their ability to predict future coin tosses and in their willingness to bet on that ability. In what follows, we report on regression analyses examining all our measured dependent variables across all studies, as well as the effects of moderating variables such as prediction changes, prediction complexity, probability knowledge, and individual differences in risk tolerance. We also report two additional measures: expected "heads" outcomes (out of 20) and willingness to bet on "heads" outcomes. The full text of each measure of participant's beliefs is available in *Table S37*, and the full text of each measure of participant's risk behavior/intentions is available in *Table S38*.

In Experiments 1 and 5, participants completed five task trials and subsequently completed all dependent measures. In Experiments 2, 3, and 4, we were primarily interested in risk-taking behavior, which entailed completing another five trials, and measured this item alone after the first five trials. We waited to measure the rest of the dependent measures until after all ten trials in these studies for two main reasons. First, because we would be collecting five additional trials anyway (to play out people's risk-taking decisions), we wanted to factor this additional data into our analyses for the rest of the dependent measures. Second, we did not want to influence people's risk behavior in the second five trials by eliciting their beliefs, particularly about their own abilities and about the nature of the mechanism. Results for each of the dependent measures are consistent across experiments, but it should be noted that for Experiments 2, 3, and 4, the second five trials occurred after participants had made risk decisions and therefore may have had a different orientation towards task outcomes. Given the consistency of the results, we do not have reason to believe this systematically impacted participants' responses.

Supplementary Analyses

Unless otherwise specified, predictors in regression models were always mean-centered. For Experiments 1 and 5, all judgments and decisions were made after five trials. For Experiments 2-4, all judgments came after 10 trials, with risk taking decisions being made after the first five trials. This means that b values represent the predicted change in y that results from one additional successful prediction, which could be out of five (Experiments 1, 5) or ten (Experiments 2, 3, 4). We chose to mean-center but not scale predictors for ease of interpretation.

For all figures, variable means at each number of successes contain 95% error bars. These error bars widen as the number of successes becomes more rare (e.g., for five trials, 0 successes and 5 successes are less common than 2 or 3 successes) and, as a result, the sample of participants experiencing these outcomes decreases. Because fewer participants, by chance, experienced those rarer outcomes, mean estimates are less precise. Additionally, for measurements after ten trials, some estimates contain no error bars (because just one participant experienced that number of successes) or no data at all (because no participants experienced that

number of successes). The rest of this section is organized in subsections for each measure, reporting analyses for all experiments in which the measure was used (see *Tables S7* and *S38*).

Ability to Predict

Self-rated ability to predict future coin tosses was always measured using the same item (*Table S37*), and was consistently positively affected by the number of successful predictions across all experiments (*Table S1*). Experiment 4 found no differences in this variable across *Reward Contingency* conditions (*Table S2; Figure S1)*. Experiment 5 found that participants in the *Predict* condition were significantly more affected by the number of successes than those in both the *Observe* and *Choose* condition, and found no effects of *Reward Contingency* between conditions (*Table S3*, *Figure S2*). However, the simple effect of the number of successes on self-rated ability to predict was significantly greater than zero for participants in the *Choose* condition who experienced *Contingent Bonuses (Table S3)*. This significant simple effect was repeated in one other dependent measure (Number of Expected Correct Predictions out of 20), and was the only statistically significant evidence that choosing between two coins affected participant's beliefs.

Table S1: ***Regression analyses for self-reported ability to predict future coin toss outcomes as a function of number of experienced successes.*** *For Experiment 4, this result is averaged over the three levels of the Reward Contingency variable (No Bonus, Contingent Bonus, Non-contingent Bonus). For Experiment 5, this result is drawn from the subset of participants who experienced the Predict condition (n = 2010) and is averaged over the two levels of the Reward Contingency variable (Contingent Bonus, Non-contingent Bonus).*

Ability to Predict	*b*	95% CI LB	95% CI UB	*t*	*p*
Experiment 1					
(Intercept)	39.22	37.78	40.66	8.96	<.001
Number of Successes	5.87	4.58	7.15	8.97	<.001
Experiment 2					
(Intercept)	37.42	35.85	39.00	46.56	<.001
Number of Successes	5.18	4.18	6.17	10.23	<.001
Experiment 3					
(Intercept)	37.52	35.90	39.13	45.63	<.001
Number of Successes	4.20	3.17	5.23	8.00	<.001
Experiment 4					
(Intercept)	37.48	36.59	38.37	82.38	<.001
Number of Successes	5.43	4.87	5.99	18.92	<.001
Experiment 5					
(Intercept)	31.81	30.72	32.90	57.12	<.001
Number of Successes	8.29	7.31	9.27	16.58	<.001

Table S2: ***Regression analysis for self-reported ability to predict future coin toss outcomes as a function of number of experienced successes and Reward Contingency condition (Experiment 4)****. Reward Contingency conditions dummy-coded (reference condition: No Bonus).*

Experiment 4:<br>Ability to Predict by Reward Contingency	*b*	95% CI<br>LB	95% CI<br>UB	*t*	*p*
(Intercept)	37.69	36.14	39.23	47.78	<.001
Number of Successes	5.48	4.51	6.46	11.00	<.001
Contingent Bonus	-0.54	-2.72	1.65	0.48	.631
Non-contingent Bonus	-0.10	-2.28	2.09	0.09	.931
Number of Successes*Contingent Bonus	0.04	-1.33	1.41	0.05	.957
Number of Successes*Non-contingent Bonus	-0.19	-1.58	1.20	0.27	.785
Simple Effects (Number of Successes by Condition)					
No Bonus	5.48	4.51	6.46	11.00	<.001
Contingent Bonus	5.52	4.56	6.48	11.25	<.001
Non-contingent Bonus	5.29	4.30	6.28	10.49	<.001
Contrasts					
No Bonus – Contingent Bonus	-0.04	-1.71	1.64	0.05	>.999
No Bonus – Non-contingent Bonus	0.19	-1.50	1.89	0.27	>.999
Contingent Bonus – Non-contingent Bonus	0.23	-1.45	1.92	0.33	>.999

Figure S1: ***Self-rated ability to predict future coin toss outcomes by number of experienced successes and Reward Contingency condition (Experiment 4).*** *Error bars = 95% confidence intervals.*

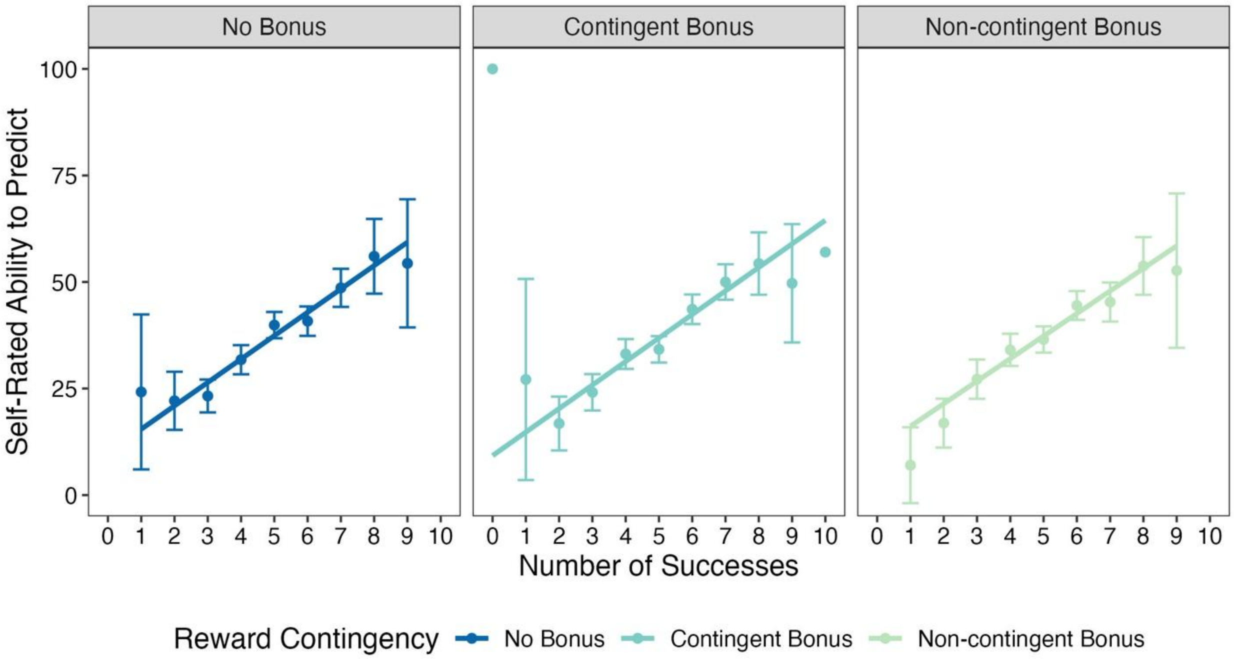

Table S3: ***Regression analysis for self-reported ability to predict future coin toss outcomes as a function of number of experienced successes, Reward Contingency condition, and Task condition (Experiment 5).*** *Reward Contingency conditions dummy-coded (reference condition: Contingent Bonus). Task conditions dummy-coded (reference condition: Observe). Contrasts are Bonferroni corrected for multiple comparisons.*

Experiment 5: Ability to Predict by Condition	*b*	95% CI LB	95% CI UB	*t*	*p*
(Intercept)	30.94	29.31	32.57	37.18	<.001
Number of Successes (NoS)	0.47	-0.99	1.92	0.63	.530
Task: Choose	-4.92	-7.23	-2.62	4.19	<.001
Task: Predict	-0.89	-3.2	1.41	0.76	.447
Reward: Non-contingent (NC)	3.84	1.53	6.14	3.26	.001
NoS*Task:Choose	2.29	0.22	4.35	2.17	.030
NoS*Task:Predict	9.05	6.97	11.12	8.56	<.001
NoS*Reward:NC	-0.31	-2.34	1.73	0.30	.767
Task:Choose*Reward:NC	4.32	1.06	7.58	2.60	.009
Task:Predict*Reward:NC	0.00	-3.27	3.26	0.00	.998
NoS*Task:Choose*Reward:NC	-1.51	-4.42	1.4	1.02	.308
NoS*Task:Predict*Reward:NC	-2.04	-4.95	0.86	1.38	.167
Simple Effects (Number of Successes by Condition)					
Observe; Contingent Bonus	0.47	-0.99	1.92	0.63	.530
Choose; Contingent Bonus	2.75	1.29	4.22	3.68	<.001
Predict; Contingent Bonus	9.51	8.03	10.99	12.62	<.001

Observe; Non-contingent Bonus	0.16	-1.27	1.58	0.22	.828
Choose; Non-contingent Bonus	0.94	-0.54	2.41	1.24	.213
Predict; Non-contingent Bonus	7.16	5.71	8.61	9.67	<.001
Contrasts					
Contingent Bonus					
Observe – Choose	-2.29	-5.21	0.63	2.17	.269
Observe – Predict	-9.05	-11.98	-6.11	8.56	<.001
Choose – Predict	-6.76	-9.70	-3.81	6.36	<.001
Non-contingent Bonus					
Observe – Choose	-0.78	-3.68	2.12	0.74	>.999
Observe – Predict	-7.00	-9.88	-4.12	6.75	<.001
Choose – Predict	-6.22	-9.15	-3.30	5.90	<.001
Task: Observe					
Contingent Bonus – NC Bonus	0.31	-2.57	3.19	0.30	>.999
Task: Choose					
Contingent Bonus – NC Bonus	1.82	-1.12	4.76	1.71	.778
Task: Predict					
Contingent Bonus – NC Bonus	2.35	-0.58	5.28	2.23	.234

Figure S2: ***Self-rated ability to predict future coin toss outcomes by number of experienced successes, Reward Contingency condition, and Task condition (Experiment 5).*** *For "Observe" and "Choose" conditions, success = heads outcome. For "Predict" condition, success = correct prediction. Error bars = 95% confidence intervals.*

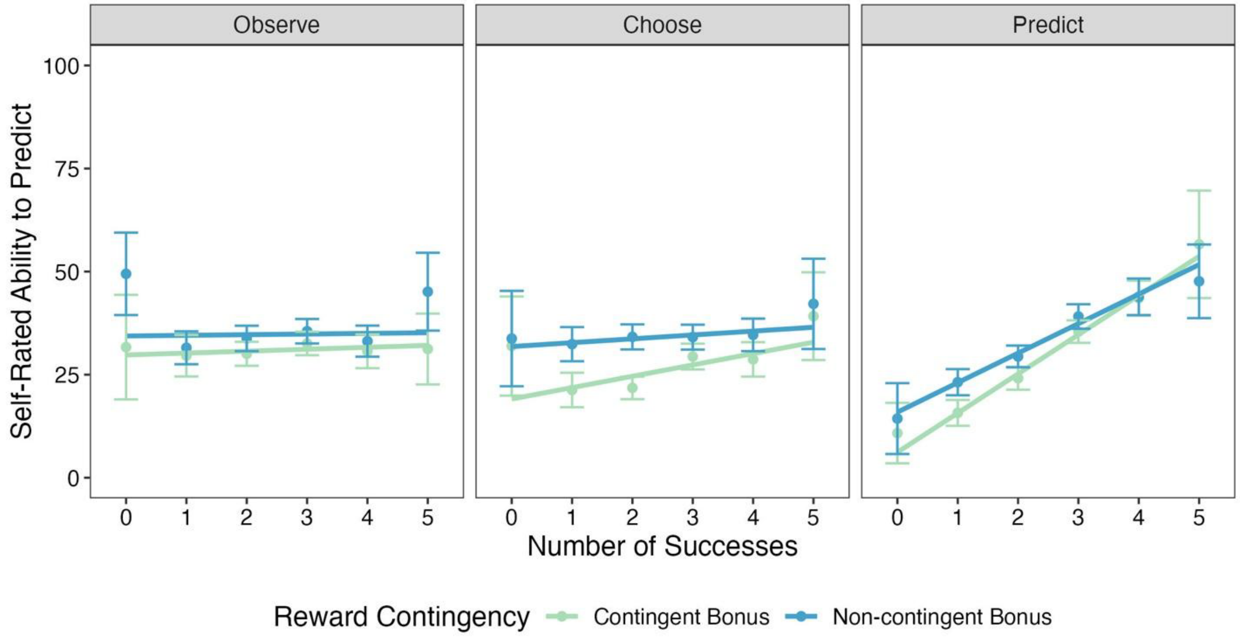

Expected Number of Correct Predictions (out of 20)

We included the expected number of correct predictions (out of 20) as an alternative measure of ability to predict future tosses that required participants to explicitly quantify their expectations about future prediction success. Significant positive effects of the number of successes on this measure were observed across all studies (*Table S4*). Experiment 4 did not reveal significant effects of *Reward Contingency* on this effect (*Table S5, Figure S3*), and Experiment 5 revealed that this effect was limited to participants in the "Predict" condition but not observed in the "Observe" or "Choose" conditions (presumably because the nature of their tasks was not to predict outcomes, see *Table S6, Figure S4*).

Table S4: ***Regression analyses for expected number of correct predictions out of 20 as a function of number of experienced successes.*** *For Experiments 2-4, these judgments came after 10 trials, with risk taking decisions being made after five trials. For Experiment 4, this result is averaged over the three levels of the Reward Contingency variable (No Bonus, Contingent Bonus, Non-contingent Bonus). For Experiment 5, this result is drawn from the subset of participants who experienced the Predict condition (n = 2010) and is averaged over the two levels of the Reward Contingency variable (Contingent Bonus, Non-contingent Bonus).*

Expected Number of Correct Predictions	*b*	95% CI LB	95% CI UB	*t*	*p*
Experiment 1					
Intercept	9.66	9.49	9.84	107.29	<.001
Number of Successes	1.20	1.04	1.36	14.77	<.001
Experiment 2					
Intercept	9.20	9.04	9.37	108.94	<.001
Number of Successes	0.81	0.70	0.91	15.14	<.001
Experiment 3					
Intercept	9.68	9.51	9.85	113.13	<.001
Number of Successes	0.67	0.57	0.78	12.33	<.001
Experiment 4					
Intercept	9.20	9.10	9.30	185.83	<.001
Number of Successes	0.69	0.63	0.75	22.02	<.001
Experiment 5					
Intercept	9.34	9.22	9.45	154.73	<.001
Number of Successes	0.99	0.89	1.10	18.29	<.001

Table S5: ***Regression analysis for number of expected correct predictions as a function of number of experienced successes and Reward Contingency condition (Experiment 4).*** *Reward Contingency conditions dummy-coded (reference condition: No Bonus).*

Experiment 4: Number of Expected Correct Predictions	*b*	95% CI LB	95% CI UB	*t*	*p*
(Intercept)	5.81	5.26	6.37	20.47	<.001
Number of Successes	0.67	0.56	0.77	12.28	<.001
Contingent Bonus	-0.13	-0.92	0.66	0.32	.745
Non-contingent Bonus	-0.14	-0.93	0.66	0.33	.739
Number of Successes*Contingent Bonus	0.02	-0.13	0.16	0.20	.840
Number of Successes*Non-contingent Bonus	0.05	-0.10	0.20	0.66	.508
Simple Effects (Number of Successes by Condition)					
No Bonus	0.67	0.56	0.77	12.28	<.001
Contingent Bonus	0.68	0.58	0.79	12.77	<.001
Non-contingent Bonus	0.72	0.61	0.82	13.07	<.001
Contrasts					
No Bonus – Contingent Bonus	-0.02	-0.20	0.17	0.20	>.999
No Bonus – Non-contingent Bonus	-0.05	-0.24	0.13	0.66	>.999
Contingent Bonus – Non-contingent Bonus	-0.04	-0.22	0.15	0.47	>.999

Figure S3: ***Expected number of correct predictions by number of experienced successes and Reward Contingency condition (Experiment 4).*** *Error bars = 95% confidence intervals.*

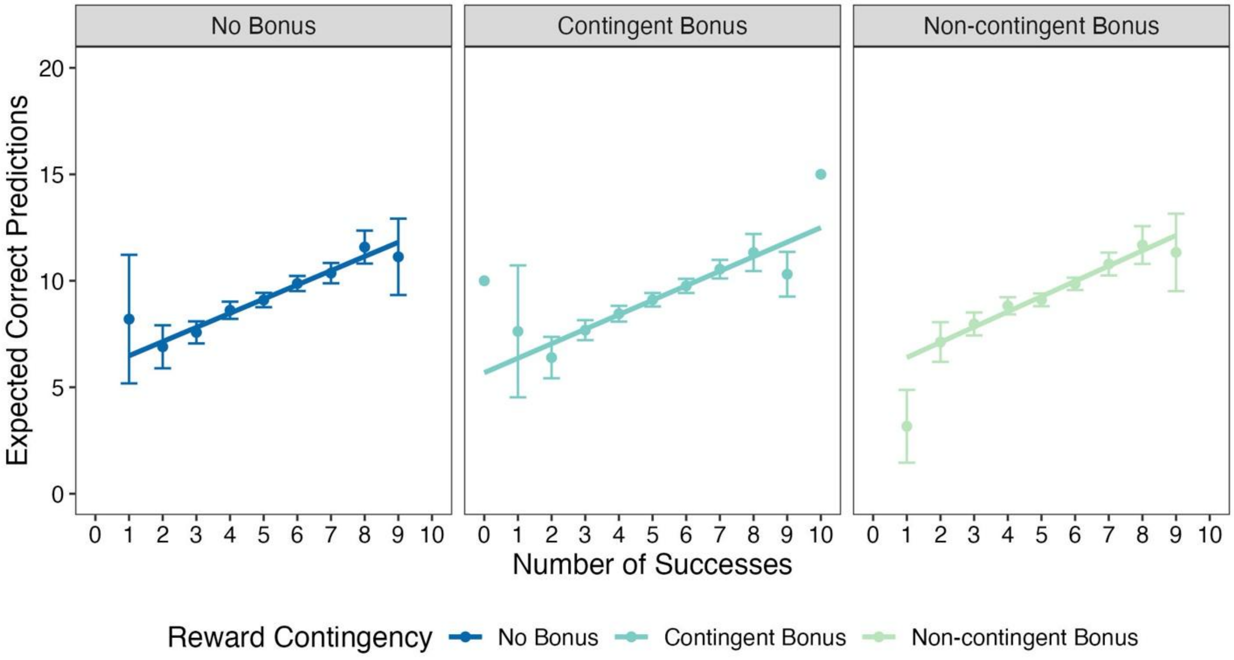

Table S6: ***Regression analysis for expected number of correct predictions out of 20 as a function of number of experienced successes, Reward Contingency condition, and Task condition (Experiment 5).*** *Reward Contingency conditions dummy-coded (reference condition: Contingent Bonus). Task conditions dummy-coded (reference condition: Observe). Contrasts are Bonferroni corrected for multiple comparisons.*

Experiment 5: Expected Number of Correct Predictions by Condition	*b*	95% CI LB	95% CI UB	*t*	*p*
(Intercept)	8.74	8.56	8.93	92.55	<.001
Number of Successes (NoS)	0.14	-0.02	0.31	1.68	.094
Task: Choose	0.24	-0.02	0.5	1.82	.069
Task: Predict	0.48	0.22	0.74	3.58	<.001
Reward: Non-contingent (NC)	0.65	0.38	0.91	4.83	<.001
NoS*Task:Choose	0.17	-0.07	0.40	1.42	.157
NoS*Task:Predict	0.98	0.74	1.21	8.12	<.001
NoS*Reward:NC	0.05	-0.19	0.28	0.39	.699
Task:Choose*Reward:NC	0.05	-0.32	0.42	0.25	.799
Task:Predict*Reward:NC	-0.38	-0.75	-0.01	2.02	.043
NoS*Task:Choose*Reward:NC	-0.30	-0.63	0.03	1.79	.073
NoS*Task:Predict*Reward:NC	-0.29	-0.62	0.04	1.70	.089
Simple Effects (Number of Successes by Condition)					
Observe; Contingent Bonus	0.14	-0.02	0.31	1.68	.094

Choose; Contingent Bonus	0.31	0.14	0.48	3.66	<.001
Predict; Contingent Bonus	1.12	0.95	1.28	13.04	<.001
Observe; Non-contingent Bonus	0.19	0.03	0.35	2.27	.023
Choose; Non-contingent Bonus	0.05	-0.11	0.22	0.63	.528
Predict; Non-contingent Bonus	0.88	0.71	1.04	10.44	<.001
Contrasts					
Contingent Bonus					
Observe – Choose	-0.17	-0.5	0.16	1.42	>.999
Observe – Predict	-0.98	-1.31	-0.64	8.12	<.001
Choose – Predict	-0.81	-1.14	-0.47	6.69	<.001
Non-contingent Bonus					
Observe – Choose	0.13	-0.20	0.46	1.12	>.999
Observe – Predict	-0.69	-1.02	-0.36	5.87	<.001
Choose – Predict	-0.82	-1.15	-0.49	6.86	<.001
Task: Observe					
Contingent Bonus – NC Bonus	-0.05	-0.37	0.28	0.39	>.999
Task: Choose					
Contingent Bonus – NC Bonus	0.26	-0.08	0.59	2.13	.298
Task: Predict					
Contingent Bonus – NC Bonus	0.24	-0.09	0.57	2.01	.403

Figure S4: ***Expected number of correct predictions as a function of number of experienced successes, Reward Contingency condition, and Task condition (Experiment 5).*** *For "Observe" and "Choose" conditions, success = heads outcome. For "Predict" condition, success = correct prediction. Error bars = 95% confidence intervals.*

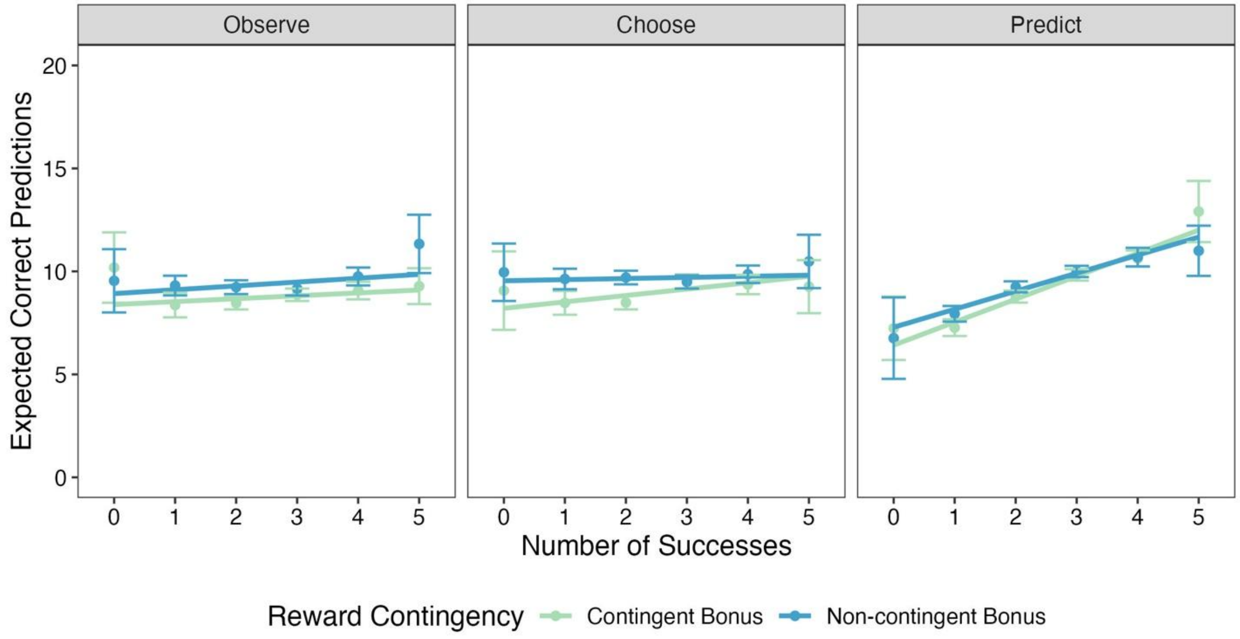

Risk-taking Intentions and Behavior

Risk tolerance was measured in three different ways across experiments (*Table S38*). Experiment 5 was conducted first, and included a self-reported willingness to bet on future predictions measured on a continuous scale, but not played out. This measure captured participants' hypothetical risk-taking intentions and was significantly affected by the number of successes (*Tables S7 and S9*). Experiment 1 was conducted next, and included a binary behavioral measure of risk taking (willingness to play again vs. take a sure amount of money). This measure was not sensitive to the number of successes (both linear and logit models reported in *Table S7*). There are two possible reasons that this was sample-dependent. Experimenters noted that many participants indicated that they found the coin flipping task quite fun and that the modest payment in the form of an Amazon gift card delivered by email was not their primary motivation for participating in the study. This suggests that the decision to play again may not have been entirely a function of the relative monetary value of the choices, but of other factors (e.g., enjoyment). Given this null result, we tested a more sensitive measure of risk behavior in Experiments 2, 3, and 4 that compelled participants to play again and to decide on a continuous scale how much risk they were willing to take on. This measure was consistently affected by the number of successes across experiments (*Table S7*). Experiment 4 found no effect of *Reward Contingency* on risk behavior (*Table S8*, *Figure S5*).

Table S7: ***Regression analyses for risk-taking behavior as a function of number of experienced successes.*** *For Experiment 1, participants either accepted or rejected a risky bet (binary choice). For Experiments 2-4, participants wagered based on their expected future rate of prediction accuracy. For Experiment 5, participants self-reported their willingness to bet on their predictions (continuous scale). For Experiment 4, this result is averaged across the three levels of the Reward Contingency variable (No Bonus, Contingent Bonus, Non-contingent Bonus). For Experiment 5, this result is drawn from the subset of participants who experienced the Predict condition (n = 2010) and is averaged across the two levels of the Reward Contingency variable (Contingent Bonus, Non-contingent Bonus).*

Risk	*b*	95% CI LB	95% CI UB	*t*	*p*
Experiment 1: Logit Model					
Intercept	0.44	0.31	0.57	6.78	<.001
Number of Successes	0.01	-0.10	0.13	0.21	.834
Experiment 1: Linear Model					
Intercept	0.61	0.58	0.64	39.32	<.001
Number of Successes	0.003	-0.02	0.03	0.21	.835
Experiment 2					
Intercept	2.02	1.96	2.09	62.75	<.001
Number of Successes	0.17	0.12	0.23	6.14	<.001
Experiment 3					
Intercept	2.37	2.31	2.42	82.67	<.001
Number of Successes	0.09	0.04	0.15	3.63	<.001
Experiment 4					

Intercept	2.06	2.02	2.10	110.28	<.001
Number of Successes	0.15	0.12	0.18	8.90	<.001
Experiment 5					
Intercept	26.97	25.39	28.55	33.44	<.001
Number of Successes	4.01	2.59	5.43	5.54	<.001

Table S8: ***Regression analysis for wagered number of correct predictions as a function of number of experienced successes and Reward Contingency condition (Experiment 4)****. Reward Contingency conditions dummy-coded (reference condition: No Bonus).*

Experiment 4: Risky Bet by Reward Contingency Condition	*b*	95% CI LB	95% CI UB	*t*	*p*
(Intercept)	2.04	1.98	2.11	63.19	<.001
Number of Successes	0.14	0.08	0.20	4.88	<.001
Contingent Bonus	-0.001	-0.09	0.09	0.03	.974
Non-contingent Bonus	0.05	-0.04	0.14	1.05	.293
Number of Successes*Contingent Bonus	-0.004	-0.08	0.07	0.12	.907
Number of Successes*Non-contingent Bonus	0.03	-0.05	0.11	0.76	.447
Simple Effects (Number of Successes by Condition)					
No Bonus	0.14	0.08	0.20	4.88	<.001
Contingent Bonus	0.13	0.08	0.19	4.68	<.001
Non-contingent Bonus	0.17	0.11	0.23	5.83	<.001
Contrasts					
No Bonus – Contingent Bonus	0.005	-0.09	0.10	0.12	>.999
No Bonus – Non-contingent Bonus	-0.03	-0.13	0.07	0.76	>.999
Contingent Bonus – Non-contingent Bonus	-0.04	-0.13	0.06	0.87	>.999

Figure S5: ***Wagered number of correct predictions by number of experienced successes and Reward Contingency condition (Experiment 4)****. Error bars = 95% confidence intervals.*

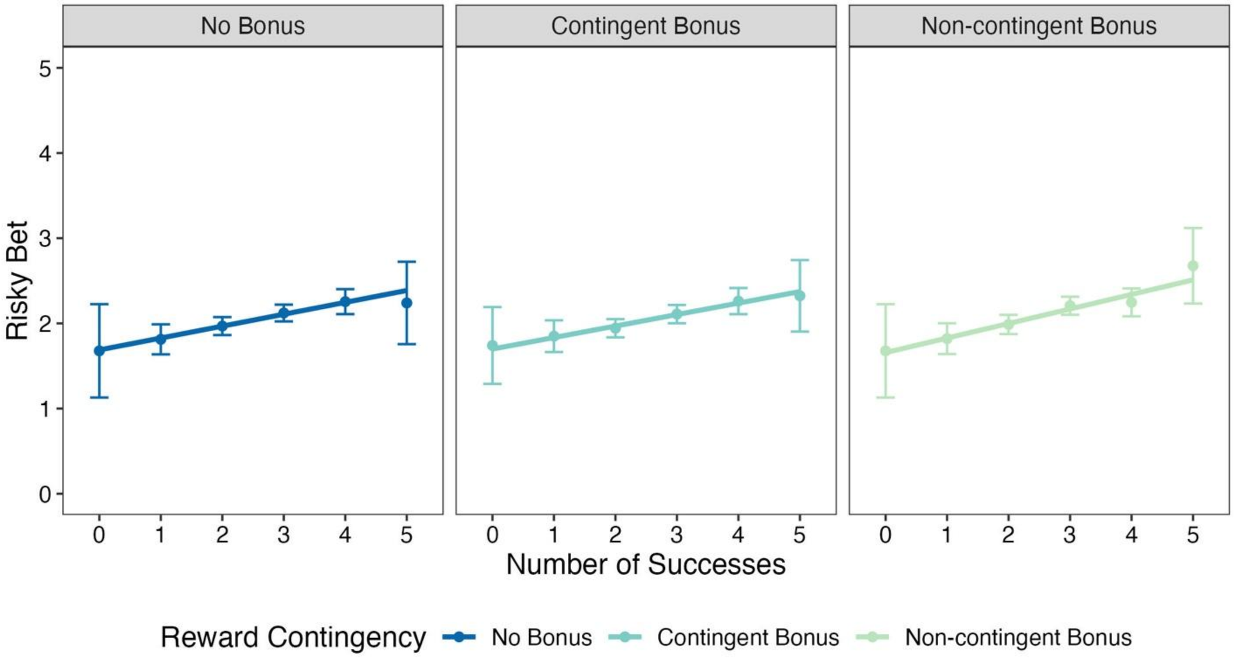

Table S9: ***Regression analysis for self-reported willingness to bet on predictions as a function of number of experienced successes, Reward Contingency condition, and Task condition (Experiment 5).*** *Reward Contingency conditions dummy-coded (reference condition: Contingent Bonus). Task conditions dummy-coded (reference condition: Observe). Contrasts are Bonferroni corrected for multiple comparisons.*

Experiment 5: Self-reported willingness to bet on Predictions	*b*	95% CI LB	95% CI UB	*t*	*p*
(Intercept)	26.39	24.15	28.62	23.15	<.001
Number of Successes (NoS)	-0.81	-2.80	1.18	0.80	.425
Task: Choose	0.59	-2.57	3.75	0.37	.714
Task: Predict	1.33	-1.82	4.49	0.83	.408
Reward: Non-contingent (NC)	0.38	-2.78	3.54	0.24	.814
NoS*Task:Choose	1.08	-1.75	3.91	0.75	.454
NoS*Task:Predict	6.33	3.49	9.17	4.37	<.001
NoS*Reward:NC	-0.20	-2.98	2.59	0.14	.890
Task:Choose*Reward:NC	3.08	-1.38	7.55	1.35	.176
Task:Predict*Reward:NC	-1.78	-6.25	2.68	0.78	.434
NoS*Task:Choose*Reward:NC	0.24	-3.75	4.23	0.12	.906
NoS*Task:Predict*Reward:NC	-2.79	-6.77	1.18	1.38	.169
Simple Effects (Number of Successes by Condition)					
Observe; Contingent Bonus	-0.81	-2.8	1.18	0.80	.425

Choose; Contingent Bonus	0.27	-1.74	2.28	0.26	.793
Predict; Contingent Bonus	5.52	3.49	7.54	5.35	<.001
Observe; Non-contingent Bonus	-1.01	-2.96	0.95	1.01	.312
Choose; Non-contingent Bonus	0.31	-1.7	2.33	0.30	.761
Predict; Non-contingent Bonus	2.53	0.54	4.52	2.50	.013
Contrasts					
Contingent Bonus					
Observe – Choose	-1.08	-5.08	2.92	0.75	>.999
Observe – Predict	-6.33	-10.34	-2.31	4.37	<.001
Choose – Predict	-5.25	-9.29	-1.21	3.61	0.003
Non-contingent Bonus					
Observe – Choose	-1.32	-5.29	2.65	0.92	>.999
Observe – Predict	-3.54	-7.48	0.40	2.49	.116
Choose – Predict	-2.22	-6.22	1.79	1.53	>.999
Task: Observe					
Contingent Bonus – NC Bonus	0.20	-3.75	4.14	0.14	>.999
Task: Choose					
Contingent Bonus – NC Bonus	-0.04	-4.08	3.99	0.03	>.999
Task: Predict					
Contingent Bonus – NC Bonus	2.99	-1.02	7.00	2.07	.350

Figure S6: ***Self-reported willingness to bet on predictions as a function of number of experienced successes, Reward Contingency condition, and Task condition (Experiment 5).*** *For "Observe" and "Choose" conditions, success = heads outcome. For "Predict" condition, success = correct prediction. Error bars = 95% confidence intervals.*

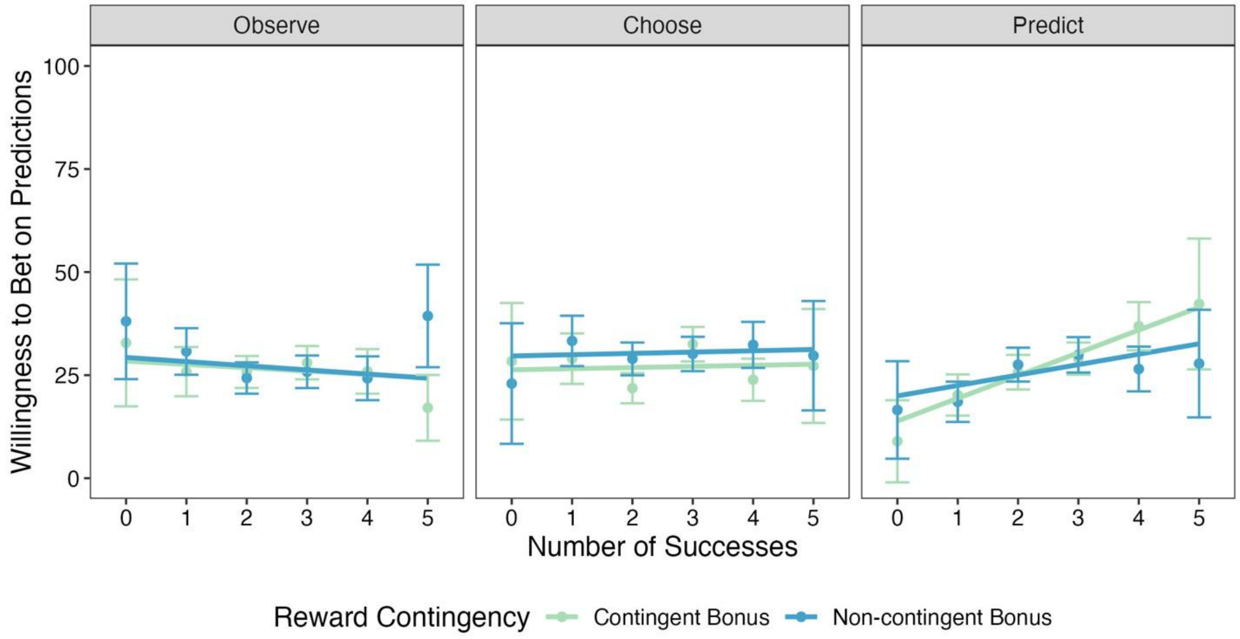

Expected Number of Heads (out of 20)

In three experiments (Experiments 2,4,5), we measured the expected number of "heads" outcomes out of 20 as a function of the number of successes. Interestingly, participants who experienced more successful predictions expected a greater number of "heads" outcomes across all three studies (*Table S10*). This may seem surprising, but is consistent with other evidence suggesting that people tend to choose "heads" more often than "tails" when predicting coin tosses (our own data confirms this) and even do so when mentally simulating a series of coin tosses (*58*). Because of this "heads bias," both successful and unsuccessful participants would have been more likely to have forecasted "heads" outcomes, and may have expected more of them in the future if those predictions were successful and less of them in the future if those predictions were unsuccessful. Given our evidence that prediction success affected how many heads outcomes participants expected to occur on future trials, we tested whether this effect was smaller or larger than the effect on the expected number of correct predictions by computing a difference score (expected correct predictions minus expected heads) and regressing this variable on the number of successful predictions. Across the three experiments in which both variables were measured (Experiments, 2, 4, and 5), *b* values were always significantly positive, meaning that the effect of number of successes on expected number of correct predictions was always greater than the effect of number of successes on expected number of heads ($b_{Exp2} = 0.52$, $t_{Exp2}(1{,}001) = 8.36$, $p_{Exp2} < .001$; $b_{Exp4} = 0.42$, $t_{Exp4}(2{,}998) = 11.89$, $p_{Exp4} < .001$; $b_{Exp5} = 0.58$, $t_{Exp5}(2{,}010) = 9.37$, $p_{Exp5} < .001$). For Experiment 4, this result is averaged over the three levels of the Reward Contingency variable (No Bonus, Contingent Bonus, Non-contingent Bonus). For Experiment 5, this result is drawn from the subset of participants who experienced the Predict condition (n = 2010) and is averaged over the two levels of the Reward Contingency variable (Contingent Bonus, Non-contingent Bonus).

Experiment 4 and Experiment 5 did not reveal significant effects of *Reward Contingency* on this effect (*Tables S11 and S12*). Experiment 5 revealed this effect was significantly larger for participants in the "Observe" and "Choose" conditions relative to those in the "Predict" condition (*Table S12*). Presumably this large difference resulted from the difference in what constituted "success" between those conditions ("heads" outcomes for the "Observe" and "Choose" conditions, correct predictions for the "Predict" condition).

Table S10: ***Regression analyses for expected number of heads outcomes out of 20 as a function of number of experienced successes.*** *For Experiments 2 and 4, these judgments came after 10 trials, with risk taking decisions being made after five trials. For Experiment 4, this result is averaged over the three levels of the Reward Contingency variable (No Bonus, Contingent Bonus, Non-contingent Bonus). For Experiment 5, this result is drawn from the subset of participants who experienced the Predict condition (n = 2010) and is averaged over the two levels of the Reward Contingency variable (Contingent Bonus, Non-contingent Bonus).*

Expected Number of Heads	*b*	95% CI LB	95% CI UB	*t*	*p*
Experiment 2					
Intercept	10.18	10.03	10.34	129.32	<.001
Number of Successes	0.29	0.19	0.38	5.77	<.001
Experiment 4					
Intercept	10.20	10.11	10.28	233.93	<.001

Number of Successes	0.26	0.21	0.32	9.58	<.001
Experiment 5					
Intercept	10.18	10.09	10.28	209.00	<.001
Number of Successes	0.39	0.31	0.48	8.94	<.001

Table S11: ***Regression analysis for number of expected heads outcomes as a function of number of experienced successes and Reward Contingency condition (Experiment 4).*** *Reward Contingency conditions dummy-coded (reference condition: No Bonus).*

Experiment 4: Number of Expected Heads	*b*	95% CI LB	95% CI UB	*t*	*p*
(Intercept)	8.75	8.26	9.24	35.03	<.001
Number of Successes	0.28	0.18	0.37	5.79	<.001
Contingent Bonus	0.11	-0.59	0.80	0.30	.762
Non-contingent Bonus	0.24	-0.46	0.94	0.68	.498
Number of Successes*Contingent Bonus	-0.03	-0.16	0.10	0.43	.667
Number of Successes*Non-contingent Bonus	-0.01	-0.14	0.12	0.16	.876
Simple Effects (Number of Successes by Condition)					
No Bonus	0.28	0.18	0.37	5.79	<.001
Contingent Bonus	0.25	0.16	0.34	5.27	<.001
Non-contingent Bonus	0.27	0.17	0.36	5.50	<.001
Contrasts					
No Bonus – Contingent Bonus	0.03	-0.13	0.19	0.43	>.999
No Bonus – Non-contingent Bonus	0.01	-0.15	0.17	0.16	>.999
Contingent Bonus – Non-contingent Bonus	-0.02	-0.18	0.14	-0.27	>.999

Table S12: ***Regression analysis for expected number of heads outcomes out of 20 as a function of number of experienced successes, Reward Contingency condition, and Task condition (Experiment 5).*** *Reward Contingency conditions dummy-coded (reference condition: Contingent Bonus). Task conditions dummy-coded (reference condition: Observe). Contrasts are Bonferroni corrected for multiple comparisons.*

Experiment 5: Number of Expected Heads by Condition	*b*	95% CI LB	95% CI UB	*t*	*p*
(Intercept)	9.63	9.49	9.77	135.92	<.001

Number of Successes (NoS)	0.87	0.74	0.99	13.77	<.001
Task: Choose	0.25	0.06	0.45	2.54	.011
Task: Predict	0.59	0.40	0.79	5.92	<.001
Reward: Non-contingent (NC)	0.72	0.53	0.92	7.24	<.001
NoS*Task:Choose	0.01	-0.16	0.19	0.13	.893
NoS*Task:Predict	-0.47	-0.65	-0.30	5.23	<.001
NoS*Reward:NC	0.02	-0.16	0.19	0.17	.862
Task:Choose*Reward:NC	-0.25	-0.53	0.02	1.80	.073
Task:Predict*Reward:NC	-0.78	-1.06	-0.50	5.51	<.001
NoS*Task:Choose*Reward:NC	-0.02	-0.27	0.22	0.19	.848
NoS*Task:Predict*Reward:NC	-0.02	-0.27	0.23	0.15	.879
Simple Effects (Number of Successes by Condition)					
Observe; Contingent Bonus	0.87	0.74	0.99	13.77	<.001
Choose; Contingent Bonus	0.88	0.75	1.00	13.77	<.001
Predict; Contingent Bonus	0.39	0.27	0.52	6.06	<.001
Observe; Non-contingent Bonus	0.88	0.76	1.00	14.27	<.001
Choose; Non-contingent Bonus	0.87	0.74	1.00	13.52	<.001
Predict; Non-contingent Bonus	0.39	0.27	0.51	6.14	<.001
Contrasts					
Contingent Bonus					
Observe – Choose	-0.01	-0.26	0.24	0.13	>.999
Observe – Predict	0.47	0.22	0.72	5.23	<.001
Choose – Predict	0.48	0.23	0.74	5.32	<.001
Non-contingent Bonus					
Observe – Choose	0.01	-0.24	0.26	0.14	>.999
Observe – Predict	0.49	0.25	0.74	5.55	<.001
Choose – Predict	0.48	0.23	0.73	5.31	<.001
Task: Observe					
Contingent Bonus – NC Bonus	-0.02	-0.26	0.23	0.17	>.999
Task: Choose					
Contingent Bonus – NC Bonus	0.01	-0.24	0.26	0.10	>.999
Task: Predict					
Contingent Bonus – NC Bonus	0.004	-0.25	0.26	0.04	>.999

Willingness to Bet on Heads

In Experiment 5 we measured participants' self-reported willingness to bet on heads outcomes. This measure was sensitive to the number of successes in all three *Contingent Bonus* conditions and in the *Choose* condition when rewards were not contingent on trial outcomes, although there were no significant differences between conditions (*Table S13*).

Table S13: ***Regression analysis for willingness to bet on heads as a function of number of experienced successes, Reward Contingency condition, and Task condition (Experiment 5).*** *Reward Contingency conditions dummy-coded (reference condition: Contingent Bonus). Task conditions dummy-coded (reference condition: Observe). Contrasts are Bonferroni corrected for multiple comparisons.*

Experiment 5: Willingness to bet on heads by Condition	*b*	95% CI LB	95% CI UB	*t*	*p*
(Intercept)	35.78	33.39	38.17	29.35	<.001
Number of Successes (NoS)	2.27	0.14	4.40	2.09	.037
Task: Choose	-3.57	-6.95	-0.20	2.07	.038
Task: Predict	-7.13	-10.51	-3.76	4.14	<.001
Reward: Non-contingent (NC)	-3.57	-6.95	-0.19	2.07	.038
NoS*Task:Choose	1.16	-1.87	4.18	0.75	.454
NoS*Task:Predict	1.70	-1.34	4.73	1.10	.273
NoS*Reward:NC	-1.61	-4.59	1.37	1.06	.289
Task:Choose*Reward:NC	5.22	0.44	10.00	2.14	.032
Task:Predict*Reward:NC	1.78	-3.00	6.56	0.73	.466
NoS*Task:Choose*Reward:NC	1.84	-2.42	6.11	0.85	.396
NoS*Task:Predict*Reward:NC	-1.78	-6.03	2.48	0.82	.413
Simple Effects (Number of Successes by Condition)					
Observe; Contingent Bonus	2.27	0.14	4.40	2.09	.037
Choose; Contingent Bonus	3.42	1.27	5.58	3.12	.002
Predict; Contingent Bonus	3.97	1.80	6.13	3.59	<.001
Observe; Non-contingent Bonus	0.66	-1.43	2.74	0.62	.538
Choose; Non-contingent Bonus	3.66	1.50	5.81	3.32	.001
Predict; Non-contingent Bonus	0.58	-1.55	2.70	0.53	.594
Contrasts					
Contingent Bonus					
Observe – Choose	-1.16	-5.44	3.13	0.75	>.999
Observe – Predict	-1.70	-5.99	2.60	1.10	>.999
Choose – Predict	-0.54	-4.86	3.78	0.35	>.999
Non-contingent Bonus					
Observe – Choose	-3.00	-7.25	1.25	1.96	.452
Observe – Predict	0.08	-4.14	4.29	0.05	>.999
Choose – Predict	3.08	-1.21	7.37	1.99	.418
Task: Observe					
Contingent Bonus – NC Bonus	1.61	-2.61	5.83	1.06	>.999
Task: Choose					
Contingent Bonus – NC Bonus	-0.23	-4.54	4.08	0.15	>.999
Task: Predict					
Contingent Bonus – NC Bonus	3.39	-0.91	7.68	2.19	.258

Luck and Skill Attributions

We measured luck and skill attributions both as a tradeoff (Experiments 1, 5) and individually (Experiments 2,3,4). When measured as a tradeoff, participants in general attributed their outcomes more to luck than to skill, but skill attributions increased as a function of the number of successful predictions (*Table S14, Figure S7*). When measured individually, participants rated their outcomes as both more attributable to luck *and* more attributable to skill (although not all effects reached statistical significance across all studies, see *Table S14, Figure S7*). We suspect that for people who believe both luck and skill affect task performance, success may lead them to believe that they are both luckier and more skillful. Experiment 4 found no significant differences in *Reward Contingency* condition on luck or skill attributions. Simple effects of the number of successes on luck and skill attributions were all significantly positive except for the "Non-contingent Bonus" condition for luck attributions (*Tables S15 and S16*).

Because one cannot be "lucky" or "skilled" when simply observing coin tosses or choosing between coins to toss without stakes, we measured luck/skill attributions in Experiment 5 only for participants experiencing the "Contingent Bonus" in the "Observe" and "Choose" conditions; and both *Reward Contingency* conditions for participants who experienced the "Predict" *Task* condition (wordings were modified to reflect experimental conditions experienced by the participants, see *Table S31*). A linear model testing the effect of *Reward Contingency* on luck/skill attributions limited to the participants who experienced the "Predict" condition found no effect of *Reward Contingency* (*Table S17*). Another linear model testing the effect of *Task* on luck/skill attributions limited to the participants in the "Contingent Bonus" condition found that participants in the "Predict" condition attributed their outcomes to skill rather than luck to a significantly greater extent than those in the "Choose" and the "Observe" conditions (*Table S18, Figure S8*). Simple effects of number of successes on increasing attribution of outcomes to skill were significantly positive in the "Predict" condition and also in the "Choose" condition (*Table S18, Figure S8*)

Table S14: ***Regression analyses for luck and skill attributions as a function of number of experienced successes.*** *Experiments 1 and 5 measured these attributions as a tradeoff (0) – Totally Luck; (100) – Totally Skill). Experiments 2, 3, and 4 measured them separately. For Experiment 4, this result is averaged over the three levels of the Reward Contingency variable (No Bonus, Contingent Bonus, Non-contingent Bonus). For Experiment 5, this result is drawn from the subset of participants who experienced the Predict condition (n = 2010) and is averaged over the two levels of the Reward Contingency variable (Contingent Bonus, Non-contingent Bonus).*

Luck/Skill Attributions	*b*	95% CI LB	95% CI UB	t	*p*
Luck-Skill Tradeoff					
Experiment 1					
(Intercept)	23.16	21.74	24.58	31.99	<.001
Number of Successes	2.17	0.90	3.44	3.36	<.001
Experiment 5					
(Intercept)	15.46	14.60	16.32	35.07	<.001
Number of Successes	3.00	2.23	3.78	7.58	<.001
Luck					

Experiment 2					
(Intercept)	65.21	63.15	67.27	62.19	<.001
Number of Successes	0.60	-0.70	1.90	0.91	.364
Experiment 3					
(Intercept)	55.93	53.66	58.19	48.50	<.001
Number of Successes	1.83	0.38	3.27	2.48	.013
Experiment 4					
(Intercept)	66.01	64.86	67.17	112.16	<.001
Number of Successes	1.21	0.48	1.94	3.27	.001
Skill					
Experiment 2					
(Intercept)	20.57	19.05	22.10	26.42	<.001
Number of Successes	2.55	1.59	3.51	5.20	<.001
Experiment 3					
(Intercept)	20.62	19.04	22.20	25.58	<.001
Number of Successes	0.84	-0.17	1.85	1.62	.105
Experiment 4					
(Intercept)	22.75	21.86	23.63	50.22	<.001
Number of Successes	3.03	2.47	3.59	10.60	<.001

Figure S7: ***Luck and skill attributions as a function of number of experienced successes.*** *Experiments 1 and 5 measured these attributions as a tradeoff (0) – Totally Luck; (100) – Totally Skill). Experiments 2, 3, and 4 measured them separately. For Experiment 4, this result is averaged over the three levels of the Reward Contingency variable (No Bonus, Contingent Bonus, Non-contingent Bonus). For Experiment 5, this result is drawn from the subset of participants who experienced the Predict condition (n = 2010) and is averaged over the two levels of the Reward Contingency variable (Contingent Bonus, Non-contingent Bonus). Error bars = 95% CIs.*

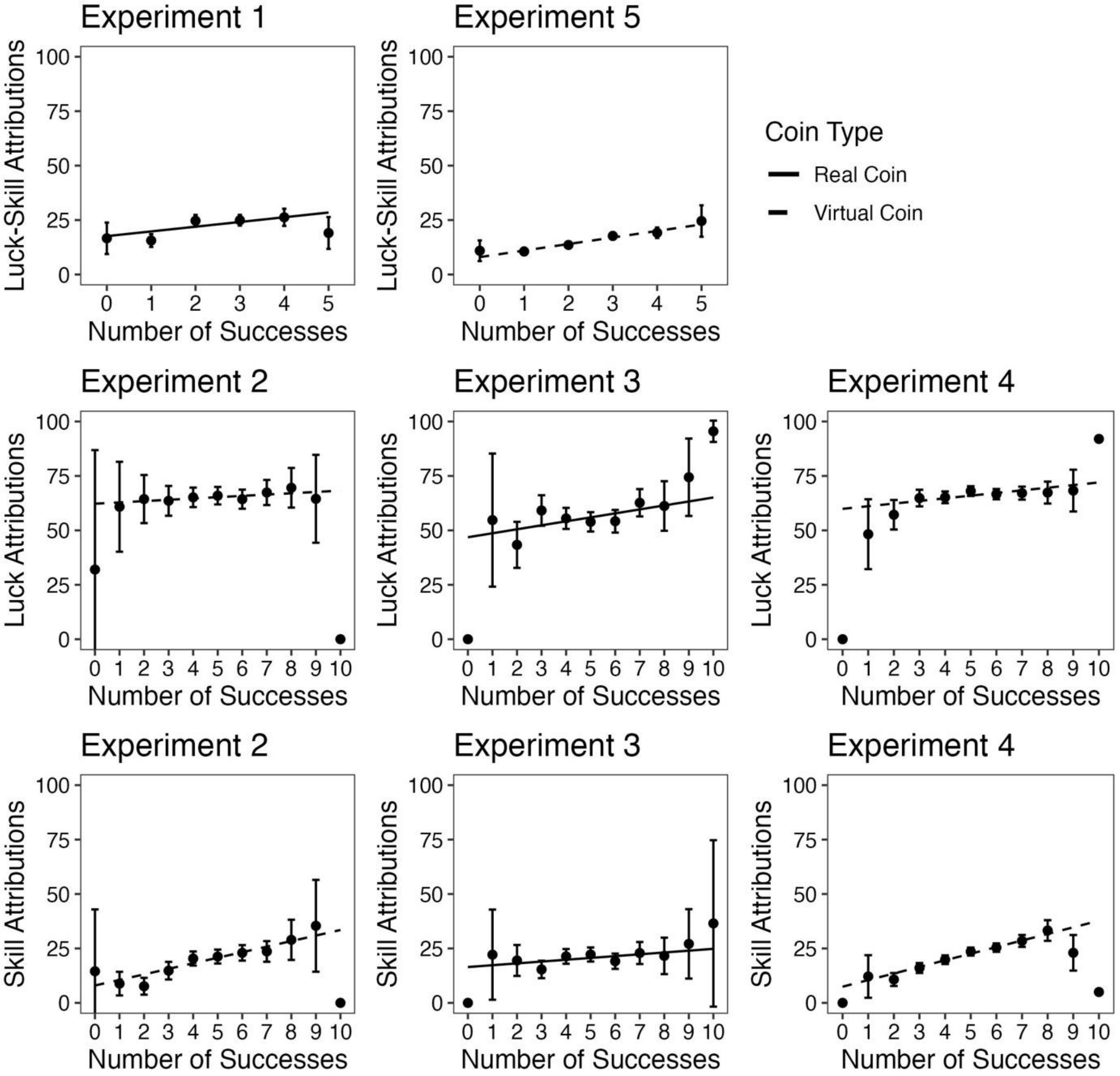

Table S15: ***Regression analysis for luck attributions as a function of number of experienced successes and Reward Contingency condition (Experiment 4).*** *Reward Contingency conditions dummy-coded (reference condition: No Bonus). Contrasts are Bonferroni corrected for multiple comparisons.*

Experiment 4: Luck Attributions by Reward Contingency Condition	*b*	95% CI LB	95% CI UB	*t*	*p*
(Intercept)	56.86	50.25	63.48	16.85	<.001
Number of Successes	1.64	0.37	2.90	2.54	.011
Contingent Bonus	1.54	-7.83	10.91	0.32	.748
Non-contingent Bonus	7.86	-1.62	17.35	1.63	.104
Number of Successes*Contingent Bonus	-0.14	-1.91	1.63	0.16	.876
Number of Successes*Non-contingent Bonus	-1.19	-2.99	0.61	1.30	.195
Simple Effects (Number of Successes by Condition)					
No Bonus	1.64	0.37	2.90	2.54	.011
Contingent Bonus	1.49	0.25	2.74	2.36	.019
Non-contingent Bonus	0.45	-0.83	1.73	0.69	.492
Contrasts					
No Bonus – Contingent Bonus	0.14	-2.02	2.31	0.16	>.999
No Bonus – Non-contingent Bonus	1.19	-1.01	3.38	1.30	.585
Contingent Bonus – Non-contingent Bonus	1.05	-1.13	3.23	1.15	.751

Table S16: ***Regression analysis for skill attributions as a function of number of experienced successes and Reward Contingency condition (Experiment 4).*** *Reward Contingency conditions dummy-coded (reference condition: No Bonus). Contrasts are Bonferroni corrected for multiple comparisons.*

Experiment 4: Skill Attributions by Reward Contingency Condition	*b*	*95% CI LB*	*95% CI UB*	*t*	*p*
(Intercept)	6.68	1.59	11.77	2.57	.010
Number of Successes	3.31	2.34	4.28	6.68	<.001
Contingent Bonus	0.91	-6.31	8.12	0.25	.805
Non-contingent Bonus	1.23	-6.07	8.53	0.33	.742

Number of Successes*Contingent Bonus	-0.49	-1.85	0.88	0.70	.483
Number of Successes*Non-contingent Bonus	-0.33	-1.71	1.06	0.46	.642
Simple Effects (Number of Successes by Condition)					
No Bonus	3.31	2.34	4.28	6.68	<.001
Contingent Bonus	2.82	1.87	3.78	5.78	<.001
Non-contingent Bonus	2.98	2.00	3.97	5.95	<.001
Contrasts					
No Bonus – Contingent Bonus	0.49	-1.18	2.16	0.7	>.999
No Bonus – Non-contingent Bonus	0.33	-1.36	2.02	0.46	>.999
Contingent Bonus – Non-contingent Bonus	-0.16	-1.84	1.52	0.23	>.999

Table S17: ***Luck/skill attributions as a function of Reward Contingency condition (Contingent Bonus, Non-contingent Bonus) (Experiment 5).*** *Reward Contingency conditions dummy-coded (reference condition: Contingent Bonus). Analysis is limited to the subset of participants who experienced the Predict condition (n = 2010).*

Experiment 5: Luck/Skill Attributions by Reward Contingency	*b*	95% CI LB	95% CI UB	*t*	*p*
(Intercept)	14.80	13.58	16.0 2	23.80	<.001
Number of Successes	3.42	2.32	4.53	6.06	<.001
Non-contingent Bonus	1.44	-0.29	3.17	1.64	.102
Number of Successes*Non-contingent Bonus	-0.81	-2.36	0.74	1.02	.306

Table S18: ***Luck/skill attributions as a function of Task condition (Observe, Choose, Predict) (Study 5).*** *Task conditions dummy-coded (reference condition: Observe). Analysis is limited to the subset of participants who experienced the Contingent Bonus condition (n = 3023). Contrasts include Bonferroni-corrected p-values.*

Experiment 5: Luck/Skill Attribution by Task	*b*	95% CI LB	95% CI UB	*t*	*p*
(Intercept)	9.94	8.82	11.07	17.33	<.001
Number of Successes	-0.002	-1.004	1.00	0.004	.997
Task: Choose	2.74	1.15	4.33	3.38	.001
Task: Predict	4.85	3.27	6.44	5.99	<.001
NoS*Task:Choose	1.06	-0.36	2.49	1.46	.144

NoS*Task:Predict	3.43	2.00	4.86	4.70	<.001
Simple Effects (Number of Successes by Condition)					
Observe	-0.002	-1.004	1.00	0.004	.997
Choose	1.06	0.05	2.07	2.05	.040
Predict	3.42	2.40	4.44	6.59	<.001
Contrasts					
Observe – Choose	-1.06	-2.80	0.67	1.46	.431
Observe - Predict	-3.43	-5.17	-1.68	4.70	<.001
Choose – Predict	-2.36	-4.12	-0.61	3.23	.004

Figure S8: ***Luck-Skill Attributions as a function of number of experienced successes, Reward Contingency condition, and Task condition (Experiment 5).*** *For "Observe" and "Choose" conditions, success = heads outcome. For "Predict" condition, success = correct prediction. For "Non-contingent Bonus" "Observe" and "Choose" conditions, no data were collected because a "heads" outcome is neither lucky nor unlucky without a stated win condition. Error bars = 95% confidence intervals.*

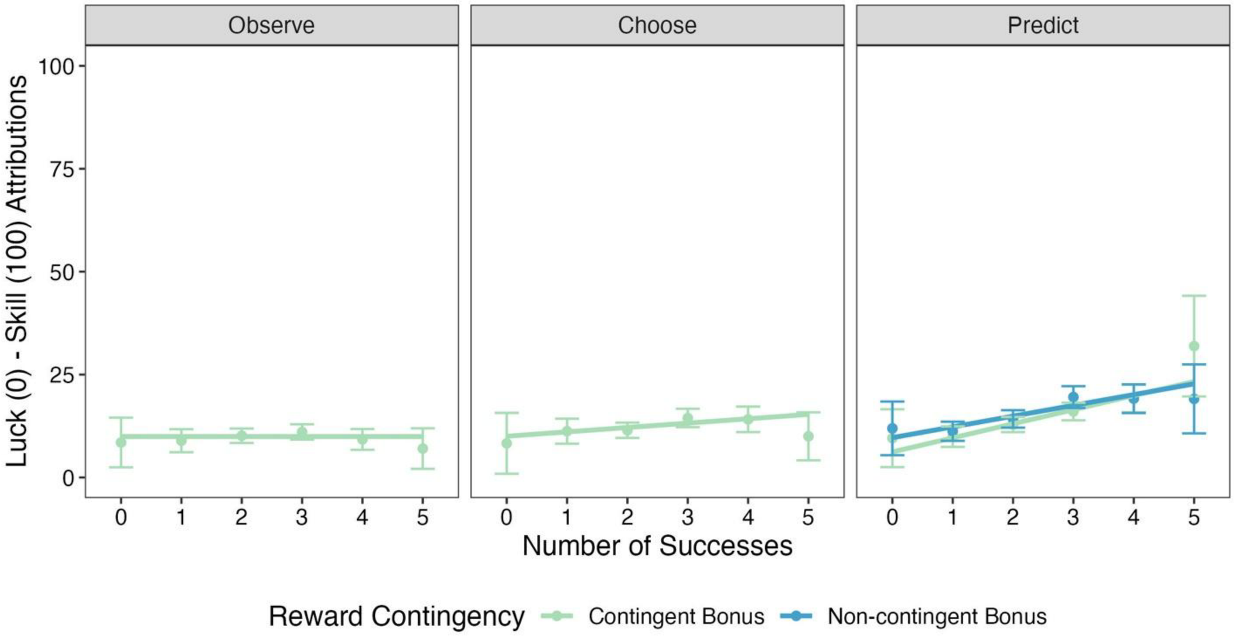

Confidence in Advice

In Experiment 4, participants wrote out advice on how to be successful in the task (these open-ended responses were not analyzed) and then rated their confidence that someone else following that advice would also be successful (*Table S37*). We found no differences in the effect of the number of successes on that confidence by *Reward Contingency* condition, but did

observe positive simple effects of the number of successes across all three conditions (*Table S19, Figure S9*). The more successful participants were in the prediction task, the more confident they were that someone following their advice would also be successful.

Table S19: ***Regression analysis for confidence in task advice as a function of number of experienced successes and Reward Contingency condition (Experiment 4).*** *Reward Contingency conditions dummy-coded (reference condition: No Bonus). Contrasts are Bonferroni corrected for multiple comparisons.*

Experiment 4: Confidence in Advice by Reward Contingency Condition	*b*	95% CI LB	95% CI UB	*t*	*p*
(Intercept)	38.1	32.94	43.26	14.48	<.001
Number of Successes	3.30	2.31	4.28	6.56	<.001
Contingent Bonus	-3.35	-10.66	3.96	-0.90	.368
Non-contingent Bonus	-0.46	-7.86	6.93	-0.12	.903
Number of Successes*Contingent Bonus	0.26	-1.12	1.64	0.37	.714
Number of Successes*Non-contingent Bonus	-0.16	-1.56	1.24	-0.22	.826
Simple Effects (Number of Successes by Condition)					
No Bonus	3.30	2.31	4.28	6.56	<.001
Contingent Bonus	3.56	2.59	4.53	7.19	<.001
Non-contingent Bonus	3.14	2.14	4.14	6.17	<.001
Contrasts					
No Bonus – Contingent Bonus	-0.26	-1.95	1.43	-0.37	>.999
No Bonus – Non-contingent Bonus	0.16	-1.56	1.87	0.22	>.999
Contingent Bonus – Non-contingent Bonus	0.42	-1.28	2.12	0.59	>.999

Figure S9: ***Confidence in task advice by number of experienced successes and Reward Contingency condition (Experiment 4).*** *Error bars = 95% confidence intervals.*

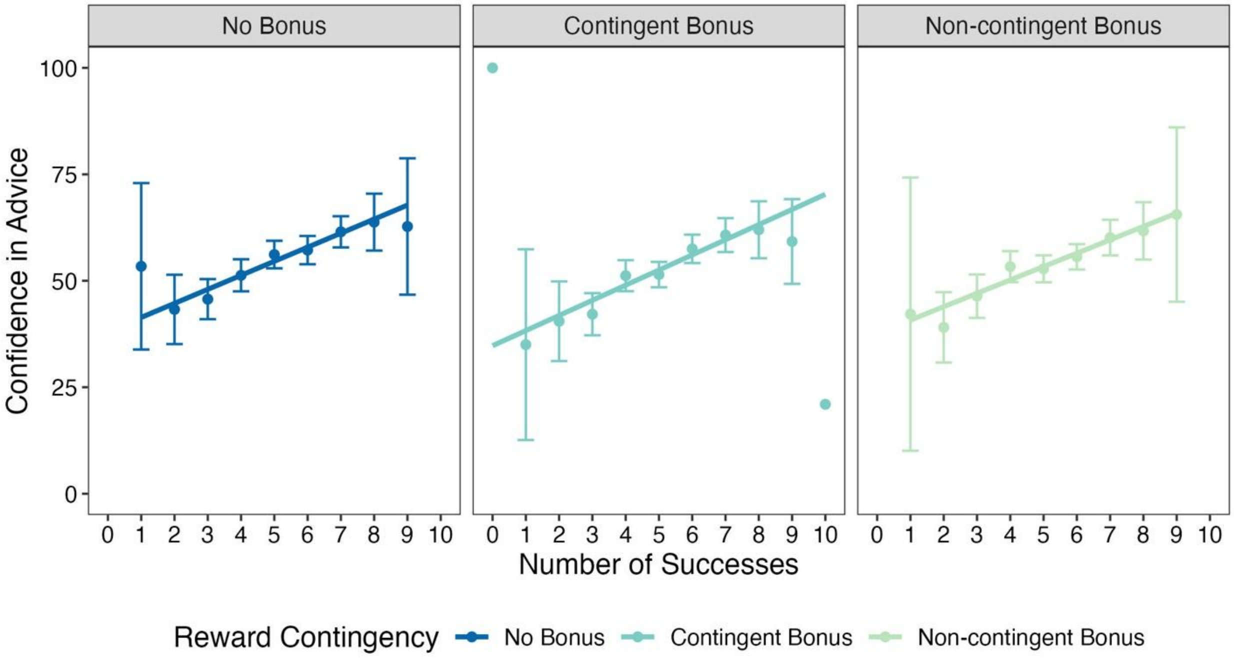

Random-Determined Process Beliefs

We measured participants' beliefs about whether the process producing the coin toss outcomes was "random" or "determined" on a continuous scale (*Table S37*). Simple linear regressions revealed that for participants tossing virtual coins (Experiments 2,4,5), prediction success was associated with the belief that the coin tossing mechanism was more random. However, inspection of the data at a descriptive level (e.g., *Figure S10, main text Figure 6*) suggested that a quadratic model would better capture participants' pattern of responses. Quadratic models predicting beliefs about the coin toss outcome generating process from number of successes revealed significant positive quadratic terms for experiments using a virtual coin, suggesting that participants who experienced rarer patterns of outcomes rated the process as less random (and more determined) than participants who experienced more common patterns of outcomes (*Table S20*). Additionally, the linear term in these models was significantly negative, suggesting that outcome rarity affects randomness judgments in part as a function of outcome valence. That is, it takes less rare negative outcomes to induce people to suspect that outcomes are not randomly determined relative to equally rare positive outcomes.

Experiment 4 did not reveal any differences in the linear effect of number of successes by *Reward Contingency* condition (*Table S21*). Experiment 5 did not reveal any differences in the linear effect of number of successes by *Task* condition, but revealed that the linear effect of number of successes on beliefs that outcomes were determined by random processes in the "Observe" and "Predict" conditions was significantly greater for participants in the "Non-contingent Bonus" condition relative to those in the "Contingent Bonus" condition (*Table S22*).

Table S20: ***Regression analyses for beliefs about the coin toss outcomes resulting from a Random (0) – Determined (100) process as a function of number of experienced successes.*** *For Experiment 4, this result is averaged over the three levels of the Reward Contingency*

variable (No Bonus, Contingent Bonus, Non-contingent Bonus). For Experiment 5, this result is drawn from the subset of participants who experienced the Predict condition (n = 2010) and is averaged over the two levels of the Reward Contingency variable (Contingent Bonus, Non-contingent Bonus).

Random vs Determined Process	*b*	95% CI LB	95% CI UB	*t*	*p*
**Linear Models**					
Experiment 1 (Real Coin)					
(Intercept)	26.69	24.86	28.52	28.56	<.001
Number of Successes	1.50	-0.14	3.13	1.80	.073
Experiment 2 (Virtual Coin)					
(Intercept)	39.28	37.13	41.42	35.94	<.001
Number of Successes	-2.40	-3.75	-1.05	3.49	<.001
Experiment 3 (Real Coin)					
(Intercept)	27.99	25.93	30.05	26.67	<.001
Number of Successes	0.64	-0.68	1.95	0.95	.342
Experiment 4 (Virtual Coin)					
(Intercept)	41.16	39.92	42.39	65.41	<.001
Number of Successes	-2.02	-2.80	-1.24	5.09	<.001
Experiment 5 (Virtual Coin)					
(Intercept)	31.05	29.63	32.47	42.78	<.001
Number of Successes	-1.84	-3.12	-0.56	2.82	.005
**Quadratic Models**					
Experiment 1(Real Coin)					
(Intercept)	27.29	24.93	29.65	22.66	<.001
Number of Successes	3.93	-2.31	10.17	1.24	.217
(Number of Successes)^2	-0.48	-1.66	0.70	0.79	.428
Experiment 2 (Virtual Coin)					
(Intercept)	37.10	34.46	39.73	27.65	<.001
Number of Successes	-10.92	-17.08	-4.76	3.48	<.001
(Number of Successes)^2	0.86	0.25	1.47	2.78	.005
Experiment 3 (Real Coin)					
(Intercept)	28.90	26.35	31.44	22.24	<.001
Number of Successes	4.32	-1.96	10.61	1.35	.177
(Number of Successes)^2	-0.37	-0.98	0.25	1.18	.240
Experiment 4 (Virtual Coin)					
(Intercept)	36.89	35.35	38.44	46.92	<.001
Number of Successes	-19.12	-22.98	-15.25	9.70	<.001

(Number of Successes)^2	1.70	1.32	2.07	8.85	<.001
Experiment 5 (Virtual Coin)					
(Intercept)	25.93	24.11	27.74	28.05	<.001
Number of Successes	-22.48	-27.30	-17.66	9.15	<.001
(Number of Successes)^2	4.13	3.20	5.06	8.70	<.001

Table S21: ***Linear regression analysis for beliefs that coin toss outcomes resulted from random or determined processes as a function of number of experienced successes and Reward Contingency condition (Experiment 4).*** *Reward Contingency conditions dummy-coded (reference condition: No Bonus). Contrasts are Bonferroni corrected for multiple comparisons.*

Experiment 4: Random-Determined Process by Reward Contingency Condition	*b*	95% CI LB	95% CI UB	*t*	*p*
(Intercept)	52.40	45.33	59.48	14.52	>.001
Number of Successes	-2.00	-3.35	-0.65	2.90	.004
Contingent Bonus	-2.07	-12.09	7.96	0.40	.686
Non-contingent Bonus	-1.21	-11.35	8.94	0.23	.816
Number of Successes*Contingent Bonus	0.14	-1.76	2.03	0.14	.888
Number of Successes*Non-contingent Bonus	-0.17	-2.09	1.75	0.17	.863
Simple Effects (Number of Successes by Condition)					
No Bonus	-2.00	-3.35	-0.65	2.90	.004
Contingent Bonus	-1.86	-3.19	-0.53	2.74	.006
Non-contingent Bonus	-2.17	-3.54	-0.80	3.11	.002
Contrasts					
No Bonus – Contingent Bonus	-0.14	-2.45	2.18	0.14	>.999
No Bonus – Non-contingent Bonus	0.17	-2.18	2.52	0.17	>.999
Contingent Bonus – Non-contingent Bonus	0.31	-2.02	2.64	0.31	>.999

Table S22: ***Linear regression analysis for beliefs that coin toss outcomes resulted from random or determined processes as a function of number of experienced successes, Reward Contingency condition, and Task condition (Experiment 5).*** *Reward Contingency conditions dummy-coded (reference condition: Contingent Bonus). Task conditions dummy-coded (reference condition: Observe). Contrasts are Bonferroni corrected for multiple comparisons.*

Experiment 5: Random – Determined Process	*b*	95% CI LB	95% CI UB	*t*	*p*
(Intercept)	34.89	32.83	36.95	33.23	<.001

Number of Successes (NoS)	-4.13	-5.96	-2.30	4.42	<.001
Task: Choose	-2.95	-5.86	-0.05	1.99	.047
Task: Predict	-2.63	-5.54	0.27	1.78	.076
Reward: Non-contingent (NC)	-1.88	-4.79	1.03	1.27	.205
NoS*Task:Choose	1.67	-0.94	4.27	1.26	.210
NoS*Task:Predict	0.50	-2.11	3.11	0.38	.708
NoS*Reward:NC	4.76	2.19	7.32	3.63	<.001
Task:Choose*Reward:NC	1.09	-3.02	5.20	0.52	.603
Task:Predict*Reward:NC	-0.49	-4.61	3.62	0.23	.815
NoS*Task:Choose*Reward:NC	-2.46	-6.13	1.21	1.31	.190
NoS*Task:Predict*Reward:NC	-1.27	-4.94	2.39	0.68	.496
Simple Effects (Number of Successes by Condition)					
Observe; Contingent Bonus	-4.13	-5.96	-2.30	4.42	<.001
Choose; Contingent Bonus	-2.46	-4.31	-0.61	2.60	.009
Predict; Contingent Bonus	-3.63	-5.49	-1.76	3.82	<.001
Observe; Non-contingent Bonus	0.63	-1.17	2.42	0.68	.495
Choose; Non-contingent Bonus	-0.16	-2.02	1.70	0.17	.864
Predict; Non-contingent Bonus	-0.15	-1.98	1.69	0.16	.877
Contrasts					
Contingent Bonus					
Observe – Choose	-1.67	-5.36	2.02	1.26	>.999
Observe – Predict	-0.50	-4.20	3.20	0.38	>.999
Choose – Predict	1.17	-2.55	4.89	0.87	>.999
Non-contingent Bonus					
Observe – Choose	0.79	-2.87	4.45	0.60	>.999
Observe – Predict	0.77	-2.86	4.40	0.59	>.999
Choose – Predict	-0.02	-3.71	3.68	0.01	>.999
Task: Observe					
Contingent Bonus – NC Bonus	-4.76	-8.39	-1.12	3.63	.003
Task: Choose					
Contingent Bonus – NC Bonus	-2.30	-6.01	1.42	-1.72	.775
Task: Predict					
Contingent Bonus – NC Bonus	-3.48	-7.18	0.21	-2.61	.081

Figure S10: ***Beliefs that coin toss outcomes resulted from Random (0) – Determined (100) process as a function of number of experienced successes, Reward Contingency condition, and Task condition (Experiment 5).*** *For "Observe" and "Choose" conditions, success = heads outcome. For "Predict" condition, success = correct prediction. Error bars = 95% confidence intervals.*

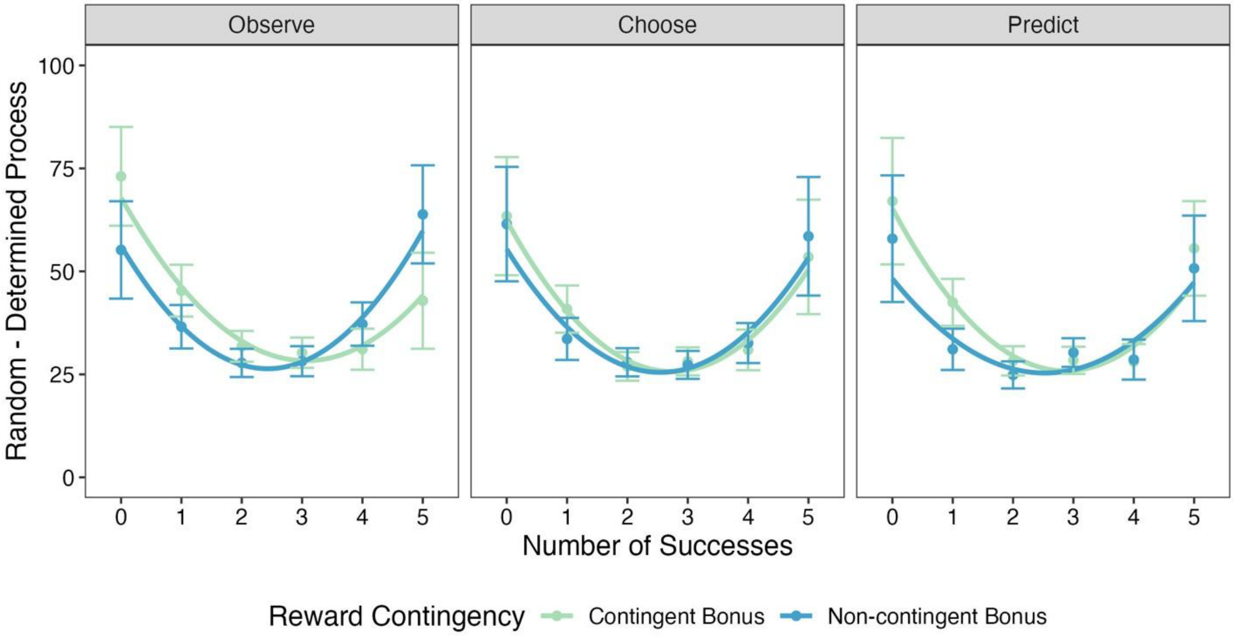

Probability Knowledge

To measure probability knowledge, we used a four-item multiple choice scale (*Table S37*). We analyzed the mean-centered number of probability questions answered correctly. Across studies, we found negative main effects of probability knowledge on self-reported ability to predict future tosses, and no interaction between probability knowledge and the number of successes on ability to predict future tosses (*Table S23, Figure S12*). Similarly, we found negative or null main effects of probability knowledge on risk-taking behavior, and no interaction effects (*Table S24, Figure S13*). In Experiment 2 and Experiment 3, we found small but significant interaction effects of probability knowledge and number of successes on the expected number of correct predictions (out of 20) such that greater knowledge of probability reduced the effect of number of successes on number of expected correct predictions (*Table S24, Figure S12*). This is consistent with the idea that people with a better understanding of probability should be more aware that randomly determined performance should not affect forecasts of future performance. However, we note that although this interaction was significant, even the participants whose probability knowledge was highest (4/4 correct) still show the success effect (*Figure S12*), and this difference did not extend to success' effects on risk-taking behavior (*Table S25, Figure S13*).

Table S23: ***Regression analyses for self-reported ability to predict future tosses as a function of number of successes and probability knowledge.*** *For Experiment 4, this result is averaged over the three levels of the Reward Contingency variable (No Bonus, Contingent Bonus, Non-contingent Bonus).*

Ability to Predict by Probability Knowledge and Number of Successes	*b*	95% CI LB	95% CI UB	*t*	*p*
Experiment 2					
(Intercept)	37.44	35.87	39.00	46.93	<.001
Number of Successes	5.20	4.22	6.19	10.35	<.001
Probability Knowledge	-3.52	-5.24	-1.80	4.01	<.001
NoS* Probability Knowledge	-0.53	-1.57	0.51	1.00	.316
Experiment 3					
(Intercept)	37.52	35.92	39.11	46.16	<.001
Number of Successes	4.22	3.20	5.24	8.12	<.001
Probability Knowledge	-4.40	-6.14	-2.67	-4.98	<.001
NoS* Probability Knowledge	-0.45	-1.56	0.65	-0.80	.422
Experiment 4					
(Intercept)	37.48	36.6	38.36	83.44	<.001
Number of Successes	5.44	4.88	5.99	19.17	<.001
Probability Knowledge	-4.26	-5.21	-3.31	8.80	<.001
NoS* Probability Knowledge	-0.43	-1.03	0.17	1.40	.162

Figure S11: ***Self-reported ability to predict future tosses by number of successes and probability knowledge (High = 4/4 correct; Low < 4/4 correct).*** *Error bars = 95% confidence intervals.*

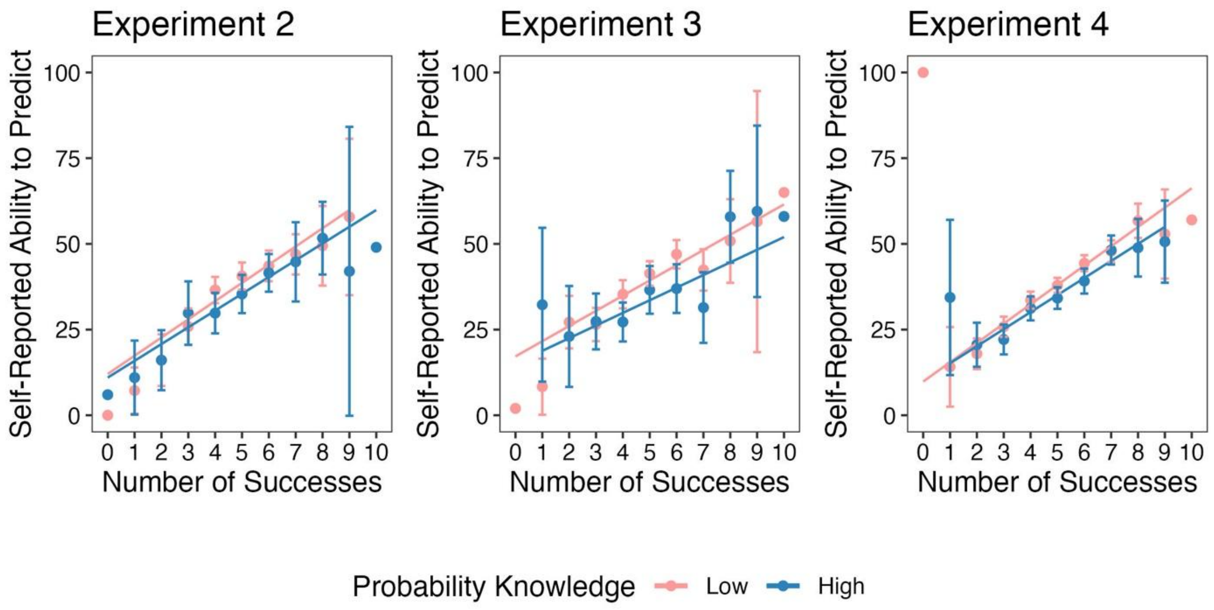

Table S24: ***Expected number of correct predictions as a function of number of successes and probability knowledge.*** *For Experiment 4, this result is averaged over the three levels of the Reward Contingency variable (No Bonus, Contingent Bonus, Non-contingent Bonus).*

Expected Correct Predictions by Probability Knowledge and Number of Successes	*b*	95% CI LB	95% CI UB	*t*	*p*
Experiment 2					
(Intercept)	9.20	9.04	9.37	110.00	<.001
Number of Successes	0.80	0.70	0.91	15.27	<.001
Probability Knowledge	-0.30	-0.48	-0.12	3.23	.001
NoS* Probability Knowledge	-0.18	-0.29	-0.07	3.21	.001
Experiment 3					
(Intercept)	9.68	9.51	9.85	113.46	<.001
Number of Successes	0.68	0.57	0.79	12.48	<.001
Probability Knowledge	-0.04	-0.22	0.14	0.43	.665
NoS* Probability Knowledge	-0.16	-0.28	-0.05	2.75	.006
Experiment 4					
(Intercept)	9.20	9.11	9.30	186.49	<.001
Number of Successes	0.69	0.63	0.75	22.14	<.001
Probability Knowledge	-0.24	-0.34	-0.13	4.47	<.001
NoS* Probability Knowledge	-0.06	-0.13	0.005	1.83	.068

Figure S12: ***Expected number of correct predictions as a function of number of successes and probability knowledge (High = 4/4 correct; Low < 4/4 correct).*** *Error bars = 95% confidence intervals.*

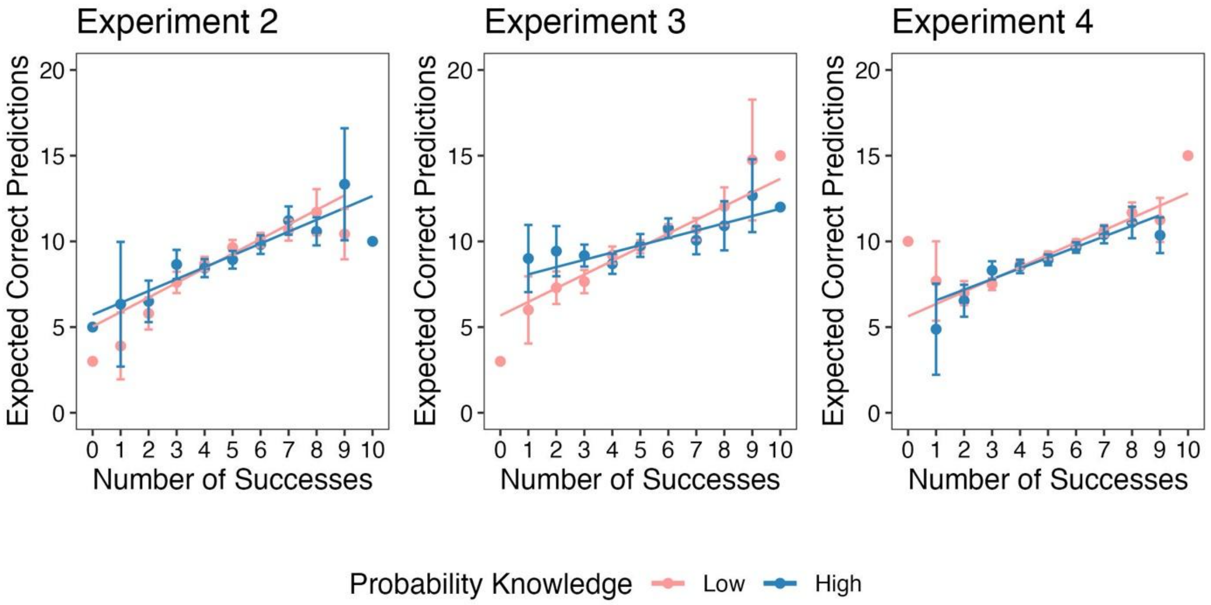

Table S25: ***Regression analyses of risk behavior as a function of number of successes and probability knowledge.*** *For Experiment 4, this result is averaged over the three levels of the Reward Contingency variable (No Bonus, Contingent Bonus, Non-contingent Bonus).*

Wagered Number of Predictions by Probability Knowledge and Number of Successes	*b*	95% CI LB	95% CI UB	*t*	*p*
Experiment 2					
(Intercept)	2.02	1.96	2.09	62.67	<.001
Number of Successes	0.17	0.12	0.23	6.13	<.001
Probability Knowledge	0.01	-0.06	0.08	0.19	.850
NoS* Probability Knowledge	-0.01	-0.07	0.05	0.47	.640
Experiment 3					
(Intercept)	2.37	2.31	2.42	82.77	<.001
Number of Successes	0.10	0.05	0.15	3.73	<.001
Probability Knowledge	-0.07	-0.13	-0.01	2.24	.025
NoS* Probability Knowledge	0.02	-0.04	0.07	0.66	.507
Experiment 4					
(Intercept)	2.06	2.02	2.10	110.20	<.001
Number of Successes	0.15	0.12	0.18	8.88	<.001
Probability Knowledge	0.005	-0.03	0.04	0.23	.814

NoS* Probability Knowledge	0.005	-0.03	0.04	0.25	.801

Figure S13: ***Wagered number of correct predictions by number of successes and probability knowledge (High = 4/4 correct; Low < 4/4 correct).*** *Error bars = 95% confidence intervals.*

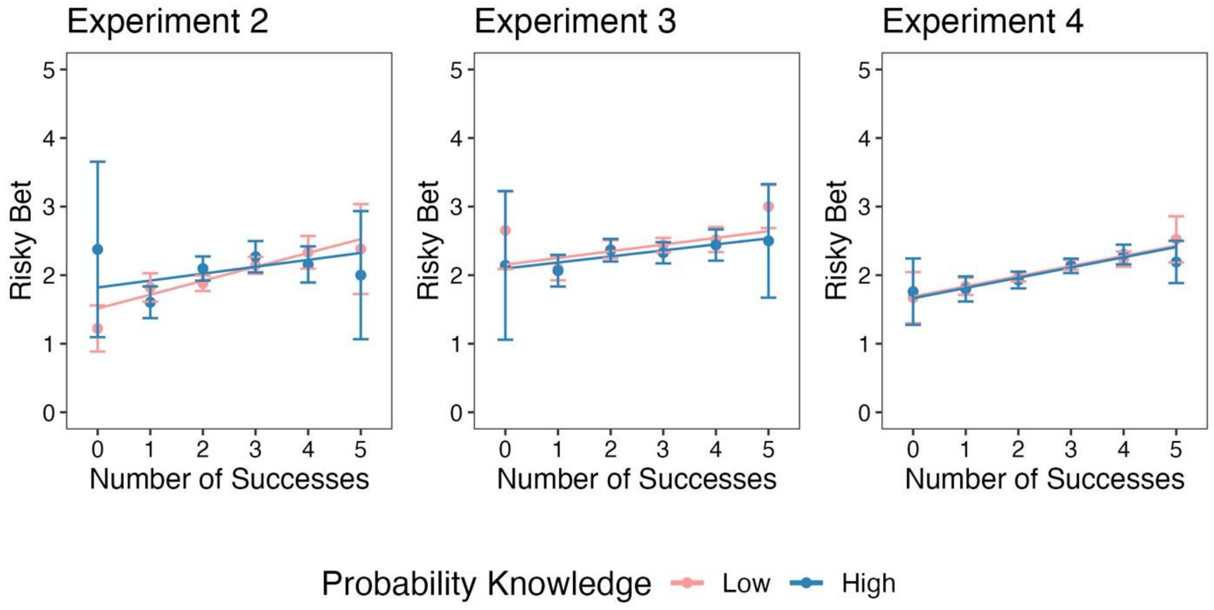

Individual Risk Preferences

We measured individual risk preferences using a single item (*Table S37*) to see if controlling for this variable affected our primary dependent measures. Individual preference for risk was positively associated with self-reported ability to predict future tosses, expected number of correct predictions and risk-taking behavior, but did not affect the effect of number of successes on all three measures (*Table S26 to Table S28*, respectively).

Table S26: ***Regression analyses of self-reported ability to predict future tosses as a function of number of successes and individual risk preferences****. For Experiment 4, this result is averaged over the three levels of the Reward Contingency variable (No Bonus, Contingent Bonus, Non-contingent Bonus).*

Ability to Predict by Number of Successes and Individual Risk Preferences	*b*	95% CI LB	95% CI UB	*t*	*p*
Experiment 2					
(Intercept)	37.43	35.87	39.00	46.91	<.001
Number of Successes	5.12	4.13	6.10	10.18	<.001
Individual Risk Preference	0.13	0.07	0.19	4.11	<.001
NoS* Individual Risk Preference	-0.01	-0.04	0.03	0.39	.696
Experiment 3					
(Intercept)	37.52	35.91	39.12	45.94	<.001

Number of Successes	4.17	3.14	5.20	7.97	<.001
Individual Risk Preference	0.13	0.07	0.20	3.89	<.001
NoS* Individual Risk Preference	0.01	-0.03	0.06	0.67	.505
Experiment 4					
(Intercept)	37.45	36.58	38.33	83.8	<.001
Number of Successes	5.30	4.75	5.85	18.78	<.001
Individual Risk Preference	0.18	0.15	0.21	10.68	<.001
NoS* Individual Risk Preference	0.02	-0.01	0.04	1.44	.149

Table S27: ***Expected correct predictions as a function of number of successes and individual risk preferences****. For Experiment 4, this result is averaged over the three levels of the Reward Contingency variable (No Bonus, Contingent Bonus, Non-contingent Bonus).*

Expected Correct Predictions by Number of Successes and Individual Risk Preferences	*b*	95% CI LB	95% CI UB	*t*	*p*
Experiment 2					
(Intercept)	9.20	9.04	9.37	110.66	<.001
Number of Successes	0.80	0.69	0.90	15.22	<.001
Individual Risk Preference	0.02	0.01	0.02	5.66	<.001
NoS* Individual Risk Preference	-0.002	-0.006	0.002	1.18	.237
Experiment 3					
(Intercept)	9.68	9.51	9.85	114.12	<.001
Number of Successes	0.68	0.57	0.78	12.46	<.001
Individual Risk Preference	0.01	0.008	0.02	4.26	<.001
NoS* Individual Risk Preference	-0.002	-0.006	0.003	0.82	.411
Experiment 4					
(Intercept)	9.20	9.11	9.30	189.57	<.001
Number of Successes	0.68	0.62	0.74	22.03	<.001
Individual Risk Preference	0.02	0.016	0.024	11.13	<.001
NoS* Individual Risk Preference	-0.002	-0.004	0.0007	1.39	.166

Table S28: ***Regression analyses of risk behavior as a function of number of successes and individual risk preferences****. For Experiment 4, this result is averaged over the three levels of the Reward Contingency variable (No Bonus, Contingent Bonus, Non-contingent Bonus).*

Risk by Number of Successes and Individual Risk Preferences	*b*	95% CI LB	95% CI UB	*t*	*p*

Experiment 2					
(Intercept)	2.02	1.96	2.08	63.57	<.001
Number of Successes	0.17	0.11	0.22	5.94	<.001
Individual Risk Preference	0.01	0.004	0.01	5.48	<.001
NoS* Individual Risk Preference	-0.0002	-0.002	0.002	-0.24	.811
Experiment 3					
(Intercept)	2.37	2.31	2.42	85.14	<.001
Number of Successes	0.09	0.04	0.14	3.68	<.001
Individual Risk Preference	0.01	0.01	0.01	7.78	<.001
NoS* Individual Risk Preference	-0.0006	-0.003	0.001	-0.55	.583
Experiment 4					
(Intercept)	2.06	2.02	2.10	111.72	<.001
Number of Successes	0.14	0.11	0.17	8.40	<.001
Individual Risk Preference	0.01	0.01	0.01	9.69	<.001
NoS* Individual Risk Preference	0.0002	-0.001	0.001	0.29	.775

Affective State

Although it was not pre-registered, tested whether reward amount (plausibly related to participants' mood) affected expectations of future performance and risk-taking behavior. As described above and in the main text, we did not observe a significant effect of prediction performance on participants' beliefs and risk behavior in the *Non-Contingent Bonus* conditions in Experiment 4 and Experiment 5. However, these rewards were uncorrelated with performance, and it could be the case that participants in these conditions who earned greater rewards took greater risks and had more optimistic beliefs about future performance due to a positive mood induced by the reward.

For participants who experienced *Non-contingent* rewards, we tested this possibility for risk behavior in Experiment 4 and for all other measured variables in Experiment 5 (limiting analysis to participants in the *Predict* condition). For each model we regressed number of successes and bonus payout (in USD) onto the dependent variable. If earning a reward (unrelated to performance) induces a positive mood that in turn affects expectations of future performance and risk-taking behavior, we should see significant effects of bonus amount on the measured variables. Results of these analyses are shown in Table S29. Across all analyses, reward amount was never a significant predictor of any of the dependent measures.

Table S29: ***Regression analyses of dependent variables for participants in Non-contingent Rewards Conditions as a function of number of successes and bonus amount (in dollars)****. For Experiment 5, this analysis is limited to participants in the Predict condition.*

DV by Number of Successes and Bonus Amount	*b*	95% CI LB	95% CI UB	*t*	*p*

Exp 4 – Risk Behavior					
(Intercept)	2.09	2.03	2.16	63.00	<.001
Number of Successes	0.17	0.11	0.23	5.67	<.001
Bonus Payout	0.007	-0.05	0.07	0.24	.813
Exp 5 – Ability to Predict					
(Intercept)	33.62	32.07	35.17	42.68	<.001
Number of Successes	7.18	5.81	8.56	10.26	<.001
Bonus Payout	-0.45	-1.84	0.95	0.63	.530
Exp 5 –Expected Correct Predictions					
(Intercept)	9.46	9.29	9.62	110.00	<.001
Number of Successes	0.88	0.73	1.03	11.51	<.001
Bonus Payout	-0.05	-0.20	0.10	0.67	.501
Exp 5 –WTB on Predictions					
(Intercept)	26.22	23.98	28.46	22.98	<.001
Number of Successes	2.63	0.63	4.62	2.59	.010
Bonus Payout	-1.73	-3.74	0.29	1.68	.093
Exp 5 –Luck/Skill Attributions					
(Intercept)	16.15	14.87	17.42	24.84	<.001
Number of Successes	2.60	1.47	3.74	4.51	<.001
Bonus Payout	0.17	-0.98	1.32	0.29	.770
Exp 5 –Expected Number of Heads					
(Intercept)	10.15	10.01	10.28	147.59	<.001
Number of Successes	0.39	0.27	0.51	6.42	<.001
Bonus Payout	-0.04	-0.16	0.08	0.63	.530
Exp 5 –WTB on Heads					
(Intercept)	26.84	24.54	29.14	22.89	<.001
Number of Successes	0.68	-1.36	2.72	0.66	.512
Bonus Payout	-1.91	-3.98	0.16	1.81	.071

Prediction Complexity

We estimated the algorithmic complexity of participants' prediction sequences using the ACSS package in R (*46*). Algorithmic complexity is an estimation of the shortest computer program that produces a given string and has been advocated as a model of human randomness judgments (*59*). In other words, the more algorithmically complex a string is, the more random people perceive it to be. For example, the coin toss sequence HHHHHHHHHH is less complex than the sequence HTHHTHTTTH and would be perceived as less random by most people. We reasoned that participants who are making more complex predictions and happened to be

"successful" in their predictions are more likely to detect a spurious correlation between their predictions and outcomes. In the extreme case where participants are making the same prediction (see the main text), there is no variance in their predictions and no correlation could be detected in principle.

Across all five studies, regression models predicting ability to predict from number of successes, complexity, and their interaction revealed that complexity positively moderates the effect of success on perceived ability to predict future tosses (*Table S30, Figure S14*) and expected number of correct predictions (*Table S31, Figure S15*). Participants who generated more complex prediction strings showed a greater influence of the number of successes on their own perceived ability to predict future tosses. In general, participants who generated more complex prediction strings and were successful were more confident in their ability to predict relative to participants who experienced the same level of success but who generated less complex prediction strings, and the opposite was true for participants who were unsuccessful. Similar effects were obtained for risk-taking behavior (*Table S32, Figure S16*).

Table S30: ***Regression analyses of self-reported ability to predict future tosses as a function of number of successes and prediction complexity.*** *For Experiment 4, this result is averaged over the three levels of the Reward Contingency variable (No Bonus, Contingent Bonus, Non-contingent Bonus). For Experiment 5, this result is drawn from the subset of participants who experienced the Predict condition (n = 2010) and is averaged over the two levels of the Reward Contingency variable (Contingent Bonus, Non-contingent Bonus).*

Ability to Predict by Prediction Complexity	*b*	95% CI LB	95% CI UB	*t*	*p*
Experiment 1					
(Intercept)	39.13	37.7	40.56	53.59	<.001
Number of Successes	6.09	2.48	9.70	3.31	.001
Complexity	5.85	4.58	7.13	8.98	<.001
NoS*Complexity	3.95	0.80	7.10	2.46	.014
Experiment 2					
(Intercept)	37.46	35.89	39.04	46.69	<.001
Number of Successes	5.17	4.18	6.16	10.23	<.001
Complexity	0.22	-0.54	0.99	0.57	.570
NoS*Complexity	0.60	0.10	1.10	2.33	.020
Experiment 3					
(Intercept)	37.58	35.98	39.18	46.1	<.001
Number of Successes	4.34	3.31	5.36	8.31	<.001
Complexity	1.42	0.62	2.21	3.48	.001
NoS*Complexity	0.64	0.14	1.14	2.50	.013
Experiment 4					
(Intercept)	37.45	36.56	38.34	82.57	<.001
Number of Successes	5.37	4.81	5.93	18.77	<.001
Complexity	0.93	0.50	1.35	4.24	<.001

NoS*Complexity	0.34	0.06	0.62	2.38	.017
Experiment 5 (Virtual Coin)					
(Intercept)	31.65	30.56	32.74	57.03	<.001
Number of Successes	8.00	7.02	8.98	16.06	<.001
Complexity	7.02	4.47	9.58	5.39	<.001
NoS*Complexity	3.46	1.06	5.86	2.82	.005

Figure S14: ***Effects of algorithmic complexity on perceived ability to predict future tosses at different levels of success.*** *Success levels are grouped for ease of interpretation.*

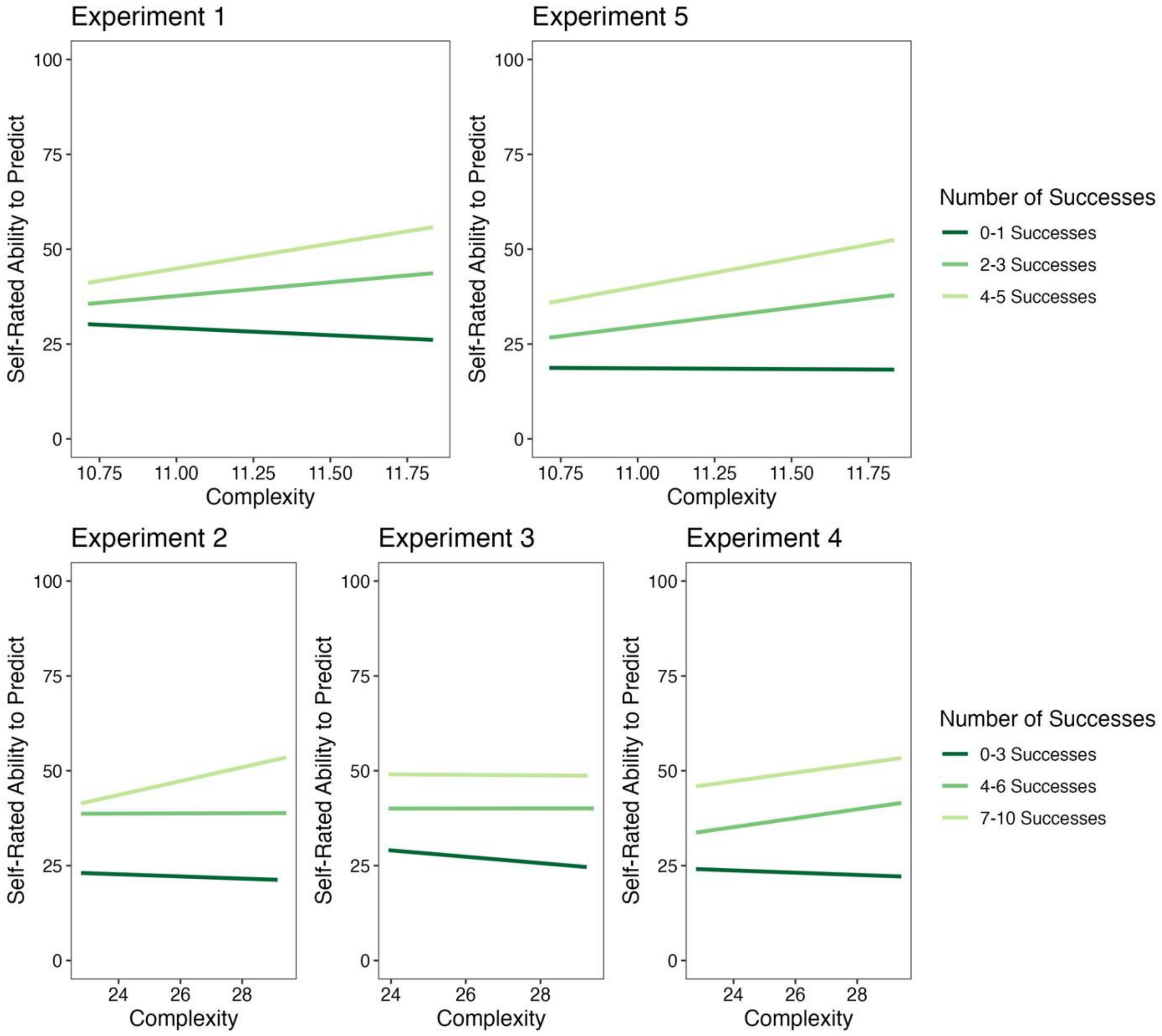

Table S31: ***Regression analyses of expected number of correct predictions as a function of number of successes and prediction complexity.*** *For Experiment 4, this result is averaged over the three levels of the Reward Contingency variable (No Bonus, Contingent Bonus, Non-contingent Bonus). For Experiment 5, this result is drawn from the subset of participants who experienced the Predict condition (n = 2010) and is averaged over the two levels of the Reward Contingency variable (Contingent Bonus, Non-contingent Bonus).*

Expected Correct Predictions by Prediction Complexity	*b*	95% CI LB	95% CI UB	*t*	*p*
Experiment 1					
(Intercept)	9.65	9.47	9.82	108.74	<.001
Number of Successes	1.21	1.06	1.37	15.16	<.001
Complexity	-0.16	-0.61	0.28	0.72	.470
NoS*Complexity	1.12	0.72	1.50	5.60	<.001
Experiment 2					
(Intercept)	9.21	9.04	9.37	109.20	<.001
Number of Successes	0.80	0.70	0.91	15.12	<.001
Complexity	-0.03	-0.11	0.05	0.80	.425
NoS*Complexity	0.07	0.01	0.11	2.44	.015
Experiment 3					
(Intercept)	9.69	9.52	9.96	114.41	<.001
Number of Successes	0.68	0.58	0.79	12.60	<.001
Complexity	-0.11	-0.19	-0.02	2.51	.012
NoS*Complexity	0.11	0.06	0.17	4.27	<.001
Experiment 4					
(Intercept)	9.20	9.10	9.30	186.03	<.001
Number of Successes	0.69	0.63	0.75	22.06	<.001
Complexity	-0.07	-0.12	-0.02	2.96	.003
NoS*Complexity	0.03	0.002	0.06	2.09	.037
Experiment 5 (Virtual Coin)					
(Intercept)	9.32	9.20	9.44	153.90	<.001
Number of Successes	0.99	0.89	1.10	18.24	<.001
Complexity	-0.11	-0.39	0.17	0.78	.437
NoS*Complexity	0.41	0.15	0.67	3.06	.002

Figure S15: ***Effects of algorithmic complexity on expected number of correct predictions at different levels of success.*** *Success levels are grouped for ease of interpretation.*

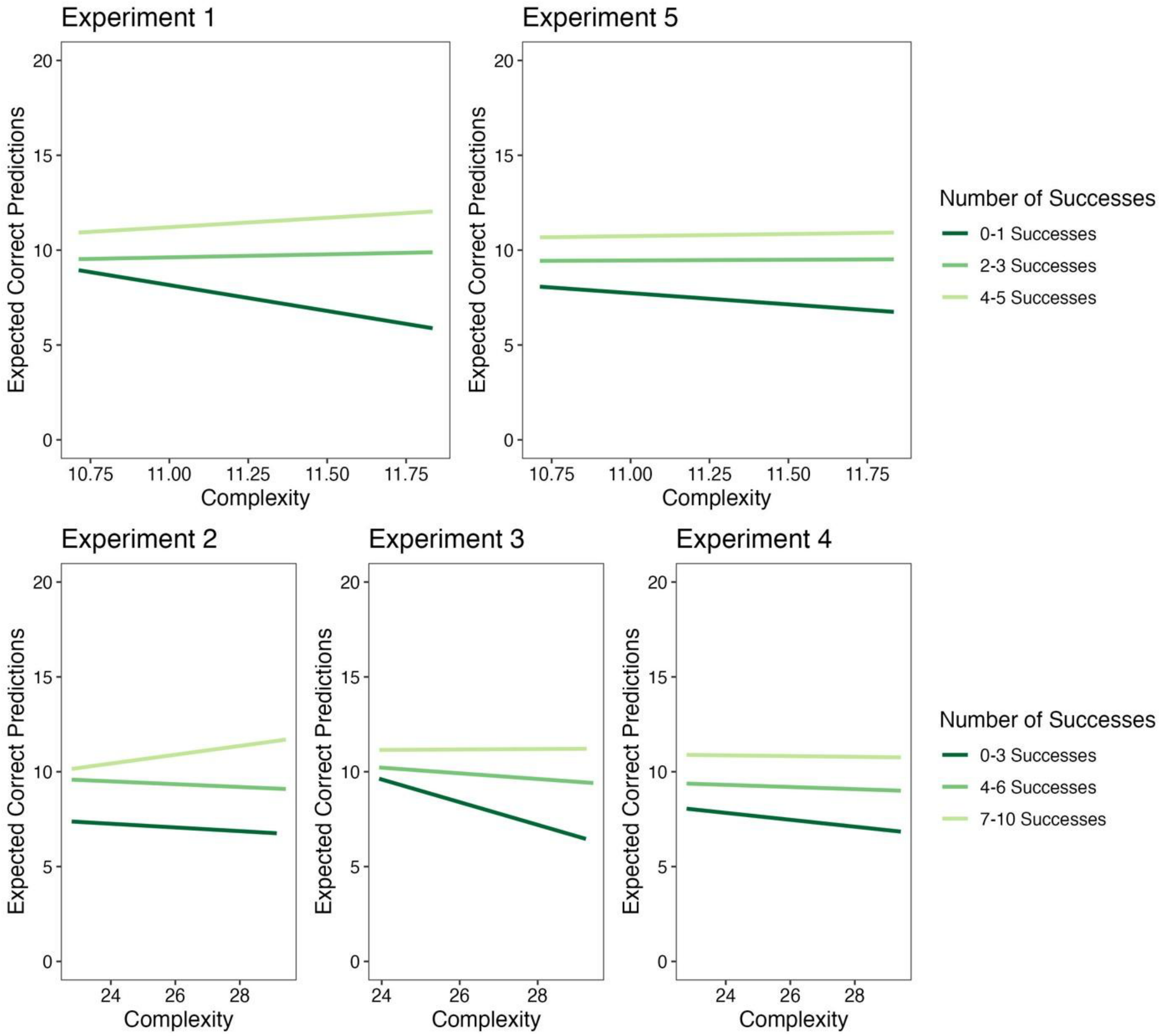

Table S32: ***Regression analyses of risk behavior as a function of number of successes and prediction complexity.*** *For Experiment 1, participants either accepted or rejected a risky bet (binary choice). For Experiments 2-4, participants wagered based on their expected future rate of prediction accuracy. For Experiment 5, participants self-reported their willingness to bet on their predictions (continuous scale). For Experiment 4, this result is averaged over the three levels of the Reward Contingency variable (No Bonus, Contingent Bonus, Non-contingent Bonus). For Experiment 5, this result is drawn from the subset of participants who experienced the Predict condition (n = 2010) and is averaged over the two levels of the Reward Contingency variable (Contingent Bonus, Non-contingent Bonus).*

Risk by Prediction Complexity	*b*	95% CI LB	95% CI UB	*t*	*p*
Experiment 1					
(Intercept)	0.61	0.58	0.64	39.26	<.001
Number of Successes	-0.05	-0.12	0.03	1.16	0.244
Complexity	0.004	-0.02	0.03	0.27	0.787
NoS*Complexity	0.01	-0.06	0.07	0.19	0.848
Experiment 2					
(Intercept)	2.01	1.94	2.07	61.93	<.001
Number of Successes	0.18	0.12	0.23	6.24	<.001
Complexity	0.004	-0.16	0.17	0.05	.960
NoS*Complexity	0.24	0.10	0.39	3.35	.001
Experiment 3					
(Intercept)	2.36	2.31	2.42	82.60	<.001
Number of Successes	0.10	0.05	0.15	3.72	<.001
Complexity	0.0006	-0.15	0.15	0.01	.994
NoS*Complexity	0.19	0.06	0.33	2.85	.005
Experiment 4					
(Intercept)	2.05	2.02	2.09	109.11	<.001
Number of Successes	0.15	0.12	0.18	8.88	<.001
Complexity	0.04	-0.05	0.14	0.90	.368
NoS*Complexity	0.12	0.04	0.20	2.92	.004
Experiment 5 (Virtual Coin)					
(Intercept)	26.96	25.38	28.54	33.46	<.001
Number of Successes	3.66	2.24	5.08	5.06	<.001
Complexity	9.40	5.69	13.11	4.97	<.001
NoS*Complexity	0.07	-3.42	3.56	0.04	.969

Figure S16: ***Effects of algorithmic complexity on risk behavior at different levels of success.*** *Success levels are grouped for ease of interpretation.*

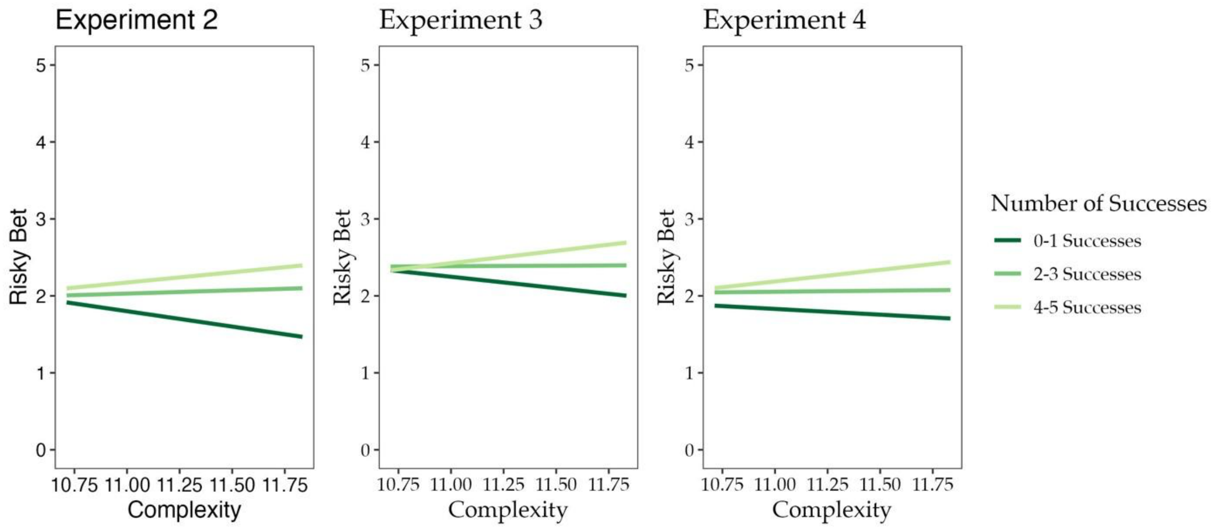

Prediction Changes

We computed the effects of the number of successes on our primary dependent variables according to whether or not participants changed their predicted outcome between trials even once as a simplified measure of prediction complexity. Results aggregated across studies are reported in the main text. We find significant interactions between number of successes and prediction changes such that participants who changed their prediction even once were more sensitive to number of successes in their ratings of self-reported ability to predict future tosses (*Table S33, Figure S17*), expected number of correct predictions (*Table S34, Figure S18*), and risk-taking behavior (except for Experiment 1 where the main effects were null, see *Table S35, Figure S19*).

Table S33: ***Regression analyses and simple effects of self-reported ability to predict future tosses as a function of number of successes and prediction changes (Change vs. No Change).*** *Change conditions dummy-coded (reference condition = "No Change"). For Experiment 4, this result is averaged over the three levels of the Reward Contingency variable (No Bonus, Contingent Bonus, Non-contingent Bonus). For Experiment 5, this result is drawn from the subset of participants who experienced the Predict condition (n = 2010) and is averaged over the two levels of the Reward Contingency variable (Contingent Bonus, Non-contingent Bonus).*

Ability to Predict by Changes	*b*	95% CI LB	95% CI UB	*t*	*p*
Experiment 1					
(Intercept)	35.27	32.74	37.80	27.35	<.001
Number of Successes	3.73	1.58	5.87	3.40	.001
Changes (Change vs. No Change)	5.72	2.65	8.79	3.66	<.001
NoS*Changes	3.18	0.51	5.85	2.33	.020
Simple Effects: No Change	3.73	1.58	5.87	3.40	.001
Simple Effects: Change	6.90	5.32	8.49	8.54	<.001
Experiment 2					
(Intercept)	35.82	32.05	39.59	18.65	<.001

Number of Successes	1.96	-0.55	4.47	1.53	.125
Changes (Change vs. No Change)	2.07	-2.07	6.22	0.98	.327
NoS*Changes	3.84	1.12	6.57	2.76	.006
Simple Effects: No Change	1.96	-0.55	4.47	1.53	.125
Simple Effects: Change	5.80	4.73	6.88	10.56	<.001
Experiment 3					
(Intercept)	29.96	26.08	33.85	15.13	<.001
Number of Successes	1.25	-1.13	3.62	1.03	.303
Changes (Change vs. No Change)	9.25	4.99	13.51	4.26	<.001
NoS*Changes	3.78	1.15	6.41	2.82	.005
Simple Effects: No Change	1.25	-1.13	3.62	1.03	.303
Simple Effects: Change	5.02	3.90	6.15	8.74	<.001
Experiment 4					
(Intercept)	32.13	30.17	34.10	32.00	<.001
Number of Successes	3.35	2.02	4.67	4.96	<.001
Changes (Change vs. No Change)	6.67	4.46	8.87	5.93	<.001
NoS*Changes	2.50	1.04	3.96	3.36	.001
Simple Effects: No Change	3.35	2.02	4.67	4.96	<.001
Simple Effects: Change	5.85	5.23	6.47	18.61	<.001
Experiment 5 (Virtual Coin)					
(Intercept)	26.32	24.42	28.21	27.23	<.001
Number of Successes	5.28	3.56	6.99	6.04	<.001
Changes (Change vs. No Change)	7.88	5.57	10.19	6.69	<.001
NoS*Changes	3.94	1.86	6.02	3.71	<.001
Simple Effects: No Change	5.28	3.56	6.99	6.04	<.001
Simple Effects: Change	9.22	8.03	10.4	15.28	<.001

Figure S17: ***Self-reported ability to predict future tosses as a function of number of successes and prediction changes (Change vs. No Change).*** *For Experiment 4, this result is averaged over the three levels of the Reward Contingency variable (No Bonus, Contingent Bonus, Non-contingent Bonus). For Experiment 5, this result is drawn from the subset of participants who experienced the Predict condition (n = 2010) and is averaged over the two levels of the Reward Contingency variable (Contingent Bonus, Non-contingent Bonus).*

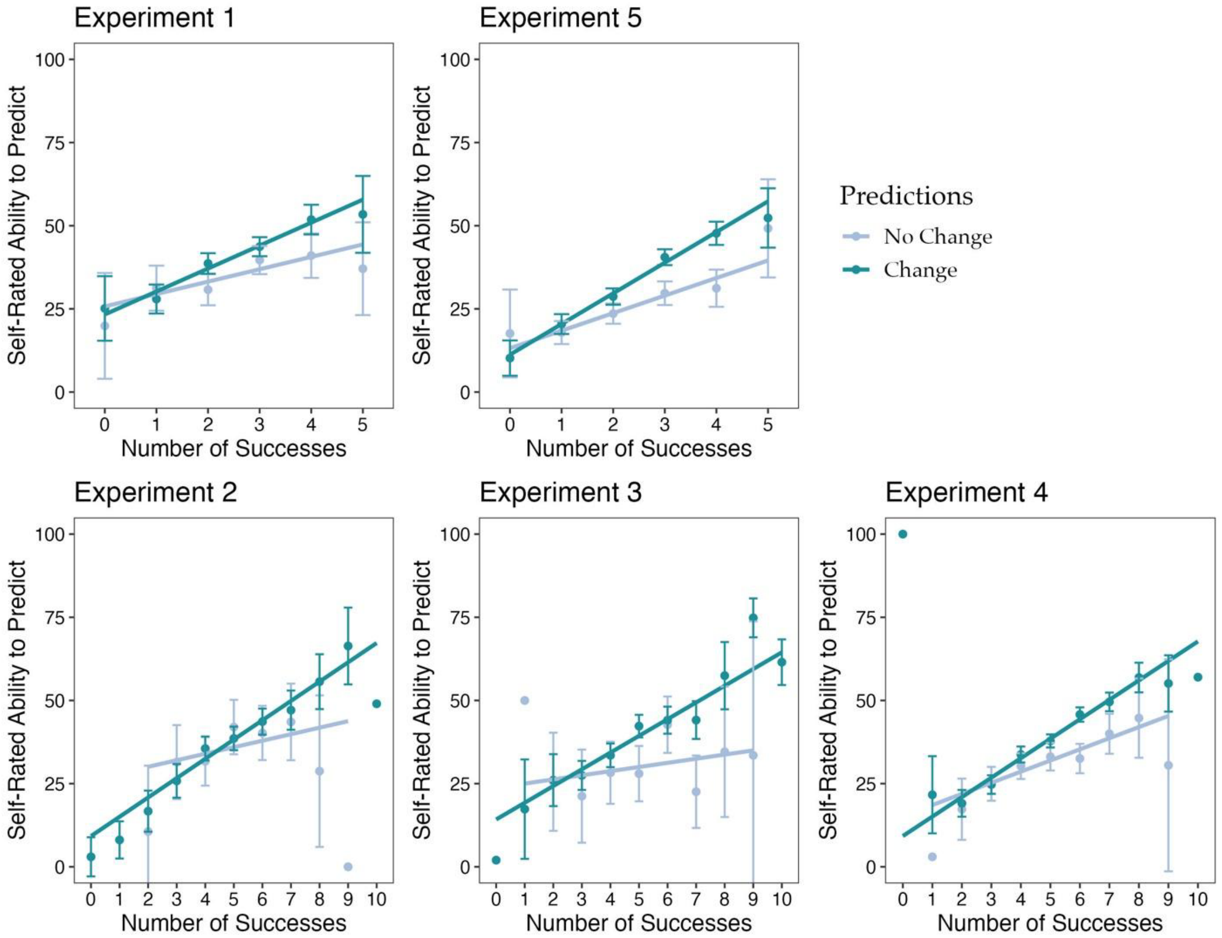

Table S34: ***Regression analyses and simple effects of expected number of correct predictions as a function of number of successes and prediction changes (Change vs. No Change).*** *Change conditions dummy-coded (reference condition = "No Change"). For Experiment 4, this result is averaged over the three levels of the Reward Contingency variable (No Bonus, Contingent Bonus, Non-contingent Bonus). For Experiment 5, this result is drawn from the subset of participants who experienced the Predict condition (n = 2010) and is averaged over the two levels of the Reward Contingency variable (Contingent Bonus, Non-contingent Bonus).*

Number of Correct Predictions by Changes	*b*	95% CI LB	95% CI UB	*t*	*p*
Experiment 1					
(Intercept)	9.72	9.41	10.04	60.11	<.001

Number of Successes	0.57	0.30	0.84	4.18	<.001
Changes (Change vs. No Change)	-0.10	-0.49	0.27	0.56	.579
NoS*Changes	0.96	0.63	1.29	5.67	<.001
Simple Effects: No Change	0.57	0.30	0.84	4.18	<.001
Simple Effects: Change	1.53	1.34	1.72	15.53	<.001
Experiment 2					
(Intercept)	9.25	8.86	9.65	45.92	<.001
Number of Successes	0.36	0.09	0.62	2.67	.008
Changes (Change vs. No Change)	-0.04	-0.48	0.40	0.18	.857
NoS*Changes	0.53	0.24	0.82	3.64	<.001
Simple Effects: No Change	0.36	0.09	0.62	2.67	.008
Simple Effects: Change	0.89	0.78	1.00	15.41	<.001
Experiment 3					
(Intercept)	9.93	9.52	10.33	47.79	<.001
Number of Successes	0.27	0.02	0.52	2.12	.034
Changes (Change vs. No Change)	-0.28	-0.72	0.17	1.22	.223
NoS*Changes	0.49	0.22	0.77	3.51	<.001
Simple Effects: No Change	0.27	0.02	0.52	2.12	.034
Simple Effects: Change	0.76	0.64	0.88	12.64	<.001
Experiment 4					
(Intercept)	9.53	9.32	9.74	86.82	<.001
Number of Successes	0.50	0.36	0.65	6.82	<.001
Changes (Change vs. No Change)	-0.42	-0.66	-0.17	3.38	<.001
NoS*Changes	0.23	0.07	0.39	2.80	.005
Simple Effects: No Change	0.50	0.36	0.65	6.82	<.001
Simple Effects: Change	0.73	0.66	0.80	21.25	<.001
Experiment 5 (Virtual Coin)					
(Intercept)	9.43	9.23	9.65	89.06	<.001
Number of Successes	0.79	0.60	0.97	8.18	<.001
Changes (Change vs. No Change)	-0.18	-0.43	0.07	1.40	.161
NoS*Changes	0.32	0.09	0.55	2.75	.006
Simple Effects: No Change	0.79	0.60	0.97	8.18	<.001
Simple Effects: Change	1.11	0.98	1.24	16.71	<.001

Figure S18: ***Expected correct predictions as a function of number of successes and prediction changes (Change vs. No Change).*** *For Experiment 4, this result is averaged over the three levels of the Reward Contingency variable (No Bonus, Contingent Bonus, Non-contingent Bonus). For Experiment 5, this result is drawn from the subset of participants who experienced the Predict condition (n = 2010) and is averaged over the two levels of the Reward Contingency variable (Contingent Bonus, Non-contingent Bonus).*

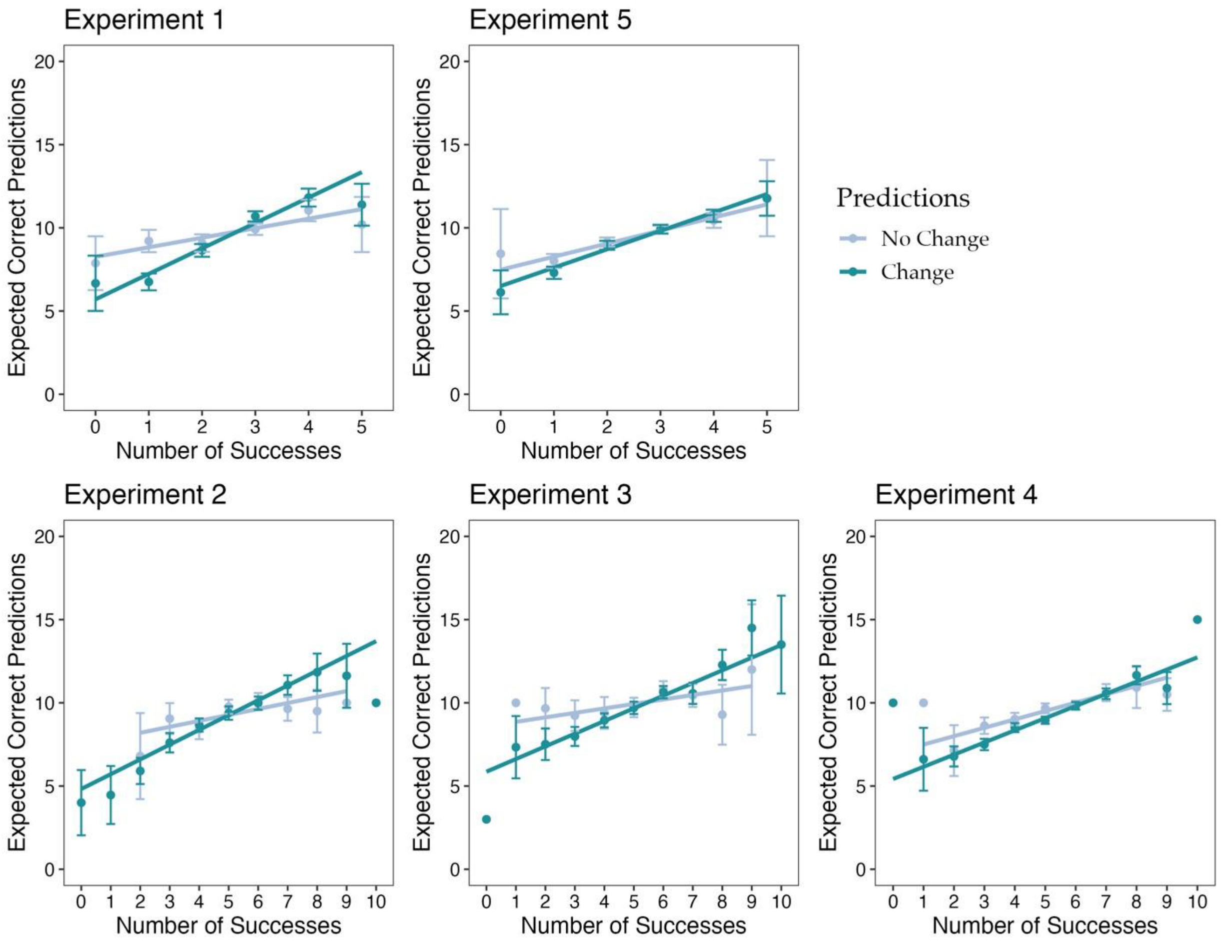

Table S35: ***Regression analyses for risk-taking behavior as a function of number of experienced successes and prediction changes (Change vs. No Change).*** *For Experiment 1, participants either accepted or rejected a risky bet (binary choice). For Experiments 2-4, participants wagered based on their expected future rate of prediction accuracy. For Experiment 5, participants self-reported their willingness to bet on their predictions (continuous scale). For Experiment 4, this result is averaged over the three levels of the Reward Contingency variable (No Bonus, Contingent Bonus, Non-contingent Bonus). For Experiment 5, this result is drawn from the subset of participants who experienced the Predict condition (n = 2010) and is averaged over the two levels of the Reward Contingency variable (Contingent Bonus, Non-contingent Bonus).*

Risk by Changes	*b*	95% CI LB	95% CI UB	*t*	*p*
Experiment 1					

(Intercept)	0.63	0.58	0.68	23.04	<.001
Number of Successes	0.004	-0.04	0.05	0.19	.850
Changes (Change vs. No Change)	-0.03	-0.10	0.03	-0.95	.344
NoS*Changes	0.00	-0.06	0.06	-0.05	.958
Simple Effects: No Change	0.004	-0.04	0.05	0.19	.850
Simple Effects: Change	0.002	-0.03	0.04	0.17	.868
Experiment 2					
(Intercept)	1.99	1.87	2.12	31.87	<.001
Number of Successes	0.04	-0.07	0.14	0.71	.480
Changes (Change vs. No Change)	0.02	-0.12	0.16	0.28	.783
NoS*Changes	0.19	0.07	0.32	3.08	.002
Simple Effects: No Change	0.04	-0.07	0.14	0.71	.480
Simple Effects: Change	0.23	0.16	0.30	6.82	<.001
Experiment 3					
(Intercept)	2.36	2.25	2.47	41.08	<.001
Number of Successes	-0.01	-0.11	0.09	-0.10	.922
Changes (Change vs. No Change)	0.01	-0.12	0.14	0.08	.934
NoS*Changes	0.13	0.02	0.25	2.27	.023
Simple Effects: No Change	-0.01	-0.11	0.09	-0.10	.922
Simple Effects: Change	0.13	0.07	0.19	4.28	<.001
Experiment 4					
(Intercept)	2.02	1.95	2.09	59.63	<.001
Number of Successes	0.08	0.02	0.14	2.77	.006
Changes (Change vs. No Change)	0.05	-0.03	0.13	1.23	.218
NoS*Changes	0.10	0.03	0.17	2.81	.005
Simple Effects: No Change	0.08	0.02	0.14	2.77	.006
Simple Effects: Change	0.18	0.14	0.22	8.73	<.001
Experiment 5 (Virtual Coin)					
(Intercept)	20.85	18.09	23.61	14.80	<.001
Number of Successes	2.50	-0.003	4.99	1.96	.050
Changes (Change vs. No Change)	9.01	5.64	12.38	5.25	<.001
NoS*Changes	3.94	1.86	6.02	3.71	<.001
Simple Effects: No Change	2.50	-0.003	4.99	1.96	.050
Simple Effects: Change	4.13	2.41	5.85	4.70	<.001

Figure S19: ***Wagered number of correct predictions as a function of number of successes and prediction change (Change vs. No Change).*** *For Experiment 4, this result is averaged over the three levels of the Reward Contingency variable (No Bonus, Contingent Bonus, Non-contingent Bonus).*

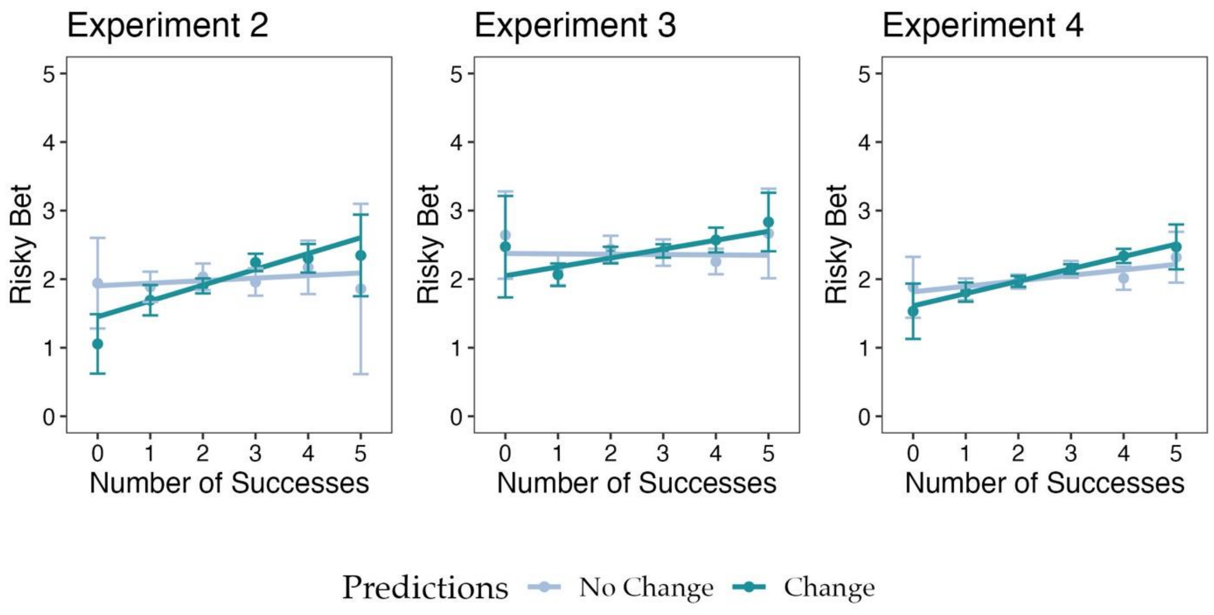

Correlations of Dependent Measures

We computed the correlations between each of our dependent measures as a way of helping to contextualize the primary effects reported in the main text. For example, although we observe mixed results of luck and skill attributions, when measured separately, skill attributions are correlated with self-reported ability to predict whereas luck attributions are not (*Table S39*). Abbreviations for variables are as follows: AbPred = Self-reported ability to predict; ExpPred = Number of expected correct predictions (out of 20); L-S = Luck/Skill attributions as a single continuous measure; RandDet = Random/Determined process beliefs; Risk = Behavioral measure of Risk (varies by Experiment, see *SM* pp. 10-14, *Table S39*); Skill = Skill attributions; Luck = Luck attributions; ExpHeads = Expected number of heads outcomes (out of 20); Conf = Confidence in advice; WTB_P = Willingness to bet on predictions; WTB_H = Willingness to bet on heads.

Table S36. Correlation matrices for dependent measures across all experiments.

Experiment 1

	AbPred	ExpPred	Luck-Skill	RandDet	Risk
AbPred	1.00				
ExpPred	0.50	1.00			
Luck-Skill	0.38	0.26	1.00		
RandDet	0.24	0.16	0.39	1.00	
Risk	0.13	0.13	0.10	0.00	1.00

Experiment 2

	AbPred	ExpPred	Skill	Luck	RandDet	Risk	ExpHeads
AbPred	1.00						
ExpPred	0.49	1.00					
Skill	0.49	0.31	1.00				
Luck	-0.11	-0.02	-0.18	1.00			
RandDet	0.12	0.05	0.15	-0.24	1.00		
Risk	0.06	0.16	0.05	-0.05	0.08	1.00	
ExpHeads	0.19	0.32	0.18	0.08	0.02	0.12	1.00

Experiment 3

	AbPred	ExpPred	Skill	Luck	RandDet	Risk
AbPred	1.00					
ExpPred	0.44	1.00				
Skill	0.34	0.12	1.00			
Luck	0.01	0.02	-0.04	1.00		
RandDet	0.23	0.15	0.23	-0.12	1.00	
Risk	0.08	0.21	0.06	0.02	0.08	1.00

Experiment 4

	AbPred	ExpPred	Skill	Luck	RandDet	Risk	Conf
AbPred	1.00						
ExpPred	0.48	1.00					
Skill	0.48	0.34	1.00				
Luck	-0.09	-0.03	-0.23	1.00			
RandDet	0.14	0.04	0.19	-0.21	1.00		
Risk	0.05	0.14	0.03	0.02	0.02	1.00	
Conf	0.38	0.34	0.34	-0.13	0.05	0.06	1.00

Experiment 5

	AbPred	ExpPred	L-S	RandDet	WTB_P	WTB_H	ExpHeads
AbPred	1.00						
ExpPred	0.45	1.00					
Luck-Skill	0.45	0.31	1.00				
RandDet	0.15	0.07	0.29	1.00			
WTB_Preds	0.25	0.27	0.19	0.03	1.00		
WTB_Heads	0.19	0.20	0.17	0.02	0.75	1.00	
ExpHeads	0.18	0.25	0.15	0.04	0.12	0.16	1.00

Hot Hand Effects

We tested for the presence of "hot hand" effects in our data by computing the length of each participant's ending streak of successes (0 - 5) and including this variable in our models along with overall number of successes. We limited these analyses to variables measured after five trials (all variables in Experiments 1 and 5, risky bet in Experiments 2-4) in order to compute success rate and ending streak from the exact same set of trials. These measures are correlated (r_{Exp1}= .63; r_{Exp2}= .64; r_{Exp3}= .60; r_{Exp4}= .62; r_{Exp5}= .62), but not such that regression analysis is uninterpretable.

Table S37. Models testing for effects of success controlling for ending streaks.

Dependent Measures by Success and Ending Streak Length	*b*	95% CI LB	95% CI UB	*t*	*p*
Exp 1 - Ability to Predict					
(Intercept)	39.22	37.78	40.66	53.35	<.001
Number of Successes	6.10	4.45	7.74	7.26	<.001
Ending Streak of Successes	-0.31	-1.70	1.08	0.43	.664
Exp 1 - Expected Correct Predictions					
(Intercept)	9.66	9.49	9.84	107.55	<.001
Number of Successes	1.34	1.14	1.54	13.00	<.001
Ending Streak of Successes	-0.19	-0.36	-0.02	2.21	.028
Exp 1 - Risky Bet					
(Intercept)	0.60	0.58	0.64	39.35	<.001
Number of Successes	-0.01	-0.05	0.02	0.78	.438
Ending Streak of Successes	0.02	-0.01	0.05	1.50	.134
Exp 1 - Luck/Skill Attributions					
(Intercept)	23.16	21.74	24.58	32.01	<.001
Number of Successes	2.92	1.29	4.54	3.53	<.001
Ending Streak of Successes	-1.01	-2.38	0.36	1.50	.149
Exp 2 - Risky Bet					
(Intercept)	2.02	1.96	2.09	62.72	<.001
Number of Successes	0.18	0.11	0.25	4.92	<.001
Ending Streak of Successes	-0.01	-0.07	0.05	0.33	.741
Exp 3 - Risky Bet					
(Intercept)	2.37	2.31	2.43	82.65	<.001
Number of Successes	0.07	0.01	0.13	2.14	.033
Ending Streak of Successes	0.04	-0.02	0.09	1.27	.206
Exp 4 - Risky Bet					

(Intercept)	2.06	2.31	2.43	110.26	<.001
Number of Successes	0.15	0.11	0.19	7.01	<.001
Ending Streak of Successes	-0.001	-0.04	0.03	0.06	.950
Exp 5 - Ability to Predict					
(Intercept)	31.96	30.87	33.06	57.37	<.001
Number of Successes	8.10	6.85	9.35	12.74	<.001
Ending Streak of Successes	0.26	-0.82	1.34	0.48	.634
Exp 5 - Expected Correct Predictions					
(Intercept)	9.35	9.24	9.47	154.98	<.001
Number of Successes	1.01	0.88	1.15	14.67	<.001
Ending Streak of Successes	-0.02	-0.14	0.09	0.42	.677
Exp 5 - WTB Predictions					
(Intercept)	27.05	25.47	28.63	33.54	<.001
Number of Successes	4.42	2.61	6.22	4.80	<.001
Ending Streak of Successes	-0.57	-2.14	0.99	0.72	.471
Exp 5 - Luck/Skill Attributions					
(Intercept)	15.51	14.65	16.38	35.18	<.001
Number of Successes	2.50	1.52	3.49	4.97	<.001
Ending Streak of Successes	0.70	-0.15	1.55	1.61	.108

Illusion of Control

Our methods and measures are similar to those of past research on the phenomenon known as the "illusion of control" (*24*, *52*). But as noted in the main text, past studies in this tradition rely on deception and do not examine the effect of performance feedback on predictions of events produced by a transparently random device. The studies that do manipulate rates of "success" (*25, 28*) typically rely on a paradigm involving participants attempting to control the illumination of a light by flipping a switch under varying reinforcement schedules (e.g., 50% of trials, 75% of trials). These studies have shown that higher rates of positive reinforcement (trials in which participants flip the switch and the light turns on) are associated with greater perceived control. As an examination of people's reactions to randomly determined performance these studies are limited because they involve an action that is commonly causally associated with its outcome. They do reveal how increasing the rate of action-outcome co-occurrence affects people's perception of the efficacy of their actions, but they do not tell us how performance transparently determined by chance affects people's beliefs and behaviors.

Perhaps more importantly, the evidentiary basis for the illusion of control is surprisingly unclear. As mentioned in the main text, illusion of control effects in the context of ostensibly random outcome generating mechanisms have been difficult to replicate in carefully controlled experiments (*44*, *53-57*). The most recent meta-analysis of illusion of control studies reported on data from 34 studies (*60*) and the authors report an overall mean effect size of D = .62, an impressively large effect size consistent with findings from an earlier meta-analysis (*61*).

However, a close inspection of the effect sizes and sample sizes of individual studies (data from Table 1 of *53*) shows a remarkable dependence of the former on the latter. As shown in *Figure S20*, almost all studies that show effect sizes larger than .50 are based on samples of less than 100 participants. Furthermore, there is a strong negative correlation between effect sizes and sample sizes, $r(34) = -.63$, $p < .001$, although the relationship appears to be non-linear. In fact, a simple power function with a negative exponent accounts for 68% of the variance of the effect sizes. We don't have information about the specific sample sizes of the control and treatment groups in the individual studies, but if we assume equal sample sizes, we can compute the SEs of the effect sizes and test for the presence of a bias, using the Egger's regression test (*62*) and a conservative estimate of the SEs (*63*). The Egger's test is highly significant. The intercept, that indicates the presence of bias, is 4.15 (95% CI: 2.95 – 5.35), $t(32) = 7.03$, $p < .001$. As the authors of the meta-analysis noted, the studies included in the analyses were very heterogeneous. The analyses here suggest that either these studies cover a variety of unrelated phenomena or that there are substantial biases in the selection of studies for publication.

Figure S20: Study effect size (Cohen's d) by sample size from the 34 studies included in Stefan & David, 2013.

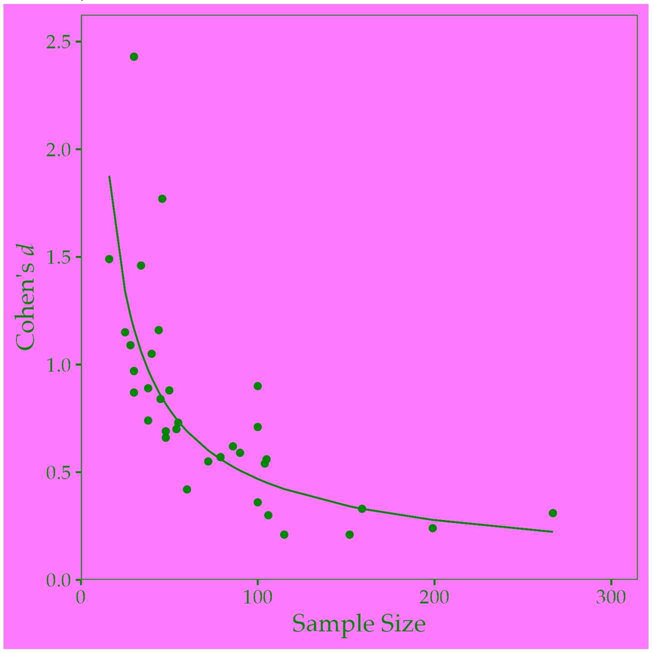

Tables with exact wording of all measures

Table S38. Measures of self-reported beliefs used in all experiments.

**Dependent Variable**	**Response Scale**	**Used in Experiment**
**Ability to Predict**		
Rate the degree to which you think you could accurately predict the outcomes if you continued to play the coin toss game:	0: No ability to predict – 100: Ability to predict outcomes perfectly	1,2,3,4,5
**Expected Number of Correct Predictions**		
Suppose that you continue to play the coin toss game for 20 more trials. On each trial you would predict the outcome (head or tail) in advance of the toss. Out of those 20 trials, on **how many trials would your prediction be correct?**		1*,2,3,4,5*
**Expected Number of Heads Outcomes**		
Suppose that you continue to play the coin toss game for 20 more trials. Out of those 20 trials, **on how many trials would a head be tossed?**		2,4,5*
**Luck and Skill Attributions**		
Your performance predicting the outcomes of the coin tosses can be described as a combination of luck and skill. Rate the degree to which you believe you were just lucky (or unlucky) versus the degree to which you have a skill that allows you to correctly predict the coin toss outcomes:	0: Totally Luck – 100: Totally skill	1,5: *Predict condition*
Your performance choosing between the two coins can be described as a combination of luck and skill. Rate the degree to which you believe you were just lucky (or unlucky) versus the degree to which you are skilled at choosing between coins:	0: Totally Luck – 100: Totally skill	5: *Choose condition*
Your performance tossing the coins can be described as a combination of luck and skill. Rate the degree to which you believe you were just lucky (or unlucky) versus the degree to which you are skilled at tossing the coin:	0: Totally Luck – 100: Totally skill	5: *Observe condition*

Rate the degree to which you believe **luck** (good luck OR bad luck) played a role in your performance in the task:	0: Luck played no role at all – 100: My outcomes were completely due to luck	2,3,4
Rate the degree to which you believe **skill** (or lack of skill) played a role in your performance in the task:	0: Skill played no role at all – 100: My outcomes were completely due to skill	2,3,4
**Advice**		
Imagine you are giving advice to someone else who is going to do the same coin toss prediction task you just completed. In 1-2 sentences, what would you tell them about how to accurately predict as many coin tosses as possible?		4
**Confidence in Advice**		
How confident are you that if someone followed your advice they would be successful in this task?	0: Not at all confident – 100: Totally confident	4
**Random vs. Determined Outcomes**		
Rate the degree to which you believe the outcomes (heads/tails) in this experiment resulted from a random, chance process:	0: Completely random – 100: Completely determined	1*,2,3,4,5*
**Individual Risk Preferences**		
How willing are you to take risks in general?	0: Not willing to take risks at all - 100: Very willing to take risks	2,3,4

*indicates measure was collected but not pre-registered

Table S39. Measures of risk taking used in all experiments.

**Risk measure**	**Response Scale**	**Used in Experiment**

You now have the opportunity to play the coin toss game again. Just like before, if you play it you may earn between $0.00 - $6.25 based on how many tosses you correctly predict. But, you can also choose to quit now, and take $1.00 for sure – we pay you the $1 right now, but you have no chance to play the coin toss game again.<br><br>Which option do you prefer: Play the coin toss game again and win between $0.00 and $6.25 or take the $1 for sure and quit for good?	0: Take the $1 for sure<br>1: Play the coin toss game again to win between $0.00 and $6.25	1

You will now **flip the same coin** five more times, predicting the outcome of each toss.<br><br>This time **you can be paid based on your ability** to predict the outcomes of the tosses. Which of the following payment options would you like to determine your payment?	0: You can be paid 50 points (25 cents) for sure and make the predictions with no additional pay<br><br>1: **Predict *at least* 1 out of 5 and earn 100** (50 cents) **points**: If you can predict at least 1 of the tosses correctly, you earn 100 (50 cents) points … if you cannot predict even one toss correctly you earn nothing<br><br>2: **Predict *at least* 2 and earn 200** ($1) **points** ... if you fail to predict at least 2 ($2) correctly you earn nothing.<br><br>3: **Predict *at least* 3 and earn 400** ($2) **points** ... if you fail to predict at least 3 correctly you earn nothing.<br><br>4: **Predict *at least* 4 and earn 800** ($4) **points** ... if you fail to predict at least 4 correctly you earn nothing.<br><br>5: **Predict *all* 5 and earn 1600** ($8) **points** … if you fail to predict all 5 correctly you earn nothing	2,3,4

Suppose you were offered the chance to continue playing this coin toss game 5 more times, and **you would win $1 for each head that was tossed**. This means you could win between $0 and $5.<br><br>Would you choose to play the game and gamble to win $0 to $5, or would you take a $2.50 payment for sure and not play the game?	0: Take $2.50 for sure –<br>100: Play the game to win between $0.00 - $5.00	5
Suppose you were offered a chance to continue playing this coin toss game five more times, and **you would win $1 for each toss on which you correctly predicted the outcome** (heads or tails) in advance of the toss.<br><br>In other words, you would be paid for the **accuracy** of your predictions.<br><br>This means you could win between $0 and $5. Would you choose to play the game and bet on your predictions to win $0 to $5, or would you take a $2.50 payment for sure and not play the game?	0: Take $2.50 for sure –<br>100: Play the game to win between $0.00 - $5.00	5

*indicates measure was collected but not pre-registered

Table S40. Four-item probability questionnaire used in Experiments 2, 3, 4.

Question	Answer Choices (incorrect; **correct**)
You toss a fair, six-sided die one time. Which of the following is true?	1) The result is more likely to be a "six" than a "one."<br>2) The result is more likely to be a "one" than a "six."<br>**3) The result is equally likely to be a "one" as it is to be a "six."**
You toss a fair coin 3 times. It comes up: **tails, tails, tails** three times in a row. Which of the following is true?	1) On the next flip, a tails result is more likely than a heads result.<br>2) On the next flip, a heads result is more likely than a tails result.<br>**3) On the next flip, a heads result and a tails result are equally likely.**
The chance of getting a rare disease is .03	1) 3

per person. Out of 1,000 people, how many of them are expected to get the disease?	2) 15<br>**3) 30**<br>4) 333
What is the probability that if you toss a fair coin twice, you will get one heads and one tails in any order?	1) 25%<br>**2) 50%**<br>3) 75%<br>4) 90%

Appendix A: Reference List of Studies of Judgments of Binary Sequences

Abramson, L. Y., & Alloy, L. B. (1980). Judgment of contingency: Errors and their implications. In A. Baum & J. Singer (Eds.), *Advances in Environmental Psychology* (Vol. 2, pp. 111–128). Psychology Press.

Alberoni, F. (1962). Contribution to the study of subjective probability: I. *Journal of General Psychology, 66*(1), 241.

Alberoni, F. (1962). Contribution to the study of subjective probability: Prediction II. *Journal of General Psychology, 66*(1), 265.

Altmann, E. M., & Burns, B. D. (2005). Streak biases in decision making: Data and a memory model. *Cognitive Systems Research*, 6(1), 5–16. https://doi.org/10.1016/j.cogsys.2004.09.002

Amir, G. S., & Williams, J. S. (1999). Cultural influences on children's probabilistic thinking. *Journal of Mathematical Behavior*, 18(1), 85–107.

Anderson, N. H. (1960). Effect of first-order conditional probability in a two-choice learning situation. *Journal of Experimental Psychology, 59*(2), 73-77.

Anderson, N. H. (1966). Test of a prediction of stimulus sampling theory in probability learning. *Journal of Experimental Psychology, 71*(4), 499-510.

Anderson, N. H., & Whalen, R. E. (1960). Likelihood judgments and sequential effects in a two-choice probability learning situation. *Journal of Experimental Psychology, 60*(2), 111-120.

Asparouhova, E., Hertzel, M., & Lemmon, M. (2009). Inference from streaks in random outcomes: Experimental evidence on beliefs in regime shifting and the law of small numbers. *Management Science, 55*(11), 1766–1782. https://doi.org/10.1287/mnsc.1090.1059

Ayton, P., & Fischer, I. (2004). The hot hand fallacy and the gambler's fallacy: Two faces of subjective randomness. *Memory & Cognition, 32*(8), 1369–1378.

Ball, C. T. (2012). Not all streaks are the same: Individual differences in risk preferences during runs of gains and losses. *Judgment and Decision Making, 7*(4), 452–461.

Barron, G., & Leider, S. (2010). The role of experience in the gambler's fallacy. *Journal of Behavioral Decision Making, 23*(2), 117–129. https://doi.org/10.1002/bdm.676

Benjamin, D. J., Moore, D. A., & Rabin, M. (2017). Biased beliefs about random samples: Evidence from two integrated experiments. *NBER Working Paper No. 23927.* https://doi.org/10.3386/w23927

Blinder, D. S., & Oppenheimer, D. M. (2008). Beliefs about what types of mechanisms produce random sequences. *Journal of Behavioral Decision Making, 21*(4), 414–427. https://doi.org/10.1002/bdm.596

Bogartz, R. S. (1965). Sequential dependencies in children's probability learning. *Journal of Experimental Psychology*, 70(4), 365–370. https://doi.org/10.1037/h0022372

Bou, S., Brandts, J., Cayón, M., & Guillén, P. (2016). The price of luck: paying for the hot hand of others. *Journal of the Economic Science Association, 2*, 60-72.

Boynton, D. M. (2003). Superstitious responding and frequency matching in the positive bias and gambler's fallacy effects. *Organizational Behavior and Human Decision Processes, 91*(1), 119–127. https://doi.org/10.1016/S0749-5978(03)00064-5

Bradley, A. E. (2024). *Three essays in experimental economics* (Doctoral dissertation). Virginia Tech.

Braga, J. N., Ferreira, M. B., Sherman, S. J., & Mata, A. (2018). What's next? Disentangling availability from representativeness using binary decision tasks. *Journal of Experimental Social Psychology, 76*, 307–319. https://doi.org/10.1016/j.jesp.2018.03.006

Brown, W. L., & Overall, J. E. (1959). Implications of recency effects for probability learning theories. *Journal of General Psychology, 61*, 243.

Budescu, D. V. (1987). A Markov model for generation of random binary sequences. *Journal of Experimental Psychology: Human Perception and Performance, 13*(1), 25–39.

Budescu, D. V., & Rapoport, A. (1994). Subjective randomization in one- and two-person games. *Journal of Behavioral Decision Making, 7*(4), 261–278.

Burns, B. D. (2003). *When it is adaptive to follow streaks: Variability and stocks*. In *Proceedings of the 25th Annual Conference of the Cognitive Science Society* (pp. 198–203).

Burns, B. D. (2004). Heuristics as beliefs and as behaviors: The adaptiveness of the "hot hand." *Cognitive Psychology*, 48(3), 295–331. https://doi.org/10.1016/j.cogpsych.2003.07.003

Burns, B. D., & Corpus, B. (2004). Randomness and inductions from streaks: "Gambler's fallacy" versus "hot hand." *Psychonomic Bulletin & Review*, 11(1), 179–184. https://doi.org/10.3758/BF03206480

Burns, B. D., & Vollmeyer, R. (1998). Modeling the adversary and success in competition. *Journal of Personality and Social Psychology*, 75(3), 711–718. https://doi.org/10.1037/0022-3514.75.3.711

Burns, B. D., & Wieth, M. (2004). The collider principle in causal reasoning: Why the Monty Hall dilemma is so hard. *Journal of Experimental Psychology: General*, 133(3), 434–449. https://doi.org/10.1037/0096-3445.133.3.434

Burns, B. D., Cox, C. R., & Sheridan, A. (2008). The role of mechanism in expectations about the future: Luck and skill. In *Proceedings of the 30th Annual Conference of the Cognitive Science Society* (pp. 1266–1271).

Carlson, K. A., & Shu, S. B. (2007). The rule of three: How the third event signals the emergence of a streak. *Organizational Behavior and Human Decision Processes, 104*(1), 113–121. https://doi.org/10.1016/j.obhdp.2007.03.004

Caruso, E. M., Waytz, A., & Epley, N. (2010). The intentional mind and the hot hand: Perceiving intentions makes streaks seem likely to continue. *Cognition, 116*(1), 149–153. https://doi.org/10.1016/j.cognition.2010.04.006

Chen, D. L., Moskowitz, T. J., & Shue, K. (2016). Decision making under the gambler's fallacy: Evidence from asylum judges, loan officers, and baseball umpires. *The Quarterly Journal of Economics, 131*(3), 1181–1242. https://doi.org/10.1093/qje/qjw017

Chi, D., & Burns, B. D. (2024). *Benford's law: Testing the effects of distributions and anchors on number estimation*. *Proceedings of the 46th Annual Meeting of the Cognitive Science Society*.

Chiesi, F., & Primi, C. (2009). Recency effects in primary-age children and college students. *International Electronic Journal of Mathematics Education*, 4(3), 259–274.

Chiesi, F., Primi, C., & Morsanyi, K. (2011). Developmental changes in probabilistic reasoning: The role of cognitive capacity, instructions, thinking styles, and relevant knowledge. *Thinking & Reasoning*, 17(3), 315–350. https://doi.org/10.1080/13546783.2011.598401

Craig, G. J., & Myers, J. L. (1963). A developmental study of sequential two-choice decision making. *Child Development*, 34(2), 483–493. https://doi.org/10.2307/1126743

Croson, R., & Sundali, J. (2005). The gambler's fallacy and the hot hand: Empirical data from casinos. *Journal of Risk and Uncertainty, 30*(3), 195–209. http://www.jstor.org/stable/41761194

Derks, P. L. (1962). The generality of the conditioning axiom in human binary prediction. *Journal of Experimental Psychology, 63*(6), 538–545.

Derks, P. L. (1963). Effect of run length on the gambler's fallacy. *Journal of Experimental Psychology, 65*(2), 213–214.

Derks, P. L., & Paclisanu, M. L. (1967). Simple strategies in binary prediction by children and adults. *Journal of Experimental Psychology*, 73(2), 278–284. https://doi.org/10.1037/h0024132

Diener, D., & Thompson, W. B. (1985). Recognizing randomness. *The American Journal of Psychology, 98*(3), 433–447. https://www.jstor.org/stable/1422628

Dohmen, T., Falk, A., Huffman, D., Marklein, F., & Sunde, U. (2009). Biased probability judgment: Evidence of incidence and relationship to economic outcomes from a representative sample. *Journal of Economic Behavior & Organization, 72*(3), 903–915. https://doi.org/10.1016/j.jebo.2009.07.014

Edwards, W. (1956). Reward probability, amount, and information as determiners of sequential two-alternative decisions. *Journal of Experimental Psychology, 52*(3), 177–189.

Edwards, W. (1961). Probability learning in 1000 trials. *Journal of Experimental Psychology, 62*(4), 385–394.

Falk, R., Falk, R., & Ayton, P. (2009). Subjective patterns of randomness and choice: Some consequences of collective responses. *Journal of Experimental Psychology: Human Perception and Performance, 35*(1), 203–224.

Farmer, G. D., Warren, P. A., & Hahn, U. (2017). Who "believes" in the gambler's fallacy and why? *Journal of Experimental Psychology: General, 146*(1), 63–76. https://doi.org/10.1037/xge0000245

Feldman, J. (1959). On the negative recency hypothesis in the prediction of a series of binary symbols. *The American Journal of Psychology, 72*(4), 597–599. https://www.jstor.org/stable/1419506

Fernberger, S. W. (1920). Interdependence of judgments within the series for the method of constant stimuli. *The American Journal of Psychology, 31*(1), 126–129.

Forlicz, M., Rólczyński, T., & Simonetti, B. (2023). Illusion of prediction possibility of random outcomes: Experimental results. *Quality & Quantity, 57*, S481–S495. https://doi.org/10.1007/s11135-022-01433-6

Gal, I., & Baron, J. (1996). Understanding repeated simple choices. *Thinking and Reasoning, 2*(1), 81–98.

Galanter, E. H., & Smith, W. A. S. (1958). Some experiments on a simple thought-problem. *The American Journal of Psychology, 71*(2), 359–366. https://doi.org/10.2307/1420080

Gault, R. H., & Goodfellow, L. D. (1940). Sources of error in psycho-physical measurements. *Journal of General Psychology, 22*, 197.

Gemelli, A., & Alberoni, F. (1961). Experimental studies of the concept of chance. *Journal of General Psychology, 65*, 3.

Gold, E., & Hester, G. (2008). The gambler's fallacy and the coin's memory. In J. I. Krueger (Ed.), *Rationality and Social Responsibility: Essays in Honor of Robyn Mason Dawes* (pp. 21–39). Psychology Press.

Goodfellow, L. D. (1940). The human element in probability. *Journal of General Psychology, 22*, 201.

Gronchi, G., & Sloman, S. A. (2021). Regular and random judgements are not two sides of the same coin: Both representativeness and encoding play a role in randomness perception. *Psychonomic Bulletin & Review, 28*(5), 1707–1714. https://doi.org/10.3758/s13423-021-01934-9

Gualtieri, S., & Denison, S. (2018). Exploring information use in children's decision-making: Base-rate neglect and trust in testimony. *Journal of Experimental Child Psychology*, 166, 314–329. https://doi.org/10.1016/j.jecp.2017.09.021

Hahn, U., & Warren, P. A. (2010). Why three heads are a better bet than four: A reply to Sun, Tweney, and Wang (2010). *Psychological Review*, *117*(2), 706–711. https://doi.org/10.1037/a0019037

Hake, H. W., & Hyman, R. (1953). Perception of the statistical structure of a random series of binary symbols. *Journal of Experimental Psychology, 45*(1), 64–72.

Hart, S. S., & Levin, I. P. (2002). Locus of control as a factor in the gambler's fallacy and the hot hand effect. *Journal of Behavioral Decision Making, 15*(1), 51–74.

Huettel, S. A., Mack, P. B., & McCarthy, G. (2002). Perceiving patterns in random series: Dynamic processing of sequence in prefrontal cortex. *Nature Neuroscience, 5*(5), 485–490. https://doi.org/10.1038/nn841

Jacobs, J. E., & Potenza, M. (1991). The use of judgment heuristics to make social and object decisions: A developmental perspective. *Child Development*, 62(1), 166–178. https://doi.org/10.1111/j.1467-8624.1991.tb01522.x

Jarvik, M. E. (1951). Probability learning and a negative recency effect in the serial anticipation of alternative symbols. *Journal of Experimental Psychology, 41*(4), 291–297.

Kareev, Y. (1992). Not that bad after all: Generation of random sequences. *Journal of Experimental Psychology Human Perception & Performance*, *18*(4), 1189–1194. https://doi.org/10.1037/0096-1523.18.4.1189

Koehler, D. J., & James, G. (2009). Probability matching in choice under uncertainty: Intuition versus deliberation. *Cognition, 113*(1), 123–137. https://doi.org/10.1016/j.cognition.2009.07.003

Lindman, H., & Edwards, W. (1961). Unlearning the gambler's fallacy. *Journal of Experimental Psychology, 62*(6), 630–631.

Lyons, J., Weeks, D. J., & Elliott, D. (2013). The gambler's fallacy: A basic inhibitory process? *Frontiers in Psychology, 4*(72), 1–8. https://doi.org/10.3389/fpsyg.2013.00072

Massey, C., & Wu, G. (2004). Online appendix to "Detecting regime shifts: The causes of under- and overreaction." [Supplementary Material].

Massey, C., & Wu, G. (2005). Detecting regime shifts: The causes of under- and overreaction. *Management Science, 51*(6), 932–947. https://doi.org/10.1287/mnsc.1050.0386

Matthews, L., & Sanders, W. (1984). Effects of causal and noncausal sequences of information on subjective prediction. *Psychological Reports*, 54(1), 211–222. https://doi.org/10.2466/pr0.1984.54.1.211

Matthews, W. J. (2013). Relatively random: Context effects on perceived randomness and predicted outcomes. *Journal of Experimental Psychology: Learning, Memory, and Cognition, 39*(5), 1642–1648. https://doi.org/10.1037/a0031081

Matthews, W. J., & Stewart, N. (2009). Psychophysics and the judgment of price: Judging complex objects on a non-physical dimension elicits sequential effects like those in perceptual tasks. *Judgment and Decision Making, 4*(1), 64–81.

McClelland, G. H., & Hackenberg, B. H. (1978). Subjective probabilities for sex of next child: U.S. college students and Philippine villagers. *Journal of Population, 1*(2), 132–147.

McDonald, F. E. J., & Newell, B. R. (2009). When a coin toss does not appear random: Causal belief and judgments of randomness. *Proceedings of the Annual Meeting of the Cognitive Science Society, 31*(31), 1580–1585. https://escholarship.org/uc/item/04w9q1hw

Militana, E., Wolfson, E., & Cleaveland, J. M. (2010). An effect of inter-trial duration on the gambler's fallacy choice bias. *Behavioural Processes, 84*(3), 455–459. https://doi.org/10.1016/j.beproc.2010.02.010

Morrison, R. S., & Ordeshook, P. C. (1975). Rational choice, light guessing, and the gambler's fallacy. *Public Choice, 22*, 79–89. https://doi.org/10.1007/BF01718987

Mossbridge, J. A., Roney, C. J. R., & Suzuki, S. (2015). Losses and external outcomes interact to produce the gambler's fallacy. *Cognition, 140*, 85–96. https://doi.org/10.1016/j.cognition.2015.03.012

Nickerson, R. S. (2007). *Penney Ante: Counterintuitive probabilities in coin tossing*. *The UMAP Journal*, 28(4), 503–532.

Nickerson, R. S., & Butler, S. F. (2009). On producing random binary sequences. *The American journal of psychology*, *122*(2), 141–151.

Nicks, D. C. (1959). Prediction of sequential two-choice decisions from event runs. *Journal of Experimental Psychology, 57*(2), 105-114.

Nies, R. C. (1962). Effects of probable outcome information on two-choice learning. *Journal of Experimental Psychology, 64*(5), 430-433.

Olivola, C. Y., & Oppenheimer, D. M. (2008). Randomness in retrospect: Exploring the interactions between memory and randomness cognition. *Psychonomic Bulletin & Review, 15*(5), 991–996. https://doi.org/10.3758/PBR.15.5.991

Oppenheimer, D. M., & Monin, B. (2009). The retrospective gambler's fallacy: Unlikely events, constructing the past, and multiple universes. *Judgment and Decision Making, 4*(5), 326–334.

Powdthavee, N., & Riyanto, Y. E. (2015). Would you pay for transparently useless advice? A test of boundaries of beliefs in folly of predictions. *The Review of Economics and Statistics*, *97*(2), 257–272. http://www.jstor.org/stable/43556173

Rabin, M. (2002). Inference by Believers in the Law of Small Numbers. *The Quarterly Journal of Economics*, *117*(3), 775–816. http://www.jstor.org/stable/4132489

Rabin, M., & Vayanos, D. (2010). The gambler's and hot-hand fallacies: Theory and applications. *The Review of Economic Studies, 77*(2), 730–778. https://doi.org/10.1111/j.1467-937X.2009.00582.x

Rapoport, A., & Budescu, D. V. (1997). Randomization in individual choice behavior. *Psychological Review, 104*(3), 603-617.

Reber, A. S., & Millward, R. B. (1968). Event observation in probability learning. *Journal of Experimental Psychology, 77*(2), 317–327. https://doi.org/10.1037/h0025748

Reimers, S., Donkin, C., & Le Pelley, M. E. (2018). Perceptions of randomness in binary sequences: Normative, heuristic, or both? *Cognition*, 172, 11–25. https://doi.org/10.1016/j.cognition.2017.11.002

Roney, C. J. R., & Sansone, N. (2015). Explaining the gambler's fallacy: Testing a gestalt explanation versus the "law of small numbers." *Thinking & Reasoning, 21*(2), 193–205. https://doi.org/10.1080/13546783.2014.942367

Roney, C. J. R., & Trick, L. M. (2009). Sympathetic magic and perceptions of randomness: The hot hand versus the gambler's fallacy. *Thinking & Reasoning, 15*(2), 197–210. https://doi.org/10.1080/13546780902847137

Roney, C. J. R., Trick, L. M., & Gilbert, D. T. (2003). Grouping and gambling: A Gestalt approach to understanding the gambler's fallacy. *Canadian Journal of Experimental Psychology, 57*(2), 69–75. https://doi.org/10.1037/h0087427

Scheibehenne, B., & Studer, B. (2014). A hierarchical Bayesian model of the influence of run length on sequential predictions. *Psychonomic Bulletin & Review, 21*(1), 211–217. https://doi.org/10.3758/s13423-013-0469-1

Scheibehenne, B., Wilke, A., & Todd, P. M. (2011). Expectations of clumpy resources influence predictions of sequential events. *Evolution and Human Behavior, 32*(5), 326–333. https://doi.org/10.1016/j.evolhumbehav.2010.11.003

Scholl, S. G., & Greifeneder, R. (2011). Disentangling the effects of alternation rate and maximum run length on judgments of randomness. *Judgment and Decision Making, 6*(6), 531–541.

Shanks, D. R., Tunney, R. J., & McCarthy, J. D. (2002). A re-examination of probability matching and rational choice. *Journal of Behavioral Decision Making, 15*(3), 233–250. https://doi.org/10.1002/bdm.413

Studer, B., Limbrick-Oldfield, E. H., & Clark, L. (2015). 'Put your money where your mouth is!': Effects of streaks on confidence and betting in a binary choice task. *Journal of Behavioral Decision Making, 28*(3), 277–291. https://doi.org/10.1002/bdm.1844

Sun, Y. (2004). Detecting the Hot Hand: an alternative model. *Proceedings of the Annual Meeting of the Cognitive Science Society*, *26*(26). https://escholarship.org/content/qt63g1s923/qt63g1s923.pdf?t=op2l74

Sun, Y., & Wang, H. (2010). Gambler's fallacy, hot hand belief, and the time of patterns. *Judgment and Decision Making*, *5*(2), 124–132. https://doi.org/10.1017/s193029750000098x

Sun, Y., & Wang, H. (2010). Perception of randomness: On the time of streaks. *Cognitive Psychology, 61*(3), 333–342. https://doi.org/10.1016/j.cogpsych.2010.07.001

Sun, Y., O'Reilly, R. C., Bhattacharyya, R., Smith, J. W., Liu, X., & Wang, H. (2015). Latent structure in random sequences drives neural learning toward a rational bias. *Proceedings of the National Academy of Sciences, 112*(11), 3788-3792. https://doi.org/10.1073/pnas.1422036112

Sun, Y., Tweney, R. D., & Wang, H. (2010). Occurrence and nonoccurrence of random sequences: Comment on Hahn and Warren (2009). *Psychological Review, 117*(2), 697–703. https://doi.org/10.1037/a0018994

Sun, Y., Tweney, R. D., & Wang, H. (2010). Postscript: Untangling the gambler's fallacy. *Psychological Review, 117*(2), 704–705. https://doi.org/10.1037/0033-295X.117.2.704

Tyszka, T., Markiewicz, Ł., Kubińska, E., Gawryluk, K., & Zielonka, P. (2017). A belief in trend reversal requires access to cognitive resources. *Journal of Cognitive Psychology, 29*(2), 202–216. https://doi.org/10.1080/20445911.2016.1245195

Tyszka, T., Zielonka, P., Dacey, R., & Sawicki, P. (2008). Perception of randomness and predicting uncertain events. *Thinking & Reasoning, 14*(1), 83–110. https://doi.org/10.1080/13546780701677669

Vulkan, N. (2000). An economist's perspective on probability matching. *Journal of Economic Surveys, 14*(1), 101–118.

Warren, P. A., Gostoli, U., Farmer, G. D., Boyle, M., El-Deredy, W., Howes, A., & Hahn, U. (2010). Assessing the "bias" in human randomness perception. *Proceedings of the 32nd Annual Conference of the Cognitive Science Society*, 3072–3077.

Warren, P., Gostoli, U., Farmer, G., Boyle, M., El-Deredy, W., Howes, A., & Hahn, U. (2014). Assessing the "bias" in human randomness perception. *Proceedings of the Annual Meeting of the Cognitive Science Society, 36*(36). https://escholarship.org/uc/item/54v9b286

Wilke, A., & Barrett, H. C. (2009). Supplementary material for "The hot hand phenomenon as a cognitive adaptation for clumped resources."

Wilke, A., & Barrett, H. C. (2009). The hot hand phenomenon as a cognitive adaptation to clumped resources. *Evolution and Human Behavior, 30*(3), 161–169. https://doi.org/10.1016/j.evolhumbehav.2008.11.004

Williams, J. J., & Griffiths, T. L. (2013). Why are people bad at detecting randomness? A statistical argument. *Journal of Experimental Psychology: Learning, Memory, and Cognition*, 39(5), 1473–1490. https://doi.org/10.1037/a0032397

Winefield, A. H. (1966). Negative recency and event-dependence. *Quarterly Journal of Experimental Psychology, 18*(1), 47–54. https://doi.org/10.1080/14640746608400006

Witte, R. S. (1964). Long-term effects of patterned reward schedules. *Journal of Experimental Psychology, 68*(6), 588–594.

Xiang, Y., Dorst, K., & Gershman, S. J. (2024). On the robustness and provenance of the gambler's fallacy. *Cognitive Science.*

Xue, G., He, Q., Lei, X., Chen, C., Liu, Y., Chen, C., Lu, Z., Dong, Q., & Bechara, A. (2012). The gambler's fallacy is associated with weak affective decision making but strong cognitive ability. *PLoS ONE, 7*(10), e47019. https://doi.org/10.1371/journal.pone.0047019

Xue, G., Lu, Z., Levin, I. P., & Bechara, A. (2011). An fMRI study of risk-taking following wins and losses: Implications for the gambler's fallacy. *Human Brain Mapping, 32*(2), 271–281.

Yu, R. Q., Osherson, D., & Zhao, J. (2017). Alternation blindness in the perception of binary sequences. *Cognitive Science Society Proceedings*.
