# Supplementary material for: People calibrate future expectations to past performance when predicting transparently random events
Source: PNAS Nexus. 2025 Aug 26;4(8):pgaf237. doi: 10.1093/pnasnexus/pgaf237 (PMC12378910; doi:10.1093/pnasnexus/pgaf237)
Supplement: pgaf237_Supplementary_Data [file pgaf237_supplementary_data.zip › Supplementary reference.docx]

58 Bar-Hillel M, Peer E, Acquisti A. 2014. “Heads or tails?”—a reachability bias in binary choice. *J Exp Psychol Learn Mem Cogn.* 40(6): 1656–1663.

59 Griffiths TL, Tenenbaum JB. 2003. Probability, algorithmic complexity, and subjective randomness. Proceedings of the Annual Meeting of the Cognitive Science Society (Vol. 25, No. 25); Boston, MA.

60 Stefan S, David D. 2013. Recent developments in the experimental investigation of the illusion of control. A meta-analytic review. *J Appl Soc Psychol.* 43(2):377–386.

61 Presson PK, Benassi VA. 1996. Illusion of control: a meta-analytic review. *J Soc Behav Pers.* 11(3):493–510.

62 Egger M, Smith GD, Schneider M, Minder C. 1997. Bias in metaanalysis detected by a simple, graphical test. *BMJ*. 315(7109): 629–634.

63 Pustejovsky JE, Rodgers MA. 2019. Testing for funnel plot asymmetry of standardized mean differences. *Res Synth Methods.* 10(1):57–71.
